# External validation of machine learning models—registered models and adaptive sample splitting

**DOI:** 10.1093/gigascience/giaf036

**Published:** 2025-05-14

**Authors:** Giuseppe Gallitto, Robert Englert, Balint Kincses, Raviteja Kotikalapudi, Jialin Li, Kevin Hoffschlag, Ulrike Bingel, Tamas Spisak

**Affiliations:** Center for Translational Neuro- and Behavioral Sciences (C-TNBS), University Medicine Essen, Hufelandstraße 55, 45147, Essen, Germany; Department of Neurology, University Medicine Essen, Hufelandstraße 55, 45147, Essen, Germany; Center for Translational Neuro- and Behavioral Sciences (C-TNBS), University Medicine Essen, Hufelandstraße 55, 45147, Essen, Germany; Department of Diagnostic and Interventional Radiology and Neuroradiology, University Medicine Essen, Hufelandstraße 55, 45147, Essen, Germany; Center for Translational Neuro- and Behavioral Sciences (C-TNBS), University Medicine Essen, Hufelandstraße 55, 45147, Essen, Germany; Department of Neurology, University Medicine Essen, Hufelandstraße 55, 45147, Essen, Germany; Center for Translational Neuro- and Behavioral Sciences (C-TNBS), University Medicine Essen, Hufelandstraße 55, 45147, Essen, Germany; Department of Neurology, University Medicine Essen, Hufelandstraße 55, 45147, Essen, Germany; Center for Translational Neuro- and Behavioral Sciences (C-TNBS), University Medicine Essen, Hufelandstraße 55, 45147, Essen, Germany; Department of Neurology, University Medicine Essen, Hufelandstraße 55, 45147, Essen, Germany; Max Planck Institute for Human Cognitive and Brain Sciences, Max Planck School of Cognition, Stephanstraße 1A, D-04103, Leipzig, Germany; Center for Translational Neuro- and Behavioral Sciences (C-TNBS), University Medicine Essen, Hufelandstraße 55, 45147, Essen, Germany; Department of Neurology, University Medicine Essen, Hufelandstraße 55, 45147, Essen, Germany; Center for Translational Neuro- and Behavioral Sciences (C-TNBS), University Medicine Essen, Hufelandstraße 55, 45147, Essen, Germany; Department of Neurology, University Medicine Essen, Hufelandstraße 55, 45147, Essen, Germany; Center for Translational Neuro- and Behavioral Sciences (C-TNBS), University Medicine Essen, Hufelandstraße 55, 45147, Essen, Germany; Department of Diagnostic and Interventional Radiology and Neuroradiology, University Medicine Essen, Hufelandstraße 55, 45147, Essen, Germany

**Keywords:** machine learning, predictive modeling, preregistration, external validation, adaptive splitting

## Abstract

**Background:**

Multivariate predictive models play a crucial role in enhancing our understanding of complex biological systems and in developing innovative, replicable tools for translational medical research. However, the complexity of machine learning methods and extensive data preprocessing and feature engineering pipelines can lead to overfitting and poor generalizability. An unbiased evaluation of predictive models necessitates external validation, which involves testing the finalized model on independent data. Despite its importance, external validation is often neglected in practice due to the associated costs.

**Results:**

Here we propose that, for maximal credibility, model discovery and external validation should be separated by the public disclosure (e.g., preregistration) of feature processing steps and model weights. Furthermore, we introduce a novel approach to optimize the trade-off between efforts spent on model discovery and external validation in such studies. We show on data involving more than 3,000 participants from four different datasets that, for any “sample size budget,” the proposed adaptive splitting approach can successfully identify the optimal time to stop model discovery so that predictive performance is maximized without risking a low-powered, and thus inconclusive, external validation.

**Conclusion:**

The proposed design and splitting approach (implemented in the Python package “AdaptiveSplit”) may contribute to addressing issues of replicability, effect size inflation, and generalizability in predictive modeling studies.

## Introduction

Multivariate predictive models integrate information across multiple variables to construct predictions of a specific outcome and hold promise for delivering more accurate estimates than traditional univariate methods [1]. For instance, when predicting individual behavioral and psychometric characteristics from brain data, such models can provide higher statistical power and better replicability than conventional mass-univariate analyses [2]. Predictive models can utilize a variety of algorithms, ranging from simple linear-regression-based models to complex deep neural networks. With increasing model complexity, the model will be more prone to overfit its training dataset, resulting in biased, overly optimistic in-sample estimates of predictive performance and often decreased generalizability to data not seen during model fit [3]. Internal validation approaches, such as cross-validation (cv), provide the means for an unbiased evaluation of predictive performance during model discovery by repeatedly holding out parts of the discovery dataset for testing purposes [4, 5]. However, internal validation approaches, in practice, still tend to yield overly optimistic performance estimates [6–8]. There are several reasons for this kind of effect size inflation. First, predictive modeling approaches typically display a high level of “analytical flexibility” and pose a large number of possible methodological choices in terms of feature preprocessing and model architecture, which emerge as uncontrolled (e.g., not cross-validated) “hyperparameters” during model discovery. Seemingly “innocent” adjustments of such parameters can also lead to overfitting if this happens outside the cv loop. The second reason for inflated internally validated performance estimates is “leakage” of information from the test dataset to the training dataset [9]. Information leakage has many faces. It can be a consequence of, for instance, feature standardization in a non-cv-compliant way or, in medical imaging, the co-registration of brain data to a study-specific template. Therefore, it is often very hard to notice, especially in complex workflows. Another reason for overly optimistic internal validation results may be that even the highest-quality discovery datasets can only yield an imperfect representation of the real world. Therefore, predictive models might capitalize on associations that are specific to the dataset at hand and simply fail to generalize “out-of-the-distribution,” e.g., to different populations. Finally, some models might also be overly sensitive to unimportant characteristics of the training data, such as subtle differences between batches of data acquisition or center-effects [10, 11].

The obvious solution for these problems is external validation; that is, to evaluate the model's predictive performance on independent (“external”) data that are guaranteed to be unseen throughout the whole model discovery procedure. There is a clear agreement in the community that external validation is critical for establishing machine learning model quality [2, 5, 12–14]. However, the amount of data to be used for model discovery and external validation can have crucial implications on the predictive power, replicability, and validity of predictive models, and is, therefore, subject of intense discussion [2, 15–19] (Supplementary Table 1). Finding the optimal sample sizes is especially challenging for biomedical research, where this trade-off needs to weigh-in ethical and economic considerations. As a consequence, to date only around 10% of predictive modeling studies include an external validation of the model [20]. Those few studies performing true external validation often perform it on retrospective data (e.g., [21, 22]) or in separate, prospective studies [22, 23]. Both approaches can result in a suboptimal use of data and may slow down the dissemination process of new results.

In this manuscript we argue that maximal reliability and transparency during external validation can be achieved with prospective data acquisition preceded by “freezing” and publicly depositing (e.g., preregistering) the whole feature processing workflow and all model weights. Furthermore, we present a novel adaptive design for predictive modeling studies with prospective data acquisition that optimizes the trade-off between efforts spent on model discovery and external validation. We evaluate the proposed approach on data involving more than 3,000 participants from four different datasets to illustrate that for any “sample size budget,” it can successfully identify the optimal time to stop model discovery, so that predictive performance is maximized without risking a low-powered, and thus inconclusive, external validation.

## Background

### The anatomy of a prospective predictive modeling study

Let us consider the following scenario: a research group plans to involve a fixed number of participants in a study with the aim of constructing a predictive model, and at the same time, evaluate its external validity. How many participants should they allocate for model discovery, and how many for external validation, to get the highest-performing model as well as conclusive validation results?

**Figure 1:**
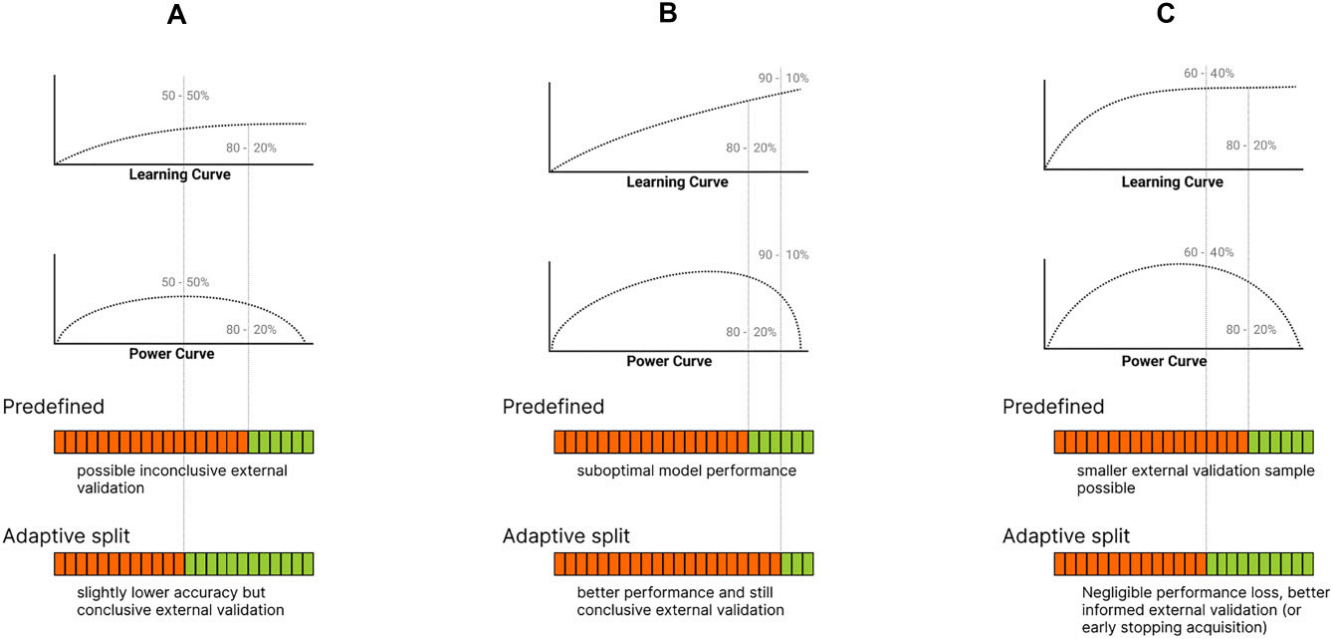
Examples of different optimal discovery and external validation sample sizes compared to a predefined 80%:20% Pareto split. (A) If the planned sample size and the model performance is low, the predefined external validation sample size might provide low statistical power to detect a significant model performance. (B) External validation of highly accurate models is well powered; increasing the discovery sample size (against the external validation sample size) might result in a better-performing final model. (C) Continuing training on the plateau of the learning curve will result in a negligible or biologically not relevant model performance improvement. In this case, a larger external validation sample (for more robust external performance estimates) or “early stopping” of the data acquisition process might be desirable.

In most cases it is very hard to make an educated guess about the optimal split of the total sample size into discovery and external validation samples prior to data acquisition. A possible approach is to use simplistic rules-of-thumb. Splitting data with an 80%:20% ratio (a.k.a. the Pareto split [24]) is probably the most common method, but a 90%:10% or a 50%:50% may also be plausible choices [25]. However, as illustrated on Fig. 1, such prefixed sample sizes are likely suboptimal in many cases and the optimal strategy is actually determined by the dependence of the model performance on training sample size, that is, the “learning curve.” For instance, in case of a significant but generally low model performance (Fig. 1A: flat learning curve) the model does not benefit a lot from adding more data to the discovery set but, on the other hand, it may require a larger external validation set for conclusive evaluation, due to the lower predictive effect size. This is visualized by the “power curve” in Fig. 1, which shows the statistical power of external validation with the remaining samples as a function of sample size used for model discovery. The optimal strategy will be different, however, if the learning curve shows a persistent increase, without a strong saturation effect, meaning that predictive performance can be significantly enhanced by training the model on a larger sample size (Fig. 1B). In this case, the stronger predictive performance that can be achieved with a larger training sample size, at the same time, allows a smaller external validation sample to be still conclusive. Finally, in some situations, model performance may rapidly get strong and reach a plateau at a relatively low sample size (Fig. 1C). In such cases, the optimal strategy might be to stop early with the discovery phase and allocate resources for a more powerful external validation.

### Transparent reporting of external validation: registered models

**Figure 2:**
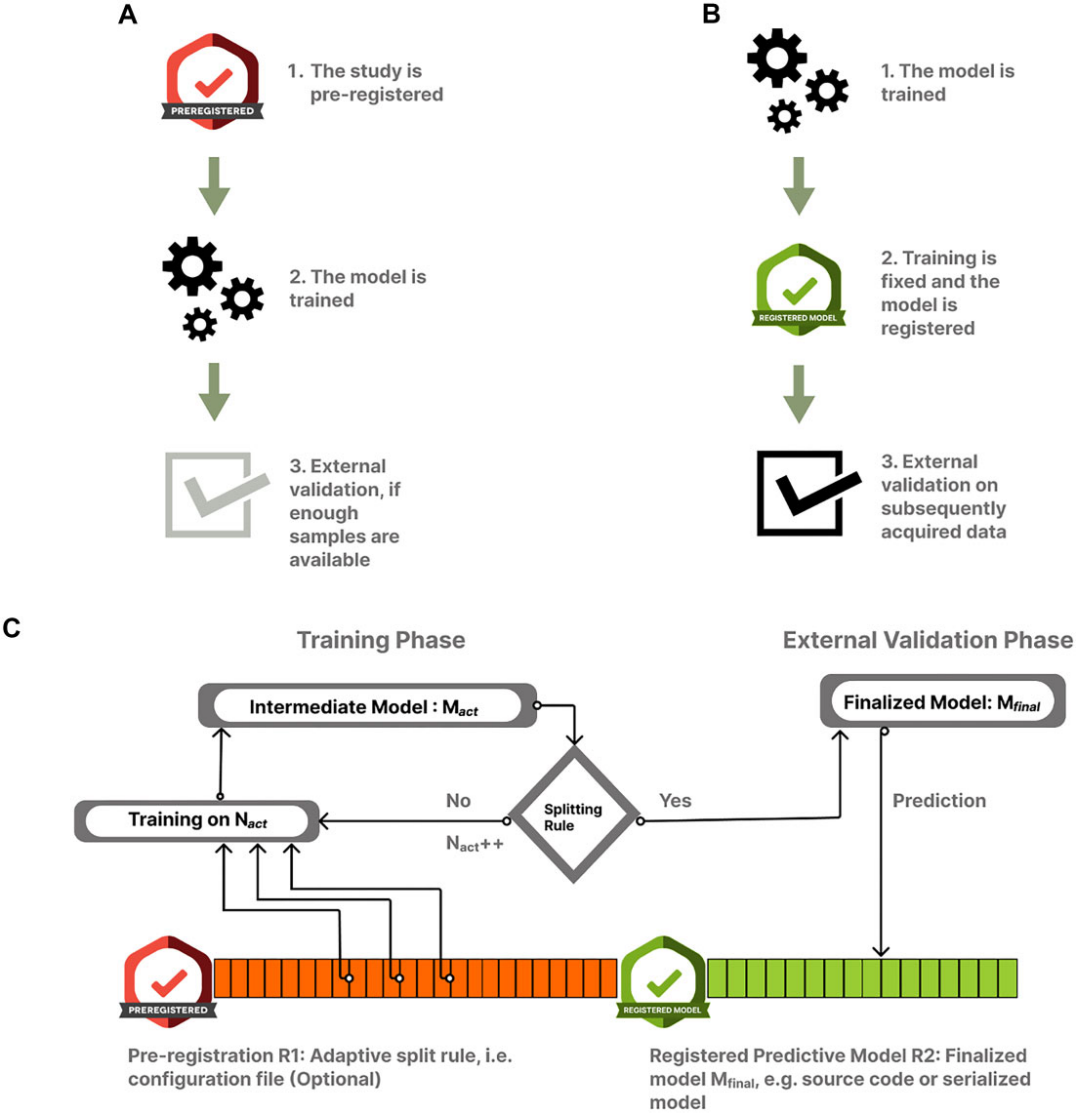
The registered model design and the proposed adaptive sample splitting procedure for prospective predictive modeling studies. (A) Predictive modeling combined with conventional preregistration. In this case the preregistration precedes data acquisition and requires fixing as many details of the analysis as possible. Given the potentially large number of coefficients to be optimized and the importance of hyperparameter optimization, conventional preregistration exhibits limited compatibility with predictive modeling studies. (B) Here we propose that in case of predictive modeling studies, public registration should only happen after the model is trained and finalized. The registration step in this case includes publicly depositing the finalized model, with all its parameters as well as all feature preprocessing steps. External validation is performed with the resulting “registered model.” This practice ensures a transparent, clear separation of model discovery and external validation. (C) The “registered model” design allows a flexible, adaptive splitting of the “sample size budget” into discovery and external validation phases. The proposed adaptive sample splitting procedure starts with fixing (and potentially preregistering) a stopping rule (R1). During the discovery phase, one or more candidate models are trained and the splitting rule is repeatedly evaluated as the data acquisition proceeds. When the splitting rule “activates,” the model gets finalized (e.g., by being fit on the whole training sample) and publicly deposited/registered (R2). Finally, data acquisition continues and the prospective external validation is performed on the newly acquired data.

A key criterion for external validation is the independence of the external data from the data used during model discovery [2, 12, 26]. Regardless of the splitting strategy, an externally validated predictive modeling study must provide strong guarantees for this independence criterion. Preregistration, i.e., the public disclosure of study plans before the start of the study, is an increasingly popular way of enhancing transparency and replicability in biomedical research [2, 27] (Fig. 2A), which could also be used to ensure the independence of the external validation sample.

However, as the concept of preregistration was originally developed for confirmatory research, it does not fit well with the exploratory nature of the model discovery phase in typical predictive modeling endeavors. Specifically, while preregistration necessitates that as many parameters of the analysis as possible are fixed before data acquisition, predictive modeling studies often involve a large number of hyperparameters (model architecture, feature preprocessing steps, regularization parameters, etc.) that are not known in advance and need to be optimized during the model discovery phase. This is especially true for complex machine learning models, such as deep neural networks, where the number of free parameters can easily reach tens of thousands or even more. In such cases, the preregistration of the discovery phase would require a large number of assumptions or simplifications, which would make the process ineffective and less transparent.

Therefore, we propose to perform the preregistration after the model discovery phase, but before the external validation (Fig. 2B). In this case, more freedom is granted for the discovery phase, while the external validation remains equally conclusive as long as the preregistration of the external validation includes all details of the finalized model (including the feature preprocessing workflow). This can easily be done by attaching the data and the reproducible analysis code used during the discovery phase or, alternatively, a serialized version of the fitted model (i.e., a file that contains all model weight). We refer to such models as “registered models.” Although preregistered external validation is, to date, sparse in the predictive modeling literature [20], examples of studies using the proposed registered model design do exist (e.g., [22, 23]). Such studies substantiate that the registered model approach allows model discovery with low sample sizes (*n* = 39 and *n* = 25 in the two studies, respectively) and still offer an unbiased evaluation of replicability and out-of-sample generalizability, without the need for data from thousands of individuals (as recently recommended by [16]).

### The adaptive splitting design

Even with registered models, the amount of data to be used for model discovery and external validation can have crucial implications for the predictive power, replicability and validity of predictive models. Here, we introduce a novel design for prospective predictive modeling studies that leverages the flexibility of model discovery granted by the registered model design. Our approach aims to adaptively determine an optimal splitting strategy during data acquisition. This strategy balances the model performance and the statistical power of the external validation (Fig. 2C). The proposed design involves continuous model fitting and hyperparameter tuning throughout the discovery phase, e.g., after every 10 new participants, and evaluating a “stopping rule” to determine if the desired compromise between model performance and the statistical power of the external validation has been achieved. This marks the end of the discovery phase and the start of the external validation phase, as well as the point at which the model must be publicly and transparently deposited or preregistered. Importantly, the preregistration should precede the continuation of data acquisition, i.e., the start of the external validation phase. In the present work, we propose and evaluate a concrete, customizable implementation for the splitting rule.

### Methods and Implementation

#### Components of the stopping rule

The stopping rule of the proposed adaptive splitting design can be formalized as function *S*:


(1) \begin{eqnarray*}
{S_{\mathrm{\Phi }}}\left( {{X_{act}},{y_{act}},\mathcal{M}} \right)\quad \quad S:{R^2} \to \left\{ {\textit{True},\textit{False}} \right\}
\end{eqnarray*}

where Φ denotes customizable parameters of the rule (detailed in the next paragraph), ${X_{act}} \in {R^2}$ is the data (a matrix consisting of ${n_{act}} > 0$ observations and a fixed number of features *p*) and ${y_{act}} \in R$ is the prediction target, as acquired so far and $\mathcal{M}$ is the machine learning model to be trained. The discovery phase ends if and only if the stopping rule returns $True$.

##### Hard sample size thresholds

Our stopping rule is designed so that it can force a minimum size for both the discovery and the external validation samples, ${t_{min}}$ and ${v_{min}}$, both being free parameters of the stopping rule.

Specifically:


(2) \begin{eqnarray*}
{\mathrm{Min\mbox{-}rule:}}\quad {n_{act}} \ge {t_{min}}
\end{eqnarray*}

(3) \begin{eqnarray*}
{\mathrm{Max\mbox{-}rule:}}\quad {n_{act}} \ge {n_{\textit{total}}} - {v_{min}}
\end{eqnarray*}

where ${n_{act}}$ and ${n_{\textit{total}}}$ are the actual sample size (e.g., participants measured so far) and the total sample size (i.e., the “sample size budget”), respectively, so that ${n_{\textit{total}}} \ge {n_{act}} > 0$. Setting ${t_{min}}$ and ${v_{min}}$ may be useful to prevent early stopping at the beginning of the training procedure, where predictive performance and validation power estimates are not yet reliable due to the small ${n_{act}}$ or to ensure that a minimal validation sample size, even if stopping criteria are never met. If ${t_{min}}$ and ${v_{min}}$ are set so that ${t_{min}} + {v_{min}} = {n_{\textit{total}}}$, then our approach falls back to training a registered model with predefined discovery and validation sample sizes.

##### Forecasting predictive performance via learning curve analysis

Taking internally validated performance estimates of the candidate model as a function of training sample size, also known as learning curve analysis, is a widely used approach to gain deeper insights into model discovery dynamics (see examples on Fig. 1). In the proposed stopping rule, we will rely on learning curve analysis to provide estimates of the current predictive performance and the expected gain when adding new data to the discovery sample.

Performance estimates can be unreliable or noisy in many cases, e.g., with low sample sizes or when using leave-one-out cross-validation [28]. To obtain stable and reliable learning curves, we propose to calculate multiple cross-validated performance estimates from subsamples sampled without replacement from the actual dataset. The proposed procedure is detailed in Algorithm 1.

**Algorithm 1:** (Bootstrapped Learning Curbe Analysis)

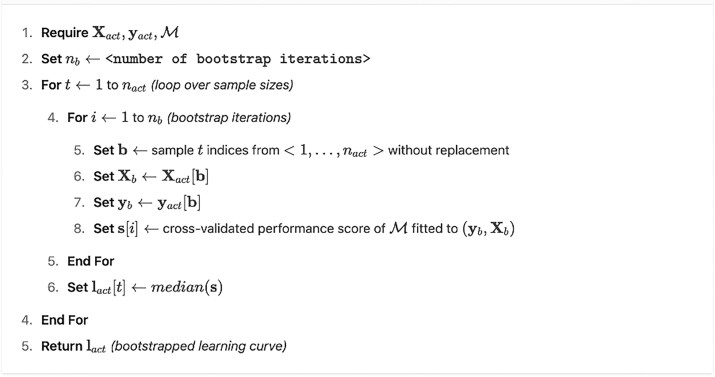

The learning curve analysis allows the discovery phase to be stopped if the expected gain in predictive performance is lower than a predefined relevance threshold and can be used, for instance, for stopping model training earlier in well-powered experiments and retaining more data for the external validation phase. Specifically, the stopping rule *S* will return $True$ if the Min-rule (equation (2)) is $True$ or the following is true:


(4) \begin{eqnarray*}
{\mathrm{Performance\mbox{-}rule:}}\quad \widehat {{s_{\textit{total}}}} - {s_{act}} \le {s_{min}}
\end{eqnarray*}

where ${s_{act}}$ is the actual bootstrapped predictive performance score (i.e., the last element of ${l_{act}}$, as returned by Algorithm 1, $\widehat {{s_{\textit{total}}}}$ is a estimate of the (unknown) predictive performance ${s_{\textit{total}}}$ (i.e., the predictive performance of the model trained on the whole sample size), and ${s_{min}}$ is the smallest predictive effect of interest. Note that this parameter configuration essentially switches off the performance rule for our main analysis (${s_{min}} = 0$, but see Supplementary  Fig. 7, for an analysis of the effect of the performance rule) and ensures that even in case of very small simulated sample size budgets, the training sample is suitable for cross-validation ($v\_min = \,\,12$).

While ${s_{\textit{total}}}$ is typically unknown at the time of evaluating the stopping rule *S*, there are various approaches to obtaining an estimate $\widehat {{s_{\textit{total}}}}$. In the base implementation of AdaptiveSplit, we stick to a simple method: we extrapolate the learning curve ${l_{act}}$ based on its tangent line at ${n_{act}}$, i.e., assuming that the latest growth rate will remain constant for the remaining samples. Although in most scenarios this is an overly optimistic estimate, it still provides a useful upper bound for the maximally achievable predictive performance with the given sample size and can successfully detect if the learning curve has already reached a flat plateau (as in Fig. 1C).

##### Statistical power of the external validation sample

Even if the learning curve did not reach a plateau, we still need to make sure that we stop the discovery phase early enough to save a sufficient amount of data for a successful external validation from our sample size budget. Given the actual predictive performance estimate ${s_{act}}$ and the size of the remaining, to-be-acquired sample ${s_{\textit{total}}} - {s_{act}}$, we can estimate the probability that the external validation correctly rejects the null hypothesis (i.e., zero predictive performance). This type of analysis, known as power calculation, allows us to determine the optimal stopping point that guarantees the desired statistical power during the external validation. Specifically, the stopping rule *S* will return $True$ if the Performance-rule (equation (4)) is $False$ and the following is true:


(5) \begin{eqnarray*}
{\mathrm{Power\mbox{-}rule:\,\,}}PO{W_{\mathrm{\alpha }}}\left( {{s_{act}},{n_{val}}} \right) \le {v_{pow}}
\end{eqnarray*}

where $PO{W_{\mathrm{\alpha }}}( {s,n} )$ is the power of a validation sample of size *n* to detect an effect size of *s*, and ${n_{val}} = {n_{\textit{total}}} - {n_{act}}$ is the size of the validation sample if stopping, i.e., the number of remaining (not yet measured) participants in the experiment. Given that machine learning model predictions are often non-normally distributed [11], our implementation is based on a bootstrapped power analysis for permutation tests, as shown in Algorithm 2. Our implementation is, however, simple to extend with other parametric or non-parametric power calculation techniques.

**Algorithm 2:** (Calculation of the Power-rule)

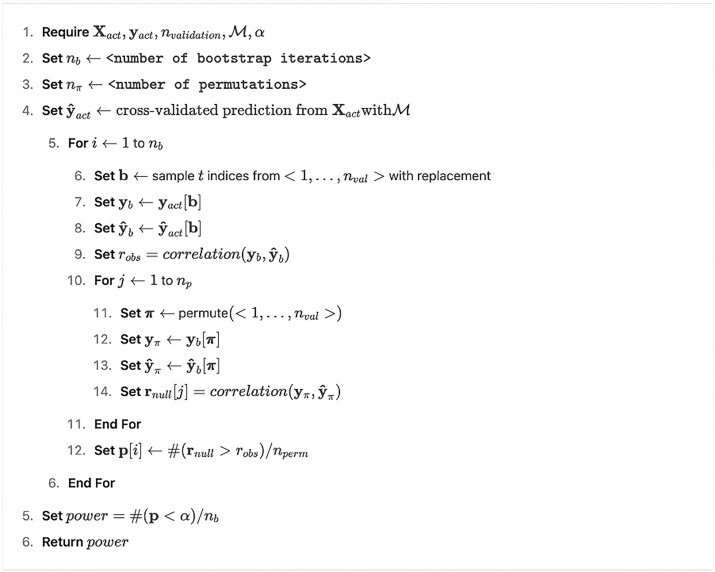

Note that depending on the aim of external validation, the Power-rule can be swapped to, or extended with, other conditions. For instance, if we are interested in accurately estimating the predictive effect size, we could condition the stopping rule on the width of the confidence interval for the prediction performance.

Calculating the validation power (Algorithm 2) for all available sample sizes ($n = 1 \ldots {n_{act}}$) defines the so-called “validation power curve” (see Fig. 1 and Supplementary Figs 2, 4 and 6), which represents the expected ratio of true positive statistical tests on increasing sample size calculated on the external validation set. Various extrapolations of the power curve can predict the expected stopping point during the course of the experiment.

#### Stopping rule

Our proposed stopping rule integrates the ${\mathrm{Min\mbox{-}rule}}$, the ${\mathrm{Max\mbox{-}rule}}$, the ${\mathrm{Performance\mbox{-}rule}}$ and the ${\mathrm{Power\mbox{-}rule}}$ in the following way:


(6) \begin{eqnarray*}
\begin{array}{@{}*{1}{l}@{}} {{S_{\mathrm{\Phi }}}\left( {{X_{act}},{y_{act}},\mathcal{M}} \right) = {\mathrm{Min\mbox{-}rule}}\quad AND}\\ {\quad \quad \quad \quad \quad \quad \quad \quad (}\\ {\quad \quad \quad \quad \quad \quad \quad \quad \quad {\mathrm{Max\mbox{-}rule}}\quad OR}\\ {\quad \quad \quad \quad \quad \quad \quad \quad \quad {\mathrm{Performance\mbox{-}rule}}\quad OR}\\ {\quad \quad \quad \quad \quad \quad \quad \quad \quad {\mathrm{Power\mbox{-}rule}}}\\ {\quad \quad \quad \quad \quad \quad \quad \quad )} \end{array}
\end{eqnarray*}

where ${\mathrm{\Phi }} \le {t_{min}},{v_{min}},{s_{min}},{v_{pow}},{\mathrm{\alpha }} > $ are the parameters of the stopping rule: minimum training sample size, minimum validation sample size, minimum effect of interest, target power for the external validation and the significance threshold, respectively.

We have implemented the proposed stopping rule in the Python package AdaptiveSplit [29]. The package can be used together with a wide variety of machine learning tools and provides an easy-to-use interface to work with scikit-learn [30] models.

#### Empirical evaluation

**Figure 3:**
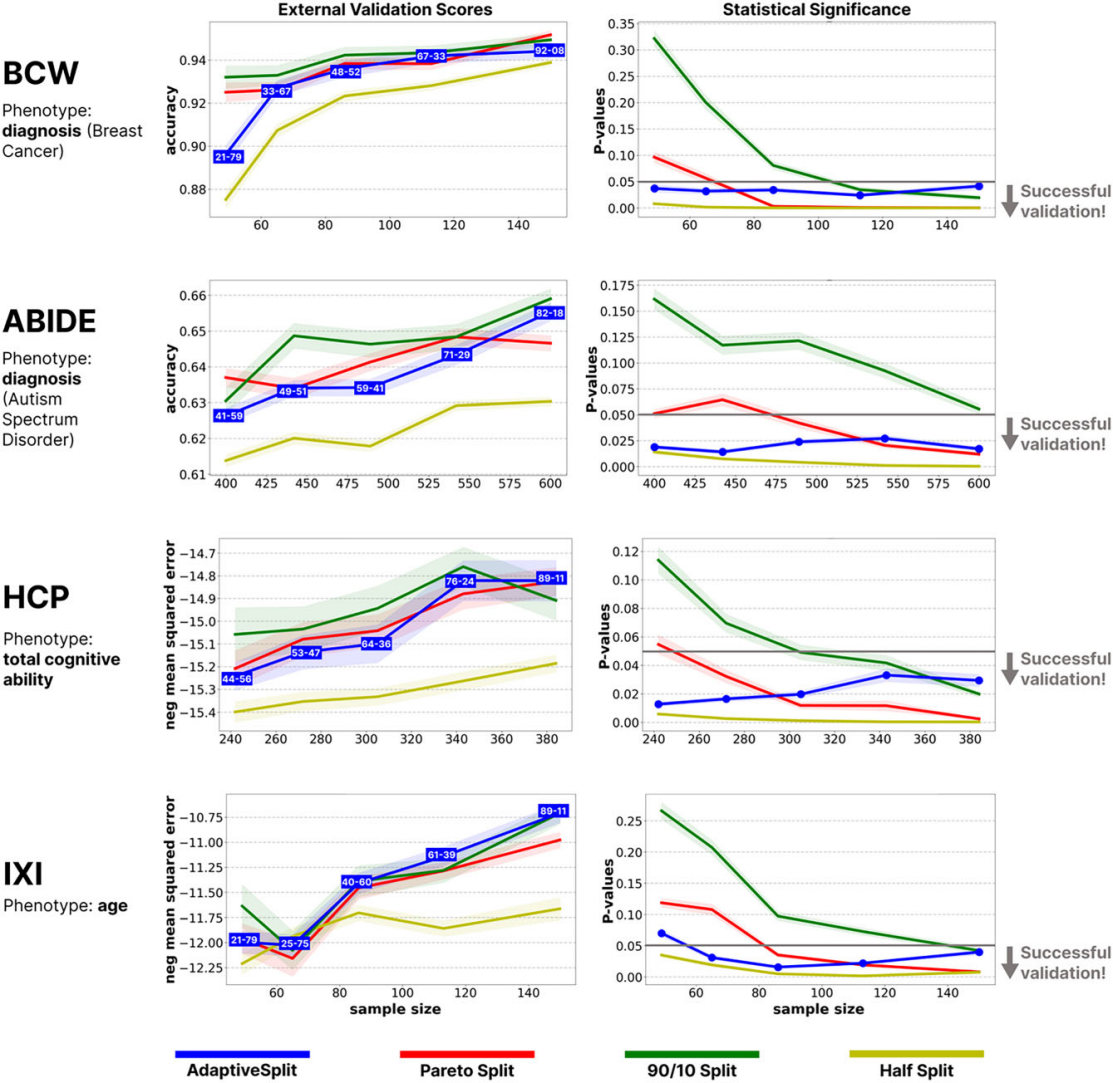
The proposed adaptive splitting approach provides a good compromise between predictive performance and the statistical power of the external validation. The left and right column show the comparison of splitting methods on external validation performance and *P*-values, respectively, at various ${n_{\textit{total}}}$. Confidence intervals are based on 100 repetitions of the analyses. The adaptive splitting approach (blue) provides a good compromise between predictive performance and statistical power of the external validation. The Pareto split (red) provides similar external validation performances to adaptive splitting; however, it often fails to provide conclusive results due to an insufficient sample size during external validation, especially in case of a limited sample size budget. The 90%:10% split (green) provides only slightly higher performances than the Pareto and the adaptive splitting techniques, but it very often gives inconclusive results ($P \ge 0.05$) in the external validation sample. Half-split (yellow) tends to provide worse predictive performance due to the too small discovery sample.

We evaluate the proposed stopping rule, as implemented in the package AdaptiveSplit [29], in four publicly available datasets; the Autism Brain Imaging Data Exchange (ABIDE) [31], the Human Connectome Project (HCP) [32], the Information eXtraction from Images (IXI) [33], and the Breast Cancer Wisconsin (BCW) [34] datasets (Fig. 3).

##### ABIDE

We obtained preprocessed data from the ABIDE dataset [31] involving the resting-state data of 866 participants (autism spectrum disorder, 402; neurotypical control, 464). Preprocessed regional time-series data were obtained as shared by [35], which were based on image data provided by the Pre-processed Connectome Project [36], preprocessed using the C-PAC pipeline [37, 38], without global signal regression. Tangent correlation across the time series of the *n* = 122 regions of the BASC brain parcellation (multilevel bootstrap analysis of stable clusters [39]) was computed with nilearn [40]. The resulting functional connectivity estimates were considered features for a predictive model of autism diagnosis.

##### HCP

The HCP dataset contains imaging and behavioral data of approximately 1,200 healthy subjects [32]. Preprocessed resting-state functional magnetic resonance imaging (fMRI) connectivity data (partial correlation of the mean regional time series of 100 brain parcels derived via independent component analysis [41], as published with the HCP1200 release (*n* = 999 participants with functional connectivity data)) were used to build models that predict individual fluid intelligence scores (Gf), measured with Penn progressive matrices [42]. The minimal preprocessing pipelines for structural, functional, and diffusion MRI were developed by the HCP and included spatial artifact/distortion removal, surface generation, cross-modal registration, and alignment to standard space [41]. These pipelines were specially designed to capitalize on the high-quality data offered by the HCP.

##### IXI

The IXI dataset is published by the Neuroimage Analysis Center, Imperial College London, UK, and is part of the project Brain Development. It consists of approximately 600 structural MRI images from a diverse population of healthy individuals, including both males and females across a wide age range. The dataset contains high-resolution brain images from three different MRI scanners (Philips Intera 3T, Philips Gyroscan Intera 1.5T, and GE 1.5T) and associated demographic information, making it suitable for studying age-related changes in brain structure and function. Structural preprocessing of T1-weighted images was conducted using FreeSurfer [43] software (version 6.0), run with default parameters, focusing on gray matter volume. The procedure included motion correction, skull stripping, removal of the cerebellum and brain stem, intensity correction, segmentation, tessellation, smoothing, and topology correction [44]. The cortical volume of brain regions was measured using the Desikan–Killiany brain atlas [45], producing 68 regional volume measures (34 per hemisphere, measured in mm^3^).

##### BCW

The BCW [34] dataset contains diagnostic features computed from digitized images of fine-needle aspirates (FNAs) of breast masses. The FNA procedure involves using a thin, hollow needle to extract cells from a suspicious area of breast tissue. These cells are then smeared onto glass slides, stained to highlight cellular structures, and scanned to create digital images. Specialized software analyses these images [46] to extract 30 different features, which quantify various morphological characteristics of the cell nuclei, such as size, shape, and texture. These features are used to create a predictive model for breast cancer diagnosis, with the target variable being the diagnosis categorized as malignant (M) or benign (B).

The chosen datasets include both classification and regression tasks and span a wide range in terms of number of participants, number of predictive features, achievable predictive effect size, and data homogeneity (Supplementary Figs 1–6). Our analyses aimed to contrast the proposed adaptive splitting method with the application of fixed training and validation sample sizes, specifically using 50%, 60%, or 90% of the total sample size for discovery and the rest for external validation. We simulated various “sample size budgets” (total sample sizes, ${n_{\textit{total}}}$) with random sampling without replacement. For a given total sample size, we simulated the prospective data acquisition procedure by incrementing ${n_{act}}$; starting with 10% of the total sample size and going up with increments of 5%. In each step, the stopping rule was evaluated with AdaptiveSplit, fitting a Ridge model (for regression tasks; HCP and IXI datasets) or a L2-regularized logistic regression (for classification tasks; ABIDE and BCW datasets). Model fit always consisted of a cross-validated fine-tuning of the $\alpha $ regularization parameter ($\alpha \in \{ {0.1,\,\,1,\,\,10} \}$), resulting in a nested cv estimate of prediction performance and validation power. Robust estimates (and confidence intervals) were obtained with bootstrapping, as described in Algorithm 1 and Algorithm 2. This procedure was iterated until the stopping rule returned True. The corresponding sample size was then considered the final discovery sample. With all four splitting approaches (adaptive, Pareto, half-split, 90%:10% split), we trained the previously described Ridge or regularized logistic regression model on the discovery sample and obtained predictions for the sample left out of the external validation. This whole procedure was repeated 100 times for each simulated sample size budget in each dataset to estimate the confidence intervals for the model’s performance in the external validation and its statistical significance. In all analyses, the adaptive splitting procedure is performed with a target power of ${v_{pow}} = 0.8$, and with $alpha\,\, = \,\,0.05$, ${t_{\textit{tmin}}} = {n_{\textit{total}}}/3$, ${v_{min}} = 12$, and ${s_{min}} = 0$. *P*-values were calculated using a permutation test with 5,000 permutations.

## Results

The results of our empirical analyses of four large, openly available datasets confirmed that the proposed adaptive splitting approach can successfully identify the optimal time to stop acquiring data for training and maintain a good compromise between maximizing both predictive performance and external validation power with any sample size budget.

In all four samples, the applied models yielded a statistically significant predictive performance at much lower sample sizes than the total size of the dataset, i.e., all datasets were well powered for the analysis. Thus, when reporting our results, we focused on the most realistic scenarios and omitted sample size budgets that were powered too low (neither of the splitting strategies leads to significant model performance) or too high (prediction performance plateaus with all splitting strategies) for any meaningful comparison between splitting strategies. After being trained on the full sample size with cross-validation, the models performed as follows: functional brain connectivity from the HCP dataset explained 13% of the variance in cognitive abilities; structural MRI data (gray matter probability maps) in the IXI dataset explained 48% in age; classification accuracy was 65.5% for autism diagnosis (functional brain connectivity) in the ABIDE dataset and 92% for breast cancer diagnosis in the BCW dataset.

The datasets varied not only in the achievable predictive performance but also in the shape of the learning curve, with different sample sizes and thus, they provided a good opportunity to evaluate the performance of our stopping rule in various circumstances (Supplementary Figs 1–6).

We found that adaptively splitting the data provided external validation performances that were comparable to the commonly used Pareto split (80%:20%) in most cases (Fig. 3, left column). From the fixed splitting approaches, the half-split assigns the least samples from the total sample size budget to the training phase (50%). Thus, the resulting model is trained on fewer data than with other strategies, typically resulting in a smaller ${l_{act}}$. Although this lower effect size should in general result in lower statistical power during the external validation phase, the half-split approach can counterbalance this with the larger sample size remaining for external validation. Our analysis shows that this happens in almost all the cases, hinting that in research scenarios where the expected predictive performance is low, researchers should either use the proposed adaptive splitting procedure, or aim for a relatively large pre-fixed external validation sample. In contrast, 90%:10% tended to display only slightly higher performances than the Pareto and the adaptive splitting techniques, in most cases. This small achievement came with a big cost in terms of the statistical power in the external validation sample, where the 90%:10% split very often gave inconclusive results ($P \ge 0.05$) (Fig. 3, right column), especially with low sample size budgets. Although to a lesser degree, the Pareto split also frequently failed to yield a conclusive external validation with small total sample sizes. In addition to the Pareto, half-split, and 90%:10% splitting strategies, we also evaluated alternative split ratios (75%:25% and 70%:30%), which are commonly used in the literature. The 75%:25% split demonstrated performance comparable to the Pareto and adaptive splitting techniques, although, similarly to Pareto, it struggled to achieve statistical significance at smaller sample sizes. In contrast, the 70%:30% split exhibited good statistical significance at the cost of lower overall performance, comparable to the trend observed with the half-split strategy (Supplementary Fig. 13). Adaptive splitting (as well as half-splitting) provided sufficient statistical power for the external validation in most cases. This was achieved by applying different strategies in different scenarios. In case of low total sample sizes, it retained a larger proportion of the sample for the external validation phase in order to achieve sufficient power, using up to 79% of the data for external validation. On the other hand, if the total sample size budget allowed it, adaptive splitting let the predictive model benefit from larger training samples, retaining 8% or less of the data for external validation in such cases.

Additionally, we report the performance of the models during the discovery phase, as illustrated in Supplementary Figs 8–12. Supplementary Fig. 12 extends the findings presented in Fig. 3 by addressing the discovery scores for each dataset and splitting strategy. Visual inspection of these scores reveals a high degree of consistency with the external validation scores, with only minor, negligible improvements observed in the latter. Supplementary Figs 8–11 facilitate a direct comparison between discovery and external validation performance by depicting the relationship between discovery scores, external validation scores, and the sample size at the chosen stopping point for each dataset and each splitting strategy. Color coding within these plots highlights the consistency of scores, which appears to be higher for bigger discovery sample sizes. A summary of all the reported scores is provided in Table 1.

**Table 1:** Performance results of the AdaptiveSplit algorithm for each dataset across the different sample sizes (${n_{act}}$). The fraction of discovery samples and external validation samples is shown for each split (Adaptive splits). For each ${n_{act}}$, the relative accuracy (for classification tasks) or negative mean absolute error (for regression tasks) is reported, along with statistical significance (*P*-value), providing a comprehensive overview of the algorithm's performance across different datasets and sample sizes.

Classification	BCW					ABIDE				
Sample sizes	49	65	86	113	150	400	442	489	542	599
Adaptive splits (discovery—external validation)	21–79	33–67	48–52	67–33	92–08	41–59	49–51	59–41	71–29	82–18
Discovery scores	0.888	0.921	0.933	0.938	0.944	0.614	0.624	0.633	0.640	0.644
External validation scores	0.896	0.927	0.935	0.941	0.944	0.626	0.634	0.634	0.643	0.655
Statistical significance	0.036	0.032	0.033	0.024	0.041	0.018	0.014	0.023	0.027	0.017
**Regression**	**HCP**					**IXI**				
Sample sizes	242	272	305	343	384	49	65	86	113	150
Adaptive splits (discovery—external validation)	44–56	53–47	64–36	76–24	89–11	21–79	25–75	40–60	61–39	89–11
Discovery scores	−15.35	−15.20	−15.10	−15.02	−14.89	−12.01	−11.93	−11.72	−11.54	−11.16
External validation scores	−15.24	−15.13	−15.09	−14.82	−14.82	−11.95	−12.08	−11.40	−11.12	−10.74
Statistical significance	0.012	0.016	0.019	0.033	0.029	0.070	0.031	0.014	0.021	0.041

Focusing only on cases with a successful, conclusive external validation, the proposed adaptive splitting strategy provided an external validation performance comparable to the alternative fixed splitting strategies in all cases where the external validation was conclusive (statistically significant). Furthermore, in contrast to the investigated fixed splitting strategies, the proposed splitting strategy yields solid guarantees for the success of the external validation phase, independent of the sample size budget.

## Discussion

Here we have proposed “registered models,” a novel design for prospective predictive modeling studies that allows flexible model discovery and trustworthy prospective external validation by fixing and publicly depositing the model after the discovery phase. Furthermore, capitalizing on the flexibility during model discovery with the registered model design, we have proposed a stopping rule for adaptively splitting the sample size budget into discovery and external validation phases. These approaches together provide a robust and flexible framework for predictive modeling studies and address several common issues in the field, including overfitting, effect size inflation as well as the lack of reliability and reproducibility.

Registered models provide a clear and transparent separation between the discovery and external validation phases, which is essential for ensuring the independence of the external validation data. Thereby, they provide a straightforward solution to several of the widely discussed issues and pitfalls of predictive model development [2, 6–8, 16]. With registered models, external validation estimates are guaranteed to be free of information leakage [9] and provide an unbiased estimate of the model’s predictive performance.

With registered models, the question of how the total sample size budget should be distributed between the discovery and external validation phase remains of central importance for the optimal use of available resources (scanning time, budget, limitations in participant recruitment) [2, 15–19, 47] (Supplementary Table 1). Optimal sample sizes are often challenging to determine prior to the study. The proposed adaptive splitting procedure promises to provide a solution in such cases by allowing the sample size to be adjusted during the data acquisition process, based on the observed performance of the model trained on the already available data. We performed a thorough evaluation of the proposed adaptive splitting procedure on data from more than 3,000 participants from four publicly available datasets. We found that the proposed adaptive splitting approach can successfully identify the optimal time to stop acquiring data for training and maintain a good compromise between maximizing both predictive performance and external validation power with any “sample size budget.” When contrasting splitting approaches based on fixed validation size with the proposed adaptive splitting technique, using the latter was always the preferable strategy to maximize power and statistical significance during external validation. The benefit of adaptively splitting the data acquisition for training and validation provides the largest benefit in lower sample size regimes. With larger total sample size budgets, the fixed Pareto split (80%:20%) also provided good results, giving similar external validation performances to adaptive splitting, without having to repeatedly retrain the model during data acquisition. Thus, for moderate-to-large sample sizes and well-powered models, the Pareto split might be a good alternative to the adaptive splitting approach, especially if the computational resources for retraining the model are limited.

Of note, the presented implementation of adaptive data splitting aims to maximize the discovery sample (and minimize the external validation sample) to achieve the highest possible performance together with a conclusive (statistically significant) external validation. However, the resulting external performance estimates will still be subject of sampling variance. If the aim is to provide more reliable estimates of the predictive effect size in the external validation, the power-rule in the proposed approach can be modified so that it stops the discovery phase when a desired confidence interval width for the external effect size estimate is reached.

The proposed adaptive splitting design can advance the development of predictive models in several ways. Firstly, it provides a simple way to perform both model discovery and initial external validation in a single study. Furthermore, it promotes the public deposition (registration) of models at an early stage of the study, enhancing transparency, reliability, and replicability. Finally, it provides a flexible approach to data splitting, which can be adjusted according to the specific needs of the study.

In conclusion, registered models provide a simple approach to guarantee the independence of model discovery and external validation, and for the development and initial evaluation of registered models with unknown power, the introduced adaptive splitting procedure provides a robust and flexible approach to determine the optimal ratio of data to be used for model discovery and external validation. Together, registered models and the adaptive splitting procedure address several common issues in the field, including overfitting and cross-validation failure, and boost the reliability and reproducibility.

## Availability of source code and requirements

Project name: AdaptiveSplit

Project home page: https://github.com/pni-lab/adaptivesplit

Operating system(s): Platform independent

Programming language: Python

Other requirements: Python 3.9 or higher

License: GNU General public licence, version 3, 29 June 2007 (GPL-3.0)

RRID:SCR_025888

bio.tools: bio.tools:adaptivesplit

Archival copies of the code repositories are available via Software Heritage [49].

## Supplementary Material

giaf036_Supplemental_File

giaf036_Authors_Response_To_Reviewer_Comments_Original_Submission

giaf036_Authors_Response_To_Reviewer_Comments_Revision_1

giaf036_Authors_Response_To_Reviewer_Comments_Revision_2

giaf036_GIGA-D-24-00187_Original_Submission

giaf036_GIGA-D-24-00187_Revision_1

giaf036_GIGA-D-24-00187_Revision_2

giaf036_GIGA-D-24-00187_Revision_3

giaf036_Reviewer_1_Report_Original_Submission Qingyu Zhao -- 7/30/2024

giaf036_Reviewer_1_Report_Revision_1 Qingyu Zhao -- 12/10/2024

giaf036_Reviewer_2_Report_Original_Submission Lisa Crossman -- 8/29/2024

giaf036_Reviewer_2_Report_Revision_1 Lisa Crossman -- 12/2/2024

## Data Availability

Empirical analysis was based on data provided by the following sources: (1) the Human Connectome Project (WU-Minn Consortium, principal investigators: D. Van Essen and K. Ugurbil; 1U54MH091657), funded by the sixteen National Institutes of Health (NIH) institutes and centers that support the NIH Blueprint for Neuroscience Research; (2) the ABIDE consortium [31], (3) Imperial College London (IXI, principal investigator: D. L. Hill, other investigators: S. C. R. Williams, S. M. Smith, and D. Hawkes; GR/S21533/02); and (4) the University of Wisconsin [34]. Raw and preprocessed data used in the present study are publicly available for download in their respective repositories: ABIDE raw data [31] available via the 1000 Functional Connectomes Project [51]. ABIDE preprocessed dataset [35] available via osf.io [52] HCP1200 raw data [32] available via ConnectomeDB [53] HCP1200 preprocessed data [41] available via Human Connectome Project [54] BCW preprocessed dataset [34] available at Kaggle [55]. IXI raw data [33] available via Biomedical Image Analysis Group—IXI Dataset [56] IXI preprocessed dataset [44] available via Zenodo [57]. The Python implementation of the AdaptiveSplit package is publicly available on GitHub [29] (https://github.com/pni-lab/adaptivesplit). Additionally, the Python scripts and data used for the analyses presented in this manuscript can be accessed in the GitHub repository [50], with archival copies of the code available in Software Heritage [58]. Dome-ML (Data, Optimization, Model and Evaluation in Machine Learning) annotations are available via the DOME registry under accession 0p6q20kd4b [48].

## Additional Files

**Supplemental Figure 1:** Heatmap showing the predictive performance of the model trained on the BCW dataset to predict diagnosis.

**Supplemental Figure 2:** Learning and power curves of the model trained on the BCW dataset, generated by the AdaptiveSplit package.

**Supplemental Figure 3:** Scatterplot showing the predictive performance of the model trained on the IXI dataset to predict age.

**Supplemental Figure 4:** Learning and power curve of the model trained on the IXI dataset to predict age.

**Supplemental Figure 5:** Scatterplot showing the predictive performance of the model trained on the HCP dataset to predict fluid intelligence.

**Supplemental Figure 6:** Learning and power curve of the model trained on the HCP dataset to predict fluid intelligence.

**Supplemental Figure 7:** Learning curves for three different models trained on the BCW dataset full sample size to show how the performance rule works.

**Supplemental Figure 8:** Scatterplots showing the relationship between discovery scores, external validation scores, and sample sizes for the BCW dataset.

**Supplemental Figure 9:** Scatterplots showing the relationship between discovery scores, external validation scores, and sample sizes for the ABIDE dataset.

**Supplemental Figure 10:** Scatterplots showing the relationship between discovery scores, external validation scores, and sample sizes for the HCP dataset.

**Supplemental Figure 11:** Scatterplots showing the relationship between discovery scores, external validation scores, and sample sizes for the IXI dataset.

**Supplemental Figure 12:** Line plots that extend Fig. 3 from the main text and show the discovery scores for all the four datasets.

**Supplemental Figure 13:** Line plots showing discovery scores, external validation scores, and *P*-values for additional data splits.

**Supplementary Table 1:** References regarding the topic of brain-behavior associations and their reproducibility.
