# Supplementary material for: External validation of machine learning models—registered models and adaptive sample splitting
Source: Gigascience. 2025 May 14;14:giaf036. doi: 10.1093/gigascience/giaf036 (PMC12077397; doi:10.1093/gigascience/giaf036)
Supplement: giaf036_Supplemental_File [file giaf036_supplemental_file.pdf]

# Supplementary Material

*Giuseppe Gallitto, Robert Englert, Balint Kincses, Raviteja Kotikalapudi, Jialin Li, Kevin Hoffschlag, Ulrike Bingel, Tamas Spisak*

## Supplementary Figures

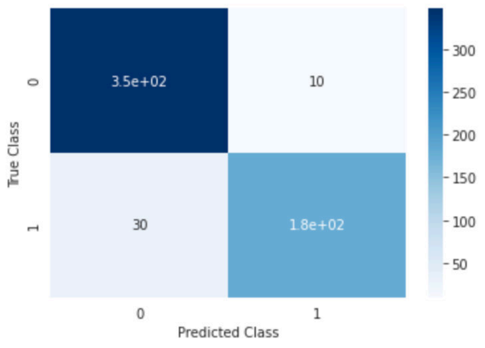


**Figure 1:** Predictive performance (confusion matrix) of the model trained on the BCW dataset to predict diagnosis. The model was trained on the whole dataset with nested cross-validation.

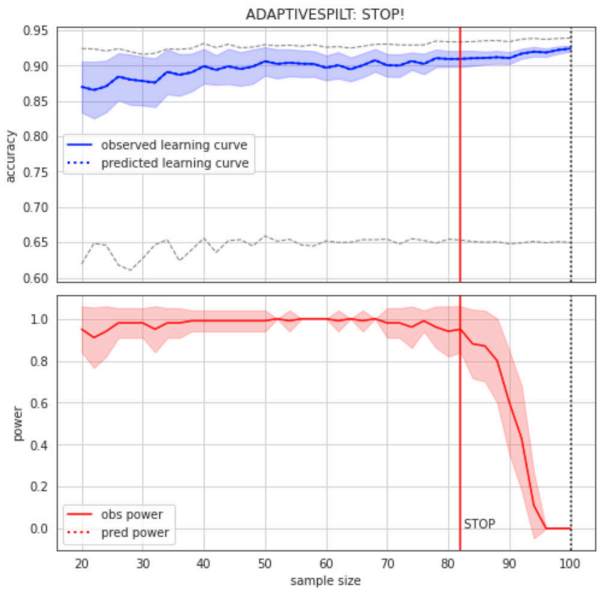


**Figure 2:** Learning curve (top) and power curve (bottom) of the model trained on the BCW dataset to predict diagnosis. The maximum sample size (i.e. the whole dataset) was considered as the "sample size budget". X-axis: $n_{act}$; y-axis (learning curve): Accuracy as a measure of predictive performance; y-axis (power curve): statistical power of the remaining sample to confirm the model's validity.

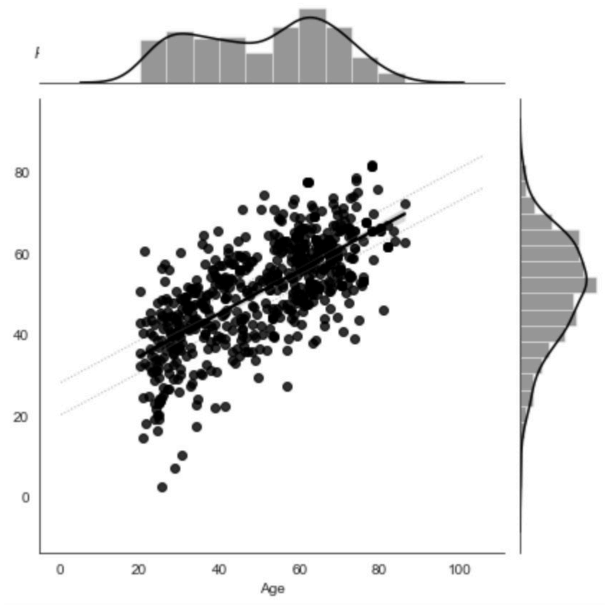


**Figure 3:** Predictive performance of the model trained on gray matter probability images from the IXI dataset to predict age. The model was trained on the whole dataset with nested cross-validation. X-axis: true age, y-axis: predicted age.

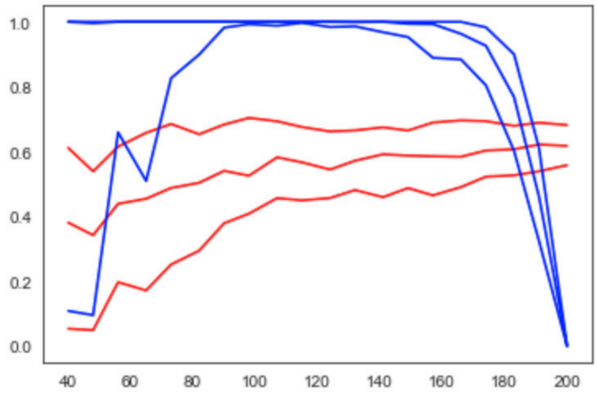


**Figure 4:** Learning curve (red) and power curve (blue) of the model trained on gray matter probability images from the IXI dataset to predict age. The maximum sample size (i.e. the whole dataset) was considered as the "sample size budget". X-axis: $n_{act}$; y-axis (learning curve): Pearson's correlation as a measure of predictive performance; y-axis (power curve): statistical power of the remaining sample to confirm the model's validity.

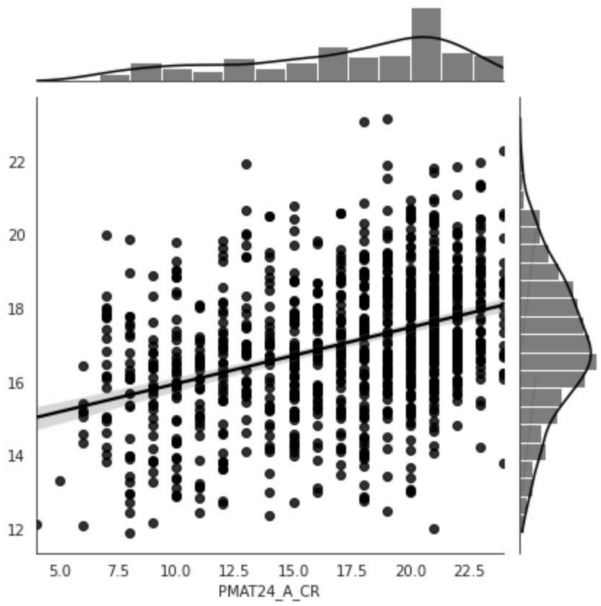


**Figure 5:** Predictive performance of the model trained on resting state functional connectivity data from the HCP dataset to predict fluid intelligence (PMAT24_A_CR). The model was trained on the whole dataset with nested cross-validation. X-axis: true age, y-axis: predicted age.

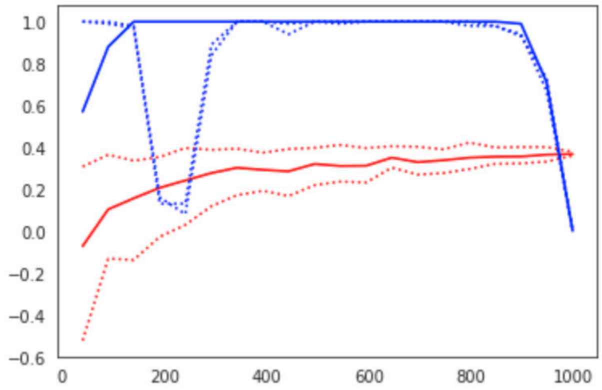


**Figure 6:** Learning curve (red) and power curve (blue) of the model trained on resting state functional connectivity data from the HCP dataset to predict fluid intelligence (PMAT24_A_CR). The maximum sample size (i.e. the whole dataset) was considered as the "sample size budget". X-axis: $n_{act}$; y-axis (learning curve): Pearson's correlation as a measure of predictive performance; y-axis (power curve): statistical power of the remaining sample to confirm the model's validity.

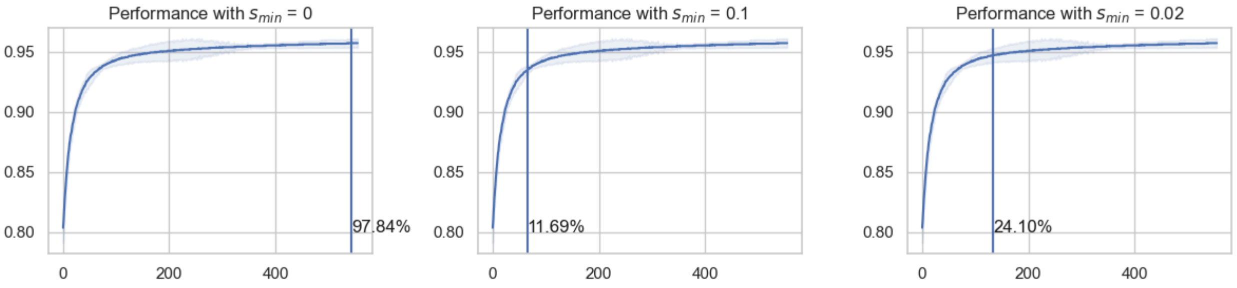


**Figure 7:** Learning curves for models trained on the BCW dataset (569 samples) to predict diagnoses. The models are evaluated using the adaptive-split's performance rule, which assesses whether the learning curve is plateauing. At each $n_{act}$, we calculate the tangent line to the curve. If the slope of the tangent line is less than or equal to the minimum relative score ($s_{min}$), the algorithm stops, signaling that the necessary size for the discovery set has been reached and data can be split. If $s_{min}$ is set to 0, the performance rule is not active.

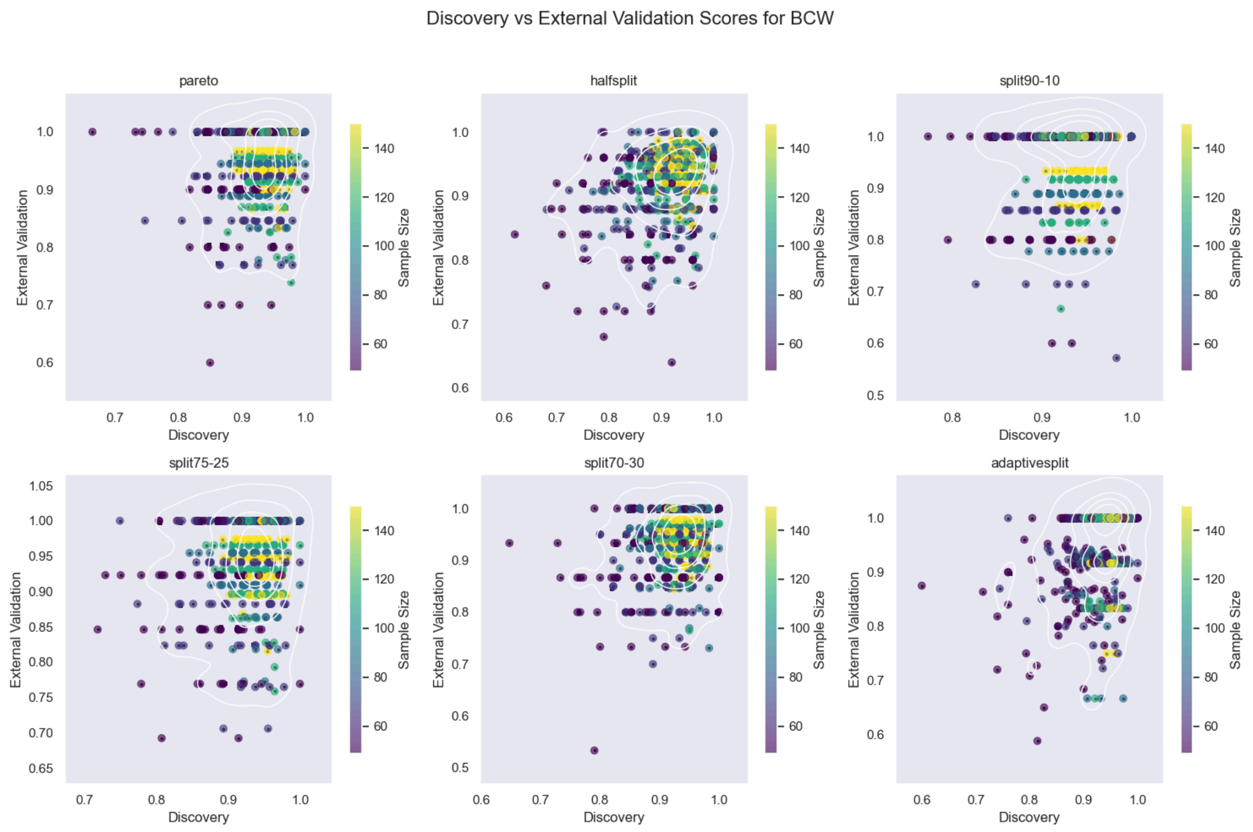


**Figure 8:** Plots showing the relationship between discovery scores, external validation scores, and sample size for the BCW dataset. These plots illustrate how model performance on the discovery set compares with external validation as the sample size increases.

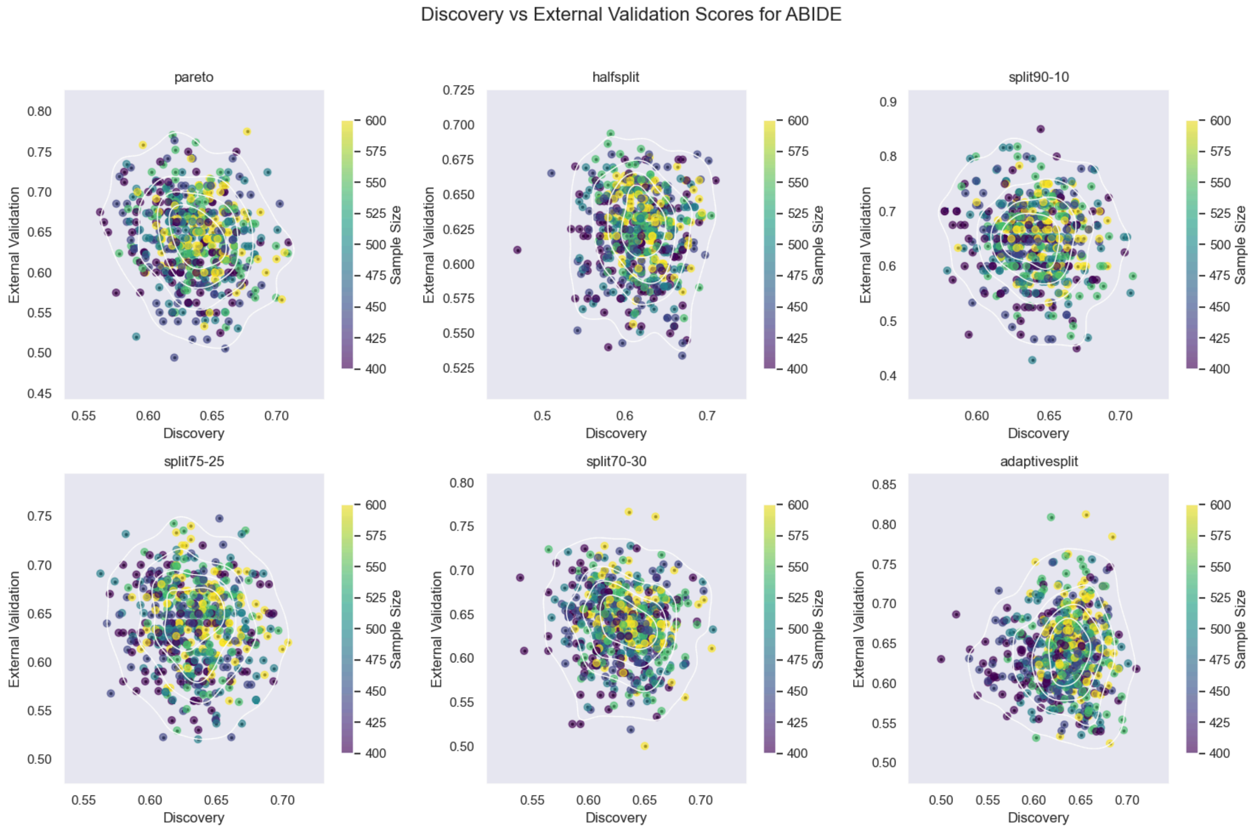


**Figure 9:** Plots showing the relationship between discovery scores, external validation scores, and sample size for the ABIDE dataset. These plots illustrate how model performance on the discovery set compares with external validation as the sample size increases.

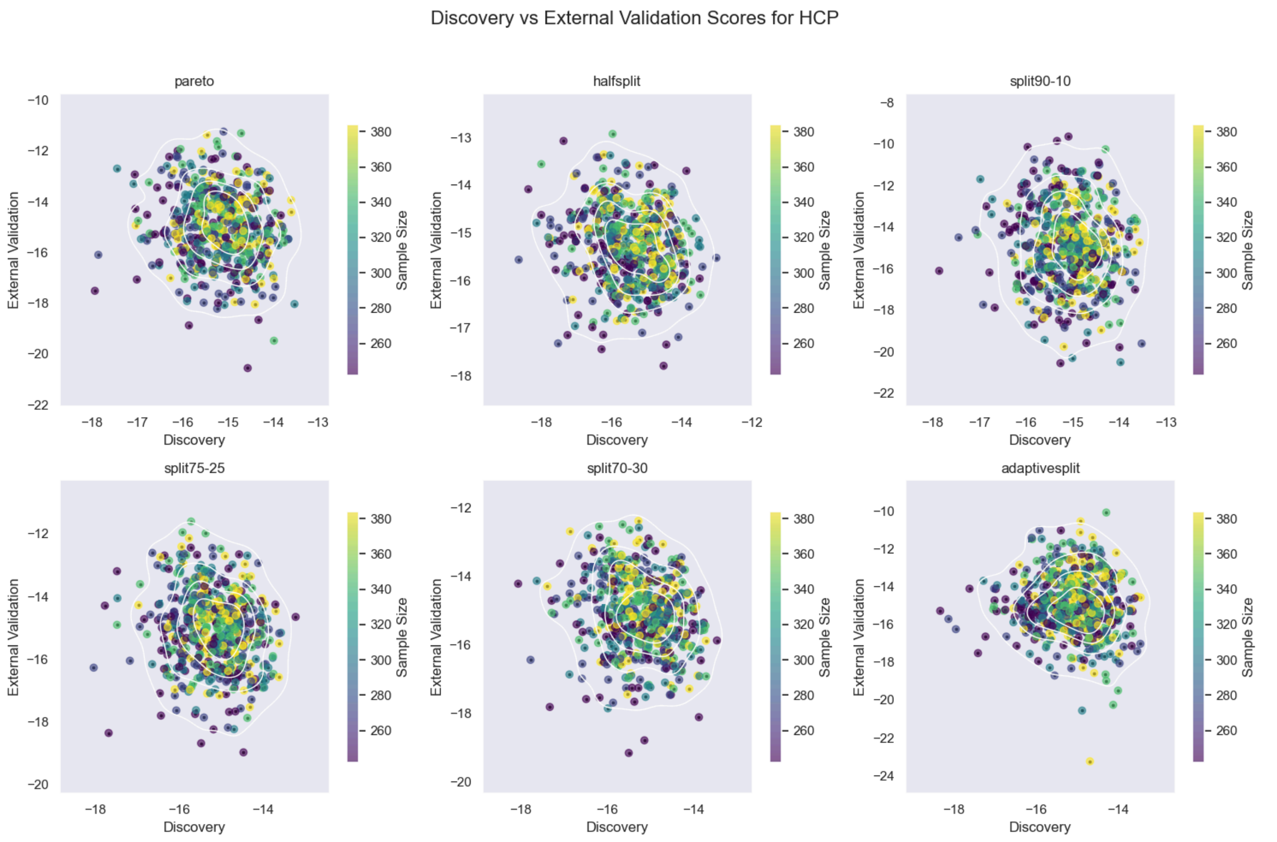


**Figure 10:** Plots showing the relationship between discovery scores, external validation scores, and sample size for the HCP dataset. These plots illustrate how model performance on the discovery set compares with external validation as the sample size increases.

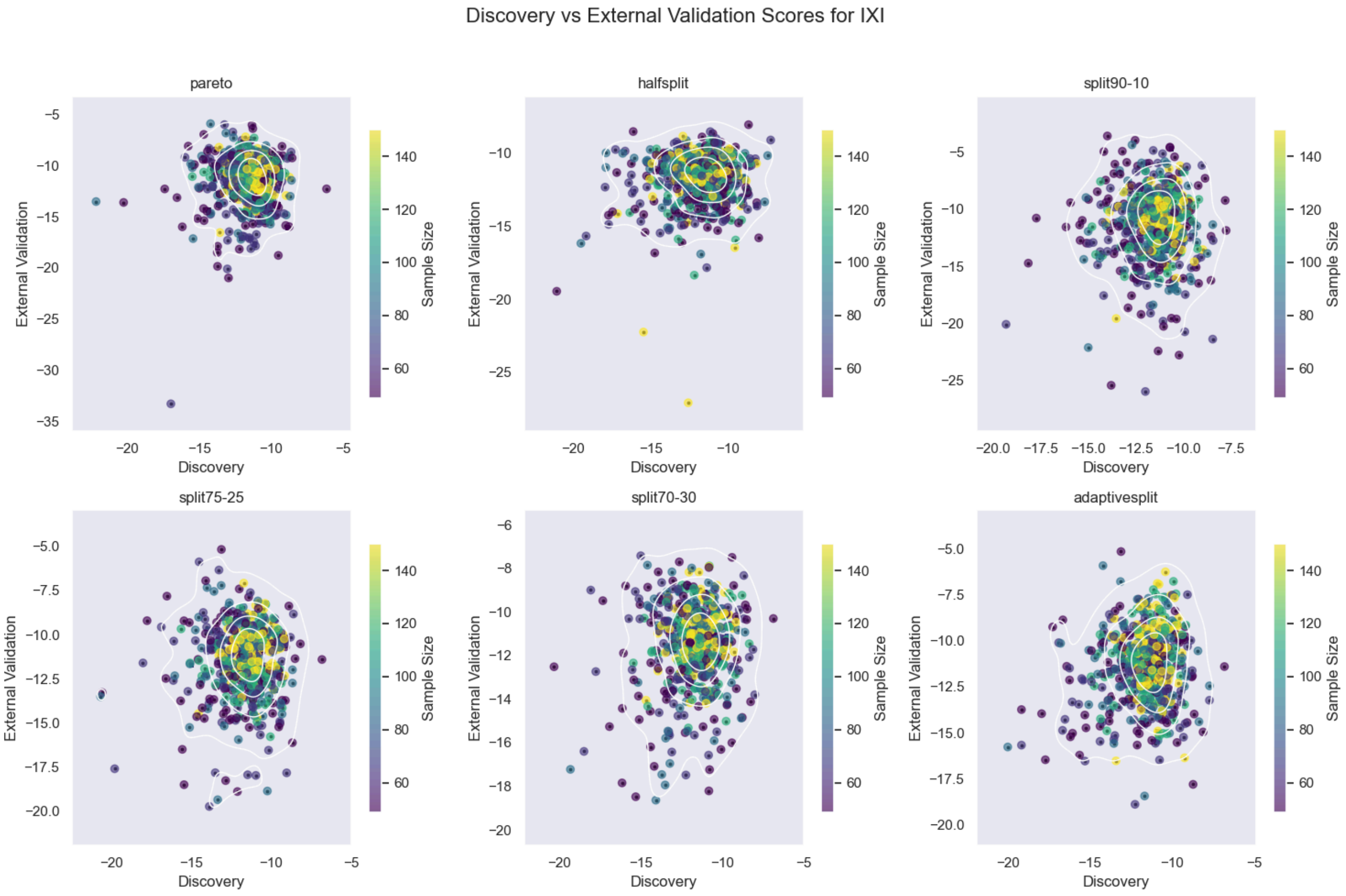


**Figure 11:** Plots showing the relationship between discovery scores, external validation scores, and sample size for the IXI dataset. These plots illustrate how model performance on the discovery set compares with external validation as the sample size increases.

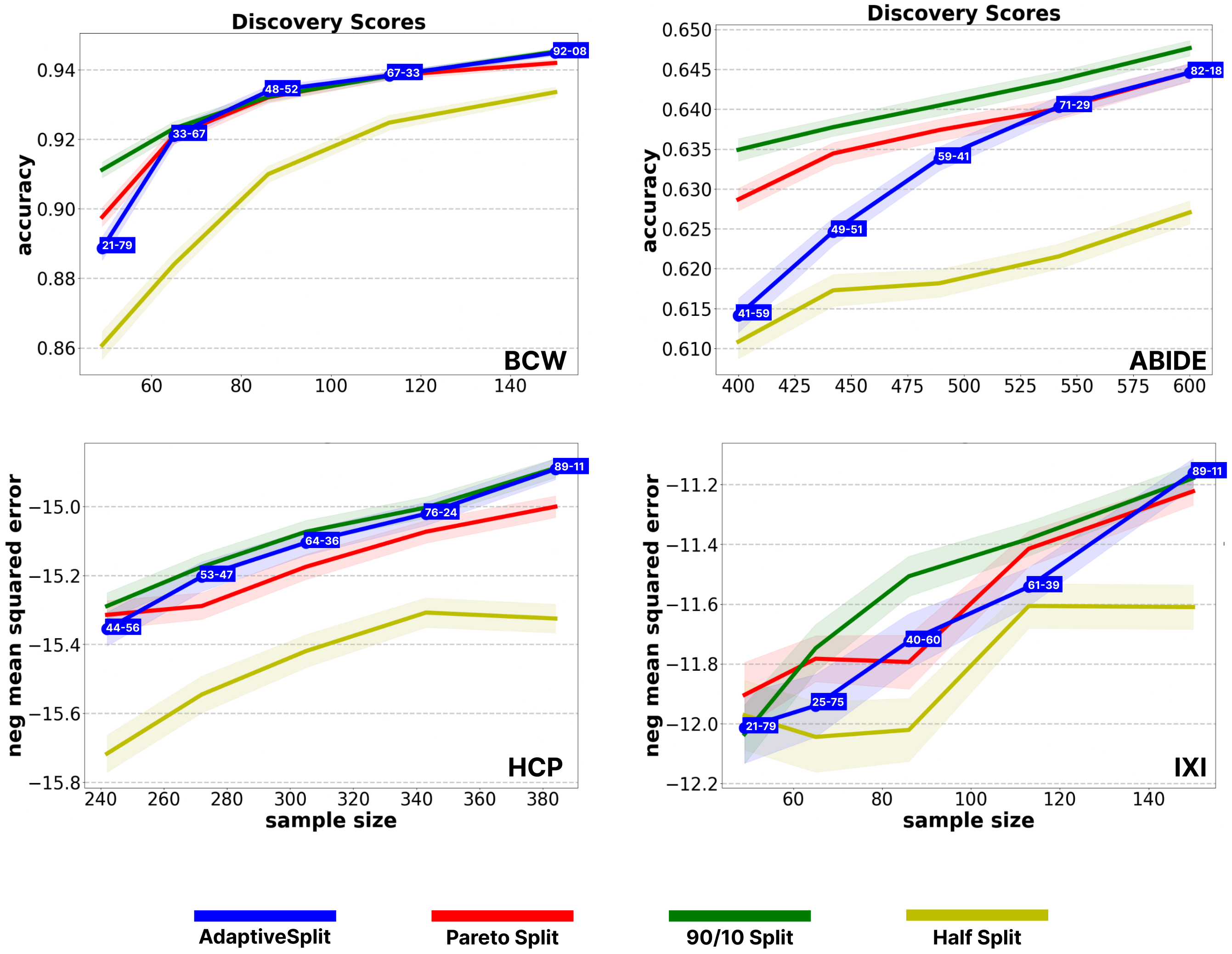


**Figure 12:** Extension of Fig. 3 from the main text, illustrating the splits and performance during the discovery phase for all four datasets.

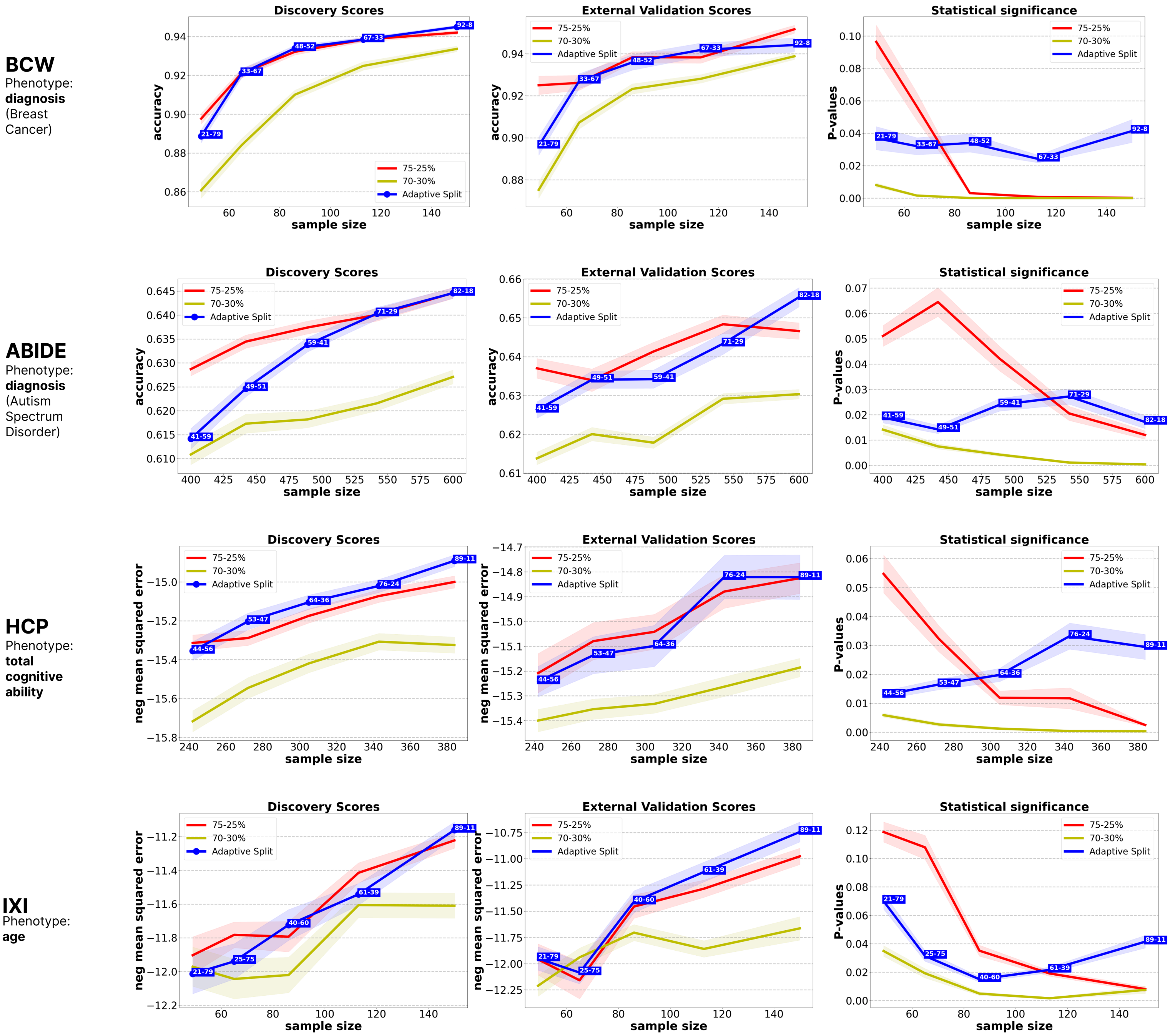


**Figure 13:** Figure illustrating the discovery scores, external validation scores, and p-values for additional data splits (75-25% and 70-30%) in comparison to the adaptive-split method. The 75-25% split shows a performance comparable to pareto and adaptivesplit but comes with lower statistical significance at lower sample sizes, while the 70-30% split is always robust but at the price of lower performance.

# Supplementary Tables

## Supplementary Table 1

Manuscripts, commentaries, and editorials on the topic of brain-behavior associations and their reproducibility, related to Marek *et al.*, 2022. See the up-to-date list here: https://spisakt.github.io/BWAS_comment/

| Authors | Title | Where |
|---|---|---|
| Nature editorial | Cognitive neuroscience at the crossroads | Nature |
| Spisak et al. | Multivariate BWAS can be replicable with moderate sample sizes | Nature |
| [Nat. Neurosci. editorial ] | Revisiting doubt in neuroimaging research | Nat. Neurosci. |
| Monica D. Rosenberg and Emily S. Finn | How to establish robust brain–behavior relationships without thousands of individuals | Nat. Neurosci. |
| Bandettini P et al. | The challenge of BWAS: Unknown Unknowns in Feature Space and Variance | Med |
| Gratton C. et al. | Brain-behavior correlations: Two paths toward reliability | Neuron |
| Cecchetti L. and Handjaras G. | Reproducible brain-wide association studies do not necessarily require thousands of individuals | psyArXiv |
| Winkler A. et al. | We need better phenotypes | brainder.org |
| DeYoung C. et al. | Reproducible between-person brain-behavior associations do not always require thousands of individuals | psyArXiv |
| Gell M et al. | The Burden of Reliability: How Measurement Noise Limits Brain-Behaviour Predictions | bioRxiv |
| Tiego J. et al. | Precision behavioral phenotyping as a strategy for uncovering the biological correlates of psychopathology | OSF |
| Chakravarty MM. | Precision behavioral phenotyping as a strategy for uncovering the biological correlates of psychopathology | Nature Mental Health |
| White T. | Behavioral phenotypes, stochastic processes, entropy, evolution, and individual variability: Toward a unified field theory for neurodevelopment and psychopathology | OHBM Aperture Neuro |

| | | |
|---|---|---|
| Bandettini P. | Lost in transformation: fMRI power is diminished by unknown variability in methods and people | OHBM Aperture Neuro |
| Thirion B. | On the statistics of brain/behavior associations | OHBM Aperture Neuro |
| Tiego J., Fornito A. | Putting behaviour back into brain–behaviour correlation analyses | OHBM Aperture Neuro |
| Lucina QU. | Brain-behavior associations depend heavily on user-defined criteria | OHBM Aperture Neuro |
| Valk SL., Hettner MD. | Commentary on 'Reproducible brain-wide association studies require thousands of individuals' | OHBM Aperture Neuro |
| Kong XZ., et al. | Scanning reproducible brain-wide associations: sample size is all you need? | Psychoradiology |
| J. Goltermann, et al. | Cross-validation for the estimation of effect size generalizability in mass-univariate brain-wide association studies | BioRxiv |
| Kang K., et al. | Study design features that improve effect sizes in cross-sectional and longitudinal brain-wide association studies | BioRxiv |
| Makowski C., et al. | Reports of the death of brain-behavior associations have been greatly exaggerated | BioRxiv |
| J. Wu et al. | The challenges and prospects of brain-based prediction of behaviour | Nat. Human Behaviour |

## References

(2022). *Nature Neuroscience*, *25*(7), 833–834. 10.1038/s41593-022-01125-2

Kang, K., Seidlitz, J., Bethlehem, R. A. I., Xiong, J., Jones, M. T., Mehta, K., Keller, A. S., Tao, R., Randolph, A., Larsen, B., Tervo-Clemmens, B., Feczko, E., Miranda Dominguez, O., Nelson, S., Schildcrout, J., Fair, D., Satterthwaite, T. D., Alexander-Bloch, A., & Vandekar, S. (2023). *Study design features that improve effect sizes in cross-sectional and longitudinal brain-wide association studies*. 10.1101/2023.05.29.542742

Makowski, C., Brown, T. T., Zhao, W., Hagler, D. J., Parekh, P., Garavan, H., Nichols, T. E., Jernigan, T. L., & Dale, A. M. (2023). *Leveraging the Adolescent Brain Cognitive Development Study to improve behavioral prediction from neuroimaging in smaller replication samples*. 10.1101/2023.06.16.545340

Marek, S., Tervo-Clemmens, B., Calabro, F. J., Montez, D. F., Kay, B. P., Hatoum, A. S., Donohue, M. R., Foran, W., Miller, R. L., Hendrickson, T. J., Malone, S. M., Kandala, S., Feczko, E., Miranda-Dominguez, O., Graham, A. M., Earl, E. A., Perrone, A. J., Cordova, M., Doyle, O., … Dosenbach, N. U. F. (2022). Reproducible brain-wide association studies require thousands of individuals. *Nature*, *603*(7902), 654–660. 10.1038/s41586-022-04492-9

Tiego, J., & Fornito, A. (2023). Putting behaviour back into brain–behaviour correlation analyses. *Aperture Neuro*. 10.52294/2f9c5854-d10b-44ab-93fa-d485ef5b24f1

Wu, J., Li, J., Eickhoff, S. B., Scheinost, D., & Genon, S. (2023). The challenges and prospects of brain-based prediction of behaviour. *Nature Human Behaviour*, *7*(8), 1255–1264. 10.1038/s41562-023-01670-1
