# Supplementary material for: External validation of machine learning models—registered models and adaptive sample splitting
Source: Gigascience. 2025 May 14;14:giaf036. doi: 10.1093/gigascience/giaf036 (PMC12077397; doi:10.1093/gigascience/giaf036)
Supplement: giaf036_GIGA-D-24-00187_Revision_1 [file giaf036_giga-d-24-00187_revision_1.pdf]

GigaScience

External validation of machine learning models - registered models and adaptive sample splitting

--Manuscript Draft--

**Manuscript Number:**	GIGA-D-24-00187R1
**Full Title:**	External validation of machine learning models - registered models and adaptive sample splitting
**Article Type:**	Research
**Funding Information:**	Deutsche Forschungsgemeinschaft (422744262) — Not applicable
**Abstract:**	Multivariate predictive models play a crucial role in enhancing our understanding of complex biological systems and in developing innovative, replicable tools for translational medical research. However, the complexity of machine learning methods and extensive data pre-processing and feature engineering pipelines can lead to overfitting and poor generalizability. An unbiased evaluation of predictive models necessitates external validation, which involves testing the finalized model on independent data. Despite its importance, external validation is often neglected in practice due to the associated costs. Here we propose that, for maximal credibility, model discovery and external validation should be separated by the public disclosure (e.g. pre-registration) of feature processing steps and model weights. Furthermore, we introduce a novel approach to optimize the trade-off between efforts spent on model discovery and external validation in such studies. We show on data involving more than 3000 participants from four different datasets that, for any “sample size budget”, the proposed adaptive splitting approach can successfully identify the optimal time to stop model discovery so that predictive performance is maximized without risking a low powered, and thus inconclusive, external validation. The proposed design and splitting approach (implemented in the Python package “AdaptiveSplit”) may contribute to addressing issues of replicability, effect size inflation and generalizability in predictive modeling studies.
**Corresponding Author:**	Giuseppe Gallitto, MSc<br>University Medicine Essen Ruhrlandklinik: Ruhrlandklinik<br>Essen, GERMANY
**Corresponding Author Secondary Information:**	
**Corresponding Author's Institution:**	University Medicine Essen Ruhrlandklinik: Ruhrlandklinik
**Corresponding Author's Secondary Institution:**	
**First Author:**	Giuseppe Gallitto
**First Author Secondary Information:**	
**Order of Authors:**	Giuseppe Gallitto
	Robert Englert
	Balint Kincses
	Raviteja Kotikalapudi
	Jialin Li
	Kevin Hoffschlag
	Ulrike Bingel
	Tamas Spisak
**Order of Authors Secondary Information:**	
**Response to Reviewers:**	Dear Editorial Team,

Powered by Editorial Manager® and ProduXion Manager® from Aries Systems Corporation

	Please find attached our response letter (Letter.docx), in which we address all the comments and concerns raised by both the reviewers and the editorial team. Additionally, we have included a revised version of the manuscript with tracked changes.<br><br>As outlined in our letter, we hope that our revisions have sufficiently addressed all the points raised, and that the manuscript is now suitable for publication in GigaScience. We remain happy to address any further questions or suggestions you or the reviewers may have.<br><br>Thank you for your consideration.<br><br>Yours sincerely,<br>Giuseppe Gallitto<br>On behalf of all authors
**Additional Information:**	
**Question**	**Response**
Are you submitting this manuscript to a special series or article collection?	No
**Experimental design and statistics**<br><br>Full details of the experimental design and statistical methods used should be given in the Methods section, as detailed in our Minimum Standards Reporting Checklist. Information essential to interpreting the data presented should be made available in the figure legends.<br><br>Have you included all the information requested in your manuscript?	Yes
**Resources**<br><br>A description of all resources used, including antibodies, cell lines, animals and software tools, with enough information to allow them to be uniquely identified, should be included in the Methods section. Authors are strongly encouraged to cite Research Resource Identifiers (RRIDs) for antibodies, model organisms and tools, where possible.<br><br>Have you included the information requested as detailed in our Minimum Standards Reporting Checklist?	Yes

Powered by Editorial Manager® and ProduXion Manager® from Aries Systems Corporation

**Availability of data and materials**<br><br>All datasets and code on which the conclusions of the paper rely must be either included in your submission or deposited in publicly available repositories (where available and ethically appropriate), referencing such data using a unique identifier in the references and in the "Availability of Data and Materials" section of your manuscript.<br><br>Have you have met the above requirement as detailed in our Minimum Standards Reporting Checklist?	Yes

Manuscript

Click here to access/download;Manuscript;adaptivesplit_manuscript.docx

External validation of machine learning models - registered models and adaptive sample splitting

Giuseppe Gallitto[1,2], Robert Englert[1,3], Balint Kincses[1,2], Raviteja Kotikalapudi[1,2], Jialin Li[1,2,4], Kevin Hoffschlag[1,2], Ulrike Bingel[1,2], Tamas Spisak[1,3]*

1 Center for Translational Neuro- and Behavioral Sciences (C-TNBS), University Medicine Essen, Germany

2 Department of Neurology, University Medicine Essen, Germany

3 Department of Diagnostic and Interventional Radiology and Neuroradiology, University Medicine Essen, Germany

4 Max Planck School of Cognition, Leipzig, Germany

** Corresponding author*

Abstract

Multivariate predictive models play a crucial role in enhancing our understanding of complex biological systems and in developing innovative, replicable tools for translational medical research. However, the complexity of machine learning methods and extensive data pre-processing and feature engineering pipelines can lead to overfitting and poor generalizability. An unbiased evaluation of predictive models necessitates external validation, which involves testing the finalized model on independent data. Despite its importance, external validation is often neglected in practice due to the associated costs. Here we propose that, for maximal credibility, model discovery and external validation should be separated by the public disclosure (e.g. pre-registration) of feature processing steps and model weights. Furthermore, we introduce a novel approach to optimize the trade-off between efforts spent on model discovery and external validation in such studies. We show on data involving more than 3000 participants from four different datasets that, for any "sample size budget", the proposed adaptive splitting approach can successfully identify the optimal time to stop model discovery so that predictive performance is maximized without risking a low powered, and thus inconclusive, external validation. The proposed design and splitting approach (implemented in the Python package "AdaptiveSplit") may contribute to addressing issues of replicability, effect size inflation and generalizability in predictive modeling studies.

Keywords: machine learning; predictive modelling; preregistration, external validation, adaptive splitting

Introduction

Multivariate predictive models integrate information across multiple variables to construct predictions of a specific outcome and hold promise for delivering more accurate estimates than traditional univariate methods (Woo *et al.*, 2017). For instance, in case of predicting individual behavioral and psychometric characteristics from brain data, such models can provide higher statistical power and better replicability, as compared to conventional mass-univariate analyses (Spisak *et al.*, 2023). Predictive models can utilize a variety of algorithms, ranging from simple linear regression-based models to complex deep neural networks. With increasing model complexity, the model will be more prone to overfit its training dataset, resulting in biased, overly optimistic in-sample estimates of predictive performance and often decreased generalizability to data not seen during model fit (Hosseini *et al.*, 2020). Internal validation approaches, like cross-validation (cv) provide means for an unbiased evaluation of predictive performance during model discovery by repeatedly holding out parts of the discovery dataset for testing purposes (Efron & Tibshirani, 1994; Poldrack *et al.*, 2020). However, internal validation approaches, in practice, still tend to yield overly optimistic performance estimates (Efron, 1983; Sui *et al.*, 2020; Varoquaux & Cheplygina, 2022). There are several reasons for this kind of effect size inflation. First, predictive modelling approaches typically display a high level of "analytical flexibility" and pose a large number of possible methodological choices in terms of feature pre-processing and model architecture, which emerge as uncontrolled (e.g. not cross-validated) "hyperparameters" during model discovery. Seemingly 'innocent' adjustments of such parameters can also lead to overfitting, if it happens outside the cv loop. The second reason for inflated internally validated performance estimates is 'leakage' of information from the test dataset to the training dataset (Kapoor & Narayanan, 2023). Information leakage has many faces. It can be a consequence of, for instance, feature standardization in a non cv-compliant way or, in medical imaging, the co-registration of brain data to a study-specific template. Therefore, it is often very hard to notice, especially in complex workflows. Another reason for overly optimistic internal validation results may be that even the highest quality discovery datasets can only yield an imperfect representation of the real world. Therefore, predictive models might capitalize on associations that are specific to the dataset at hand and simply fail to generalize "out-of-the-distribution", e.g. to different populations. Finally, some models might also be overly sensitive to unimportant characteristics of the training data, like subtle differences between batches of data acquisition or center-effects (Prosperi *et al.*, 2020; Spisak, 2022).

The obvious solution for these problems is *external validation*; that is, to evaluate the model's predictive performance on independent ('external') data that is guaranteed to be unseen during the whole model discovery procedure. There is a clear agreement in the community that external validation is critical for establishing machine learning model quality (Collins *et al.*, 2014; Ho *et al.*, 2020; Yu *et al.*, 2022; Spisak *et al.*, 2023; Poldrack *et al.*, 2020). However, the amount of data to be used for model discovery and external validation can have crucial implications on the predictive power, replicability and validity of predictive models and is, therefore, subject of intense discussion (Riley *et al.*, 2021; Marek *et al.*, 2022; Spisak *et al.*, 2023; Rosenberg & Finn, 2022; Thirion, 2023; Makowski *et al.*, 2023; Supplementary Table 1). Finding the optimal sample sizes is especially challenging for biomedical research, where this trade-off needs to weigh-in ethical and economic considerations. As a consequence, to date only around 10% of predictive modeling studies include an external validation of the model (Yang *et al.*, 2022). Those few studies performing true external validation often perform it on retrospective data (like Lee *et al.*, 2021 or Kincses *et al.*, 2024) or in separate, prospective studies (Spisak *et al.*, 2020; Kincses *et al.*, 2024). Both approaches can result in a suboptimal use of data and may slow down the dissemination process of new results.

In this manuscript we argue that maximal reliability and transparency during external validation can be achieved with prospective data acquisition preceded by "freezing" and publicly depositing (e.g. pre-registering) the whole feature processing workflow and all model weights. Furthermore, we present a novel adaptive design for predictive modeling studies with prospective data acquisition that optimizes the trade-off between efforts spent on model discovery and external validation. We evaluate the proposed approach on data involving more than 3000 participants from four different datasets to illustrate that for any "sample size budget", it can successfully identify the optimal time to stop model discovery, so that predictive performance is maximized without risking a low powered, and thus inconclusive, external validation.

Background

The anatomy of a prospective predictive modelling study

Let us consider the following scenario: a research group plans to involve a fixed number of participants in a study with the aim of constructing a predictive model, and at the same time, evaluate its external validity. How many participants should they allocate for model discovery, and how many for external validation, to get the highest performing model as well as conclusive validation results?

In most cases it is very hard to make an educated guess about the optimal split of the total sample size into discovery and external validation samples prior to data acquisition. A possible approach is to use simplistic rules-of-thumb. Splitting data with an 80-20% ratio (a.k.a Pareto-split, Lipovetsky, 2009) is probably the most common method, but a 90-10% or a 50-50% may also be plausible choices (Raykar & Saha, 2015). However, as illustrated on Figure 1, such prefixed sample sizes are likely sub-optimal in many cases and the optimal strategy is actually determined by the dependence of the model performance on training sample size, that is, the "learning curve". For instance, in case of a significant but generally low model performance (Figure 1A: flat learning curve) the model does not benefit a lot from adding more data to the discovery set but, on the other hand, it may require a larger external validation set for conclusive evaluation, due to the lower predictive effect size. This is visualized by the "power curve" on Figure 1, which shows the statistical power of external validation with the remaining samples as a function of sample size used for model discovery. The optimal strategy will be different, however, if the learning curve shows a persistent increase, without a strong saturation effect, meaning that predictive performance can be significantly enhanced by training the model on larger sample size (Figure 1B). In this case, the stronger predictive performance that can be achieved with larger training sample size, at the same time, allows a smaller external validation sample to be still conclusive. Finally, in some situations, model performance may rapidly get strong and reach a plateau at a relatively low sample size (Figure 1C). In such cases, the optimal strategy might be to stop early with the discovery phase and allocate resources for a more powerful external validation.

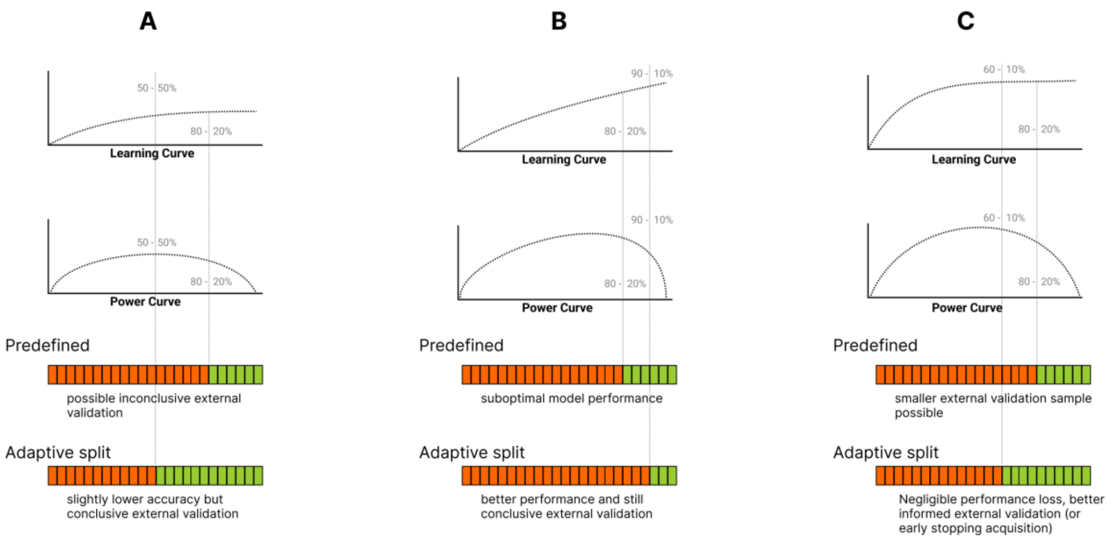

Figure 1: Examples of different optimal discovery and external validation sample sizes compared to a predefined 80-20% Pareto-split. (A) If the planned sample size and the model performance is low, the predefined external validation sample size might provide low statistical power to detect a significant model performance. **(B)** External validation of highly accurate models is well-powered; increasing the discovery sample size (against the external validation sample size) might result in a better performing final model. **(C)** Continuing training on the plateau of the learning curve will result in a negligible or biologically not relevant model performance improvement. In this case, a larger external validation sample (for more robust external performance estimates) or 'early stopping' of the data acquisition process might be desirable.

Transparent reporting of external validation: registered models

A key criterion for external validation is the independence of the external data from the data used during model discovery (Steyerberg & Harrell, 2016; Collins *et al.*, 2014; Spisak *et al.*, 2023). Regardless of the splitting strategy, an externally validated predictive modelling study must provide strong guarantees for this independence criterion. Pre-registration, i.e. the public disclosure of study plans before the start of the study, is an increasingly popular way of enhancing transparency and replicability in biomedical research (Nosek *et al.*, 2019; Spisak *et al.*, 2023) (Figure 2A), which could also be used to ensure the independence of the external validation sample.

However, as the concept of pre-registration was originally developed for confirmatory research, it does not fit well with the exploratory nature of the model discovery phase in typical predictive modelling endeavors.

Specifically, while pre-registration necessitates that as many parameters of the analysis as possible are fixed before data acquisition, predictive modelling studies often involve a large number of hyperparameters (e.g. model architecture, feature pre-processing steps, regularization parameters, etc.) that are not known in advance and need to be optimized during the model discovery phase. This is especially true for complex machine learning models, like deep neural networks, where the number of free parameters can easily reach tens of thousands or even more. In such cases, the pre-registration of the discovery phase would require a large number of assumptions or simplifications, which would make the process ineffective and less transparent.

Therefore, we propose to perform the pre-registration after the model discovery phase, but before the external validation (Figure 2B). In this case, more freedom is granted for the discovery phase, while the external validation remains equally conclusive, as long as the pre-registration of the external validation includes all details of the *finalized* model (including the feature pre-processing workflow). This can easily be done by attaching the data and the reproducible analysis code used during the discovery phase or, alternatively, a serialized version of the fitted model (i.e. a file that contains all model weight). We refer to such models as **registered models**. While pre-registered external validation is, to date, sparse in the predictive modelling literature (Yang *et al.*, 2022), examples of studies using the proposed registered model design do exist, see e.g. Spisak *et al.*, 2020 or Kincses *et al.*, 2024. Such studies substantiate that the **registered model** approach allows model discovery with low sample sizes (n=39 and n=25 in the two studies, respectively) and still offer an unbiased evaluation of replicability and out-of-sample generalizability, without the need for data from thousands of individuals (as recently recommended by Marek, Tervo-Clemens et. al, 2022).

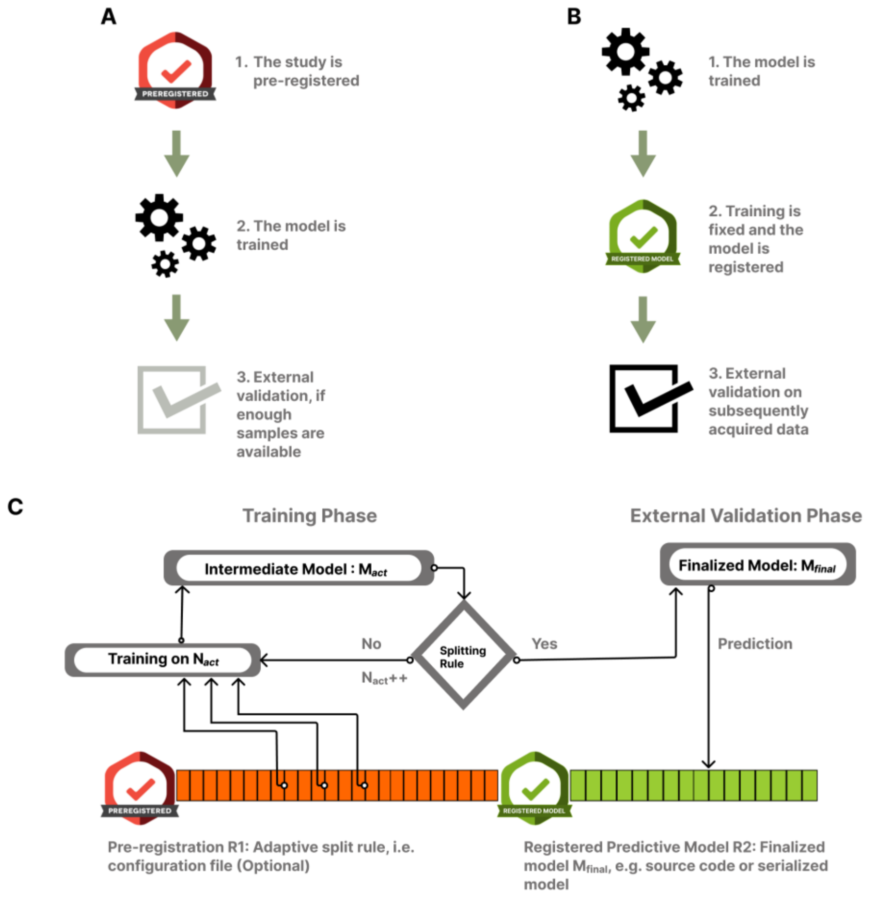

Figure 2: The registered model design and the proposed adaptive sample splitting procedure for prospective predictive modeling studies. (A) Predictive modelling combined with conventional pre-registration. In this case the pre-registration precedes data acquisition and requires fixing as many details of the analysis as possible. Given the potentially large number of coefficients to be optimized and the importance of hyperparameter optimization, conventional pre-registration exhibits a limited compatibility with predictive modelling studies. **(B)** Here we propose that in case of predictive modelling studies, public registration should only happen after the model is trained and finalized. The registration step in this case includes publicly depositing the finalized model, with all its parameters as well as all feature pre-processing steps. External validation is performed with the resulting *registered model*. This practice ensures a transparent, clear separation of model discovery and external validation. **(C)** The “registered model” design allows a flexible, adaptive splitting of the “sample size budget” into discovery and external validation phases. The proposed adaptive sample splitting procedure starts with fixing (and potentially pre-registering) a stopping rule (R1). During the discovery phase, one or more candidate models are trained and the splitting rule is repeatedly evaluated as the data acquisition proceeds. When the splitting rule “activates”, the model gets finalized (e.g. by being fit on the whole training sample) and publicly deposited/registered (R2). Finally, data acquisition continues and the prospective external validation is performed on the newly acquired data.

The adaptive splitting design

Even with registered models, the amount of data to be used for model discovery and external validation can have crucial implications on the predictive power, replicability and validity of predictive models. Here, we introduce a novel design for prospective predictive modeling studies that leverages the flexibility of model discovery granted by the registered model design. Our approach aims to adaptively determine an optimal splitting strategy during data acquisition. This strategy balances the model performance and the statistical power of the external validation (Figure 2C). The proposed design involves continuous model fitting and hyperparameter tuning throughout the discovery phase, for example, after every 10 new participants, and evaluating a ‘stopping rule’ to determine if the desired compromise between model performance and statistical power of the external validation has been achieved. This marks the end of the discovery phase and the start of the external validation phase, as well as the point at which the model must be publicly and transparently deposited or preregistered. Importantly, the preregistration should precede the continuation of data acquisition, i.e., the start of the external validation phase. In the present work, we propose and evaluate a concrete, customizable implementation for the splitting rule.

Methods and Implementation

Components of the stopping rule

The stopping rule of the proposed adaptive splitting design can be formalized as function S:

$$S_{\Phi}(X_{act}, y_{act}, \mathcal{M}) \qquad S: R^{\mathbb{2}} \longrightarrow \{True, False\} \tag{1}$$

where Φ denotes customizable parameters of the rule (detailed in the next paragraph), $X_{act} \in R^{\mathbb{2}}$ is the data (a matrix consisting of $n_{act} > 0$ observations and a fixed number of features p) and $y_{act} \in R$ is the prediction target, as acquired so far and $\mathcal{M}$ is the machine learning model to be trained. The discovery phase ends if and only if the stopping rule returns $True$.

Hard sample size thresholds

Our stopping rule is designed so that it can force a minimum size for both the discovery and the external validation samples, t_{min} and v_{min}, both being free parameters of the stopping rule.

Specifically:

$$\text{Min-rule:} \quad n_{act} \geq t_{min} \tag{2}$$

$$\text{Max-rule:} \quad n_{act} \geq n_{total} - v_{min} \tag{3}$$

where n_{act} and n_{total} are the actual sample size (e.g. participants measured so far) and the total sample size (i.e. the "sample size budget"), respectively, so that $n_{total} \geq n_{act} > 0$. Setting t_{min} and v_{min} may be useful to prevent early stopping at the beginning of the training procedure, where predictive performance and validation power estimates are not yet reliable due to the small n_{act} or to ensure that a minimal validation sample size, even if stopping criteria are never met. If t_{min} and v_{min} are set so that $t_{min} + v_{min} = n_{total}$ then our approach falls back to training a registered model with predefined discovery and validation sample sizes.

Forecasting Predictive Performance via Learning Curve Analysis

Taking internally validated performance estimates of the candidate model as a function of training sample size, also known as learning curve analysis, is a widely used approach to gain deeper insights into model discovery dynamics (see examples on Figure 1). In the proposed stopping rule, we will rely on learning curve analysis to provide estimates of the current predictive performance and the expected gain when adding new data to the discovery sample.

Performance estimates can be unreliable or noisy in many cases, for instance with low sample sizes or when using leave-one-out cross-validation (Varoquaux, 2018). To obtain stable and reliable learning curves, we propose to

calculate multiple cross-validated performance estimates from sub-samples sampled without replacement from the actual data set. The proposed procedure is detailed in Algorithm 1.

Algorithm 1 (Bootstrapped Learning Curve Analysis)

1. **Require** $\mathbf{X}_{act}, \mathbf{y}_{act}, \mathcal{M}$
2. **Set** $n_b \leftarrow$ `<number of bootstrap iterations>`
3. **For** $t \leftarrow 1$ to n_{act} *(loop over sample sizes)*
    4. **For** $i \leftarrow 1$ to n_b *(bootstrap iterations)*
        5. **Set** $\mathbf{b} \leftarrow$ sample t indices from $< 1, \dots, n_{act} >$ without replacement
        6. **Set** $\mathbf{X}_b \leftarrow \mathbf{X}_{act}[\mathbf{b}]$
        7. **Set** $\mathbf{y}_b \leftarrow \mathbf{y}_{act}[\mathbf{b}]$
        8. **Set** $\mathbf{s}[i] \leftarrow$ cross-validated performance score of $\mathcal{M}$ fitted to $(\mathbf{y}_b, \mathbf{X}_b)$
    5. **End For**
    6. **Set** $\mathbf{l}_{act}[t] \leftarrow median(\mathbf{s})$
4. **End For**
5. **Return** $\mathbf{l}_{act}$ *(bootstrapped learning curve)*

The learning curve analysis allows the discovery phase to be stopped if the expected gain in predictive performance is lower than a predefined relevance threshold and can be used for instance for stopping model training earlier in well-powered experiments and retain more data for the external validation phase. Specifically, the stopping rule S will return $True$ if the *Min-rule* (Eq. 2) is $True$ or the following is true:

$$\text{Performance-rule:} \quad \widehat{s_{total}} - s_{act} \leq s_{min} \tag{4}$$

where s_{act} is the actual bootstrapped predictive performance score (i.e. the last element of l_{act}, as returned by Algorithm 1, $\widehat{s_{total}}$ is a estimate of the (unknown) predictive performance s_{total} (i.e. the predictive performance of the model trained on the whole sample size) and s_{min} is the smallest predictive effect of interest. Note that this

parameter configuration essentially switches off the performance rule for our main analysis ($s_{min} = 0$, but see Supplementary material, figure 7, for an analysis of the effect of the performance rule) and ensures that even in case of very small simulated sample size budgets, the training sample is suitable for cross-validation ($v\_min = 12$).

While s_{total} is typically unknown at the time of evaluating the stopping rule S, there are various approaches of obtaining an estimate $\widehat{s_{total}}$. In the base implementation of AdaptiveSplit, we stick to a simple method: we extrapolate the learning curve l_{act} based on its tangent line at n_{act}, i.e. assuming that the latest growth rate will remain constant for the remaining samples. While in most scenarios this is an overly optimistic estimate, it still provides a useful upper bound for the maximally achievable predictive performance with the given sample size and can successfully detect if the learning curve has already reached a flat plateau (like on Figure 1C).

Statistical power of the external validation sample

Even if the learning curve did not reach a plateau, we still need to make sure that we stop the discovery phase early enough to save a sufficient amount of data for a successful external validation from our sample size budget. Given the actual predictive performance estimate s_{act} and the size of the remaining, to-be-acquired sample $s_{total} - s_{act}$, we can estimate the probability that the external validation correctly rejects the null hypothesis (i.e. zero predictive performance). This type of analysis, known as power calculation, allows us to determine the optimal stopping point that guarantees the desired statistical power during the external validation. Specifically, the stopping rule S will return $True$ if the $Performance\text{-}rule$ (Eq. 4) is $False$ and the following is true:

$$\text{Power-rule:} \quad POW_{\alpha}(s_{act}, n_{val}) \leq v_{pow} \tag{5}$$

where $POW_{\alpha}(s, n)$ is the power of a validation sample of size n to detect an effect size of s and $n_{val} = n_{total} - n_{act}$ is the size of the validation sample if stopping, i.e. the number of remaining (not yet measured) participants in the experiment. Given that machine learning model predictions are often non-normally distributed (Spisak, 2022), our implementation is based on a bootstrapped power analysis for permutation tests, as shown in

Algorithm 2. Our implementation is, however, simple to extend with other parametric or non-parametric power calculation techniques.

Algorithm 2 **(Calculation of the Power-rule)**

1. **Require** $\mathbf{X}_{act}, \mathbf{y}_{act}, n_{validation}, \mathcal{M}, \alpha$
2. **Set** $n_b \leftarrow$ `<number of bootstrap iterations>`
3. **Set** $n_\pi \leftarrow$ `<number of permutations>`
4. **Set** $\hat{\mathbf{y}}_{act} \leftarrow$ cross-validated prediction from $\mathbf{X}_{act}$with$\mathcal{M}$
    5. **For** $i \leftarrow 1$ to n_b
        6. **Set** $\mathbf{b} \leftarrow$ sample t indices from $< 1, \ldots, n_{val} >$ with replacement
        7. **Set** $\mathbf{y}_b \leftarrow \mathbf{y}_{act}[\mathbf{b}]$
        8. **Set** $\hat{\mathbf{y}}_b \leftarrow \hat{\mathbf{y}}_{act}[\mathbf{b}]$
        9. **Set** $r_{obs} = correlation(\mathbf{y}_b, \hat{\mathbf{y}}_b)$
        10. **For** $j \leftarrow 1$ to n_p
            11. **Set** $\boldsymbol{\pi} \leftarrow$ permute$(< 1, \ldots, n_{val} >)$
            12. **Set** $\mathbf{y}_\pi \leftarrow \mathbf{y}_b[\boldsymbol{\pi}]$
            13. **Set** $\hat{\mathbf{y}}_\pi \leftarrow \hat{\mathbf{y}}_b[\boldsymbol{\pi}]$
            14. **Set** $\mathbf{r}_{null}[j] = correlation(\mathbf{y}_\pi, \hat{\mathbf{y}}_\pi)$
        11. **End For**
        12. **Set** $\mathbf{p}[i] \leftarrow \#(\mathbf{r}_{null} > r_{obs})/n_{perm}$
    6. **End For**
5. **Set** $power = \#(\mathbf{p} < \alpha)/n_b$
6. **Return** $power$

Note that depending on the aim of external validation, the *Power-rule* can be swapped to, or extended with, other conditions. For instance, if we are interested in accurately estimating the predictive effect size, we could condition the stopping rule on the width of the confidence interval for the prediction performance.

Calculating the validation power (Algorithm 2) for all available sample sizes ($n = 1 \ldots n_{act}$) defines the so-called "validation power curve" (see Figure 1 and Supplementary Figures 2, 4 and 6), that represents the expected ratio of true positive statistical tests on increasing sample size calculated on the external validation set. Various extrapolations of the power curve can predict the expected stopping point during the course of the experiment.

Stopping Rule

Our proposed stopping rule integrates the Min-rule, the Max-rule, the Performance-rule and the Power-rule in the following way:

$$S_{\Phi}(X_{act}, y_{act}, \mathcal{M}) = \text{Min-rule} \quad AND$$
$$\Big(\text{Max-rule} \quad OR$$
$$\text{Performance-rule} \quad OR$$
$$\text{Power-rule} \Big) \tag{6}$$

where $\Phi =< t_{min}, v_{min}, s_{min}, v_{pow}, \alpha >$ are parameters of the stopping rule: minimum training sample size, minimum validation sample size, minimum effect of interest and target power for the external validation and the significance threshold, respectively.

We have implemented the proposed stopping rule in the Python package "*adaptivesplit*" (Gallitto et al., n.d.). The package can be used together with a wide variety of machine learning tools and provides an easy-to-use interface to work with scikit-learn (Pedregosa *et al.*, 2012) models.

Empirical evaluation

We evaluate the proposed stopping rule, as implemented in the package *adaptivesplit* (Gallitto et al., n.d.), in four publicly available datasets; the Autism Brain Imaging Data Exchange (ABIDE; Di Martino *et al.*, 2013), the Human Connectome Project (HCP; Van Essen *et al.*, 2013), the Information eXtraction from Images (IXI; Hill *et al.*, 2005) and the Breast Cancer Wisconsin (BCW; Street *et al.*, 1993) datasets (Fig. 3).

ABIDE

We obtained preprocessed data from Autism Brain Imaging Data Exchange (ABIDE) dataset (Di Martino *et al.*, 2013) involving the resting-state data of 866 participants (Autism Spectrum Disorder: 402, neurotypical control: 464). Pre-processed regional time-series data were obtained as shared by Dadi *et al.*, 2019, which were based on image data provided by the Pre-processed Connectome Project (Craddock *et al.*, 2013a), pre-processed using the C-PAC pipeline (Craddock et al., 2013b; Preprocessed Connectomes Project, n.d.), without global signal regression. Tangent correlation across the time series of the n=122 regions of the BASC brain parcellation (Multi-level bootstrap analysis of stable clusters; Bellec *et al.*, 2010) was computed with nilearn (Abraham et al., 2014). The resulting functional connectivity estimates were considered features for a predictive model of autism diagnosis.

HCP

The Human Connectome Project dataset contains imaging and behavioral data of approximately 1,200 healthy subjects (Van Essen *et al.*, 2013). Pre-processed resting state functional magnetic resonance imaging (fMRI) connectivity data (partial correlation of the mean regional timeseries of 100 brain parcels derived via independent component analysis; Glasser *et al.*, 2013 as published with the HCP1200 release (N=999 participants with functional connectivity data) were used to build models that predict individual fluid intelligence scores (Gf), measured with Penn Progressive Matrices (Duncan *et al.*, 2000). The minimal preprocessing pipelines of Glasser et al. for structural, functional, and diffusion MRI that were developed by the HCP and included spatial artifact/distortion removal, surface generation, cross-modal registration, and alignment to standard space. These pipelines were specially designed to capitalize on the high-quality data offered by the HCP.

IXI

The IXI dataset is published by the Neuroimage Analysis Center, from Imperial College London, in the United Kingdom, and it is part of the project Brain Development. It consists of approximately 600 structural MRI images from a diverse population of healthy individuals, including both males and females across a wide age range. The dataset contains high-resolution brain images from three different MRI scanners (Philips Intera 3T, Philips

Gyroscan Intera 1.5T and GE 1.5T) and associated demographic information, making it suitable for studying age-related changes in brain structure and function. Structural pre-processing of T1-weighted images was conducted using FreeSurfer (Fischl, 2012) software (version 6.0), run with default parameters, focusing on grey matter volume. The procedure included motion correction, skull stripping, removal of the cerebellum and brain stem, intensity correction, segmentation, tessellation, smoothing and topology correction (Kotikalapudi, 2024). Cortical volume of brain regions was measured using the Desikan-Killiany brain atlas (Desikan et al., 2006), producing 68 regional volume measures (34 per hemisphere, measured in mm^3).

BCW

The Breast Cancer Wisconsin (BCW, Street et al., 1993) dataset contains diagnostic features computed from digitized images of fine needle aspirates (FNA) of breast masses. The FNA procedure involves using a thin, hollow needle to extract cells from a suspicious area of breast tissue. These cells are then smeared onto glass slides, stained to highlight cellular structures, and scanned to create digital images. Specialized software analyses these images (Wolberg et al., 1994) to extract 30 different features, which quantify various morphological characteristics of the cell nuclei, such as size, shape, and texture. These features are used to create a predictive model for breast cancer diagnosis, with the target variable being the diagnosis categorized as malignant (M) or benign (B).

The chosen datasets include both classification and regression tasks and span a wide range in terms of number of participants, number of predictive features, achievable predictive effect size and data homogeneity (see Supplementary Figures 1-6). Our analyses aimed to contrast the proposed adaptive splitting method with the application of fixed training and validation sample sizes, specifically using 50, 60 or 90% of the total sample size for discovery and the rest for external validation. We simulated various "sample size budgets" (total sample sizes, n_{total}) with random sampling without replacement. For a given total sample size, we simulated the prospective data acquisition procedure by incrementing n_{act}; starting with 10% of the total sample size and going up with increments of five. In each step, the stopping rule was evaluated with "AdaptiveSplit", fitting a Ridge model (for regression tasks; HCP and IXI datasets) or a L2-regularized logistic regression (for classification tasks; ABIDE and BCW datasets). Model fit always consisted of a cross-validated fine-tuning of the α regularization parameter ($\alpha \in \{0.1, 1, 10\}$), resulting in a nested cv estimate of prediction performance and validation power. Robust

estimates (and confidence intervals) were obtained with bootstrapping, as described in Algorithm 1 and Algorithm 2. This procedure was iterated until the stopping rule returned True. The corresponding sample size was then considered the final discovery sample. With all four splitting approaches (adaptive, Pareto, Half-split, 90-10% split), we trained the previously described Ridge or regularized logistic regression model on the discovery sample and obtained predictions for the sample left out for external validation. This whole procedure was repeated 100 times for each simulated sample size budget in each dataset, to estimate the confidence intervals for the models performance in the external validation and its statistical significance. In all analyses, the adaptive splitting procedure is performed with a target power of $v_{pow} = 0.8$, an $alpha = 0.05$, $t_{tmin} = n_{total}/3$, $v_{min} = 12$, $s_{min} = 0$. P-values were calculated using a permutation test with 5000 permutations.

Results

The results of our empirical analyses of four large, openly available datasets confirmed that the proposed adaptive splitting approach can successfully identify the optimal time to stop acquiring data for training and maintain a good compromise between maximizing both predictive performance and external validation power with any sample size budget.

In all four samples, the applied models yielded a statistically significant predictive performance at much lower sample sizes than the total size of the dataset, i.e. all datasets were well powered for the analysis. Thus, when reporting our results, we focused on the most realistic scenarios and omitted sample size budgets that were powered too low (neither of the splitting strategies leads to significant model performance) or too high (prediction performance plateaus with all splitting strategies) for any meaningful comparison between splitting strategies. Trained on the full sample size with cross-validation, the models displayed the following performances: functional brain connectivity from the HCP dataset explained 13% of the variance in cognitive abilities; structural MRI data (gray matter probability maps) in the IXI dataset explained 48% in age; classification accuracy was 65.5% for autism diagnosis (functional brain connectivity) in the ABIDE dataset and 92% for breast cancer diagnosis in the BCW dataset.

The datasets varied not only in the achievable predictive performance but also in the shape of the learning curve, with different sample sizes and thus, they provided a good opportunity to evaluate the performance of our stopping rule in various circumstances (Supplementary Figures 1-6).

We found that adaptively splitting the data provided external validation performances that were comparable to the commonly used Pareto split (80-20%) in most cases (Figure 3, left column). From the fixed splitting approaches, the half-split assigns the least samples from the total sample size budget to the training phase (50%). Thus, the resulting model is trained on less data than with other strategies, typically resulting in a smaller l_{act}. While this lower effect size should in general result in lower statistical power during the external validation phase, the half-split approach can counterbalance this with the larger sample size remaining for external validation. Our analysis shows, that this happens in almost all of the cases, hinting that in research scenarios where the expected predictive performance is low, researchers should either use the proposed adaptive splitting procedure, or aim for a relatively large pre-fixed external validation sample. In contrast, 90-10% tended to display only slightly higher performances than the Pareto and the Adaptive splitting techniques, in most cases. This small achievement came with a big cost in terms of the statistical power in the external validation sample, where the 90-10% split very often gave inconclusive results ($p \geq 0.05$) (Figure 3, right column), especially with low sample size budgets. Although to a lesser degree, Pareto split also frequently failed to yield a conclusive external validation with small total sample sizes. In addition to the Pareto, half-split, and 90-10% splitting strategies, we also evaluated alternative split ratios (75-25% and 70-30%), which are commonly used in the literature. The 75-25% split demonstrated performance comparable to the Pareto and adaptive splitting techniques, although, similarly to Pareto, it struggled to achieve statistical significance at smaller sample sizes. In contrast, the 70-30% split exhibited good statistical significance at the cost of lower overall performance, comparable to the trend observed with the half-split strategy (see Supplementary material, Figure 13). Adaptive splitting (as well as half-split) provided sufficient statistical power for the external validation in most cases. This was achieved by applying different strategies in different scenarios. In case of low total sample sizes, it retained a larger proportion of the sample for the external validation phase in order to achieve sufficient power, up to using 79% of the data for external validation. On the other hand, if the total sample size budget allowed it, adaptive splitting let the predictive model benefit from larger training samples, retaining 8% or less of the data for external validation in such cases.

Focusing only on cases with a successful, conclusive external validation, the proposed adaptive splitting strategy provided an external validation performance comparable to the alternative fixed splitting strategies, in all cases

where the external validation was conclusive (statistically significant). Furthermore, in contrast to the investigated fixed splitting strategies, the proposed splitting strategy yields solid guarantees for the success of the external validation phase, independent of the sample size budget.

**Classification**	**BCW**					**ABIDE**				
Sample sizes	49	65	86	113	150	400	442	489	542	599
Adaptive Splits	21-79	33-67	48-52	67-33	92-08	41-59	49-51	59-41	71-29	82-18
Discovery Scores	0.888	0.921	0.933	0.938	0.944	0.614	0.624	0.633	0.640	0.644
External Validation scores	0.896	0.927	0.935	0.941	0.944	0.626	0.634	0.634	0.643	0.655
Statistical Significance	0.036	0.032	0.033	0.024	0.041	0.018	0.014	0.023	0.027	0.017
**Regression**	**HCP**					**IXI**				
Sample sizes	242	272	305	343	384	49	65	86	113	150
Splits	44-56	53-47	64-36	76-24	89-11	21-79	25-75	40-60	61-39	89-11
Discovery Scores	-15.35	-15.20	-15.10	-15.02	-14.89	-12.01	-11.93	-11.72	-11.54	-11.16
External Validation scores	-15.24	-15.13	-15.09	-14.82	-14.82	-11.95	-12.08	-11.40	-11.12	-10.74
Statistical Significance	0.012	0.016	0.019	0.033	0.029	0.070	0.031	0.014	0.021	0.041

Table.1: Performance results of the "adaptivesplit" algorithm for each dataset across the different sample sizes (n_{act}). The fraction of discovery samples (orange) and external validation samples (green) is shown for each split. For each n_{act}, the relative accuracy (for classification tasks) or negative mean absolute error (for regression tasks) is reported, along with statistical significance (p-value), providing a comprehensive overview of the algorithm's performance across different datasets and sample sizes.

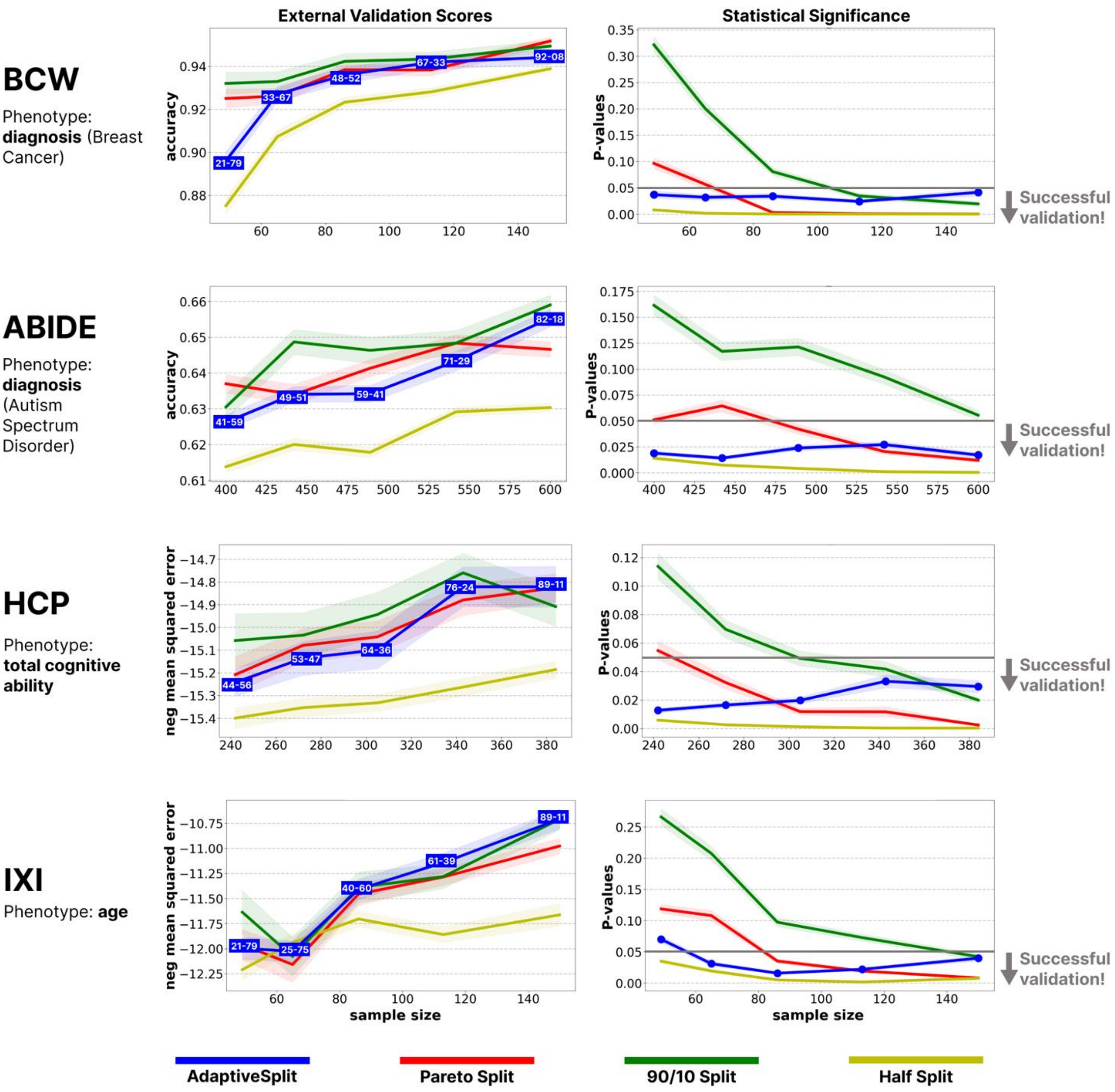

Figure 3: The proposed adaptive splitting approach provides a good compromise between predictive performance and statistical power of the external validation. The left and right column shows the comparison of splitting methods on external validation performance and p-values, respectively, at various n_{total}. Confidence intervals are based on 100 repetitions of the analyses. The adaptive splitting approach (blue) provides a good compromise between predictive performance and statistical power of the external validation. The Pareto split (red) provides similar external validation performances to adaptive splitting; however it often fails to provide conclusive results due to an insufficient sample size during external validation, especially in case of a limited sample size budget. The 90-10% split (green) provides only slightly higher performances than the Pareto and the Adaptive splitting techniques, but it very often gives inconclusive results ($p \geq 0.05$) in the

external validation sample. Half-split (yellow) tends to provide worse predictive performance due to the too small discovery sample.

Discussion

Here we have proposed "registered models", a novel design for prospective predictive modeling studies that allows flexible model discovery and trustworthy prospective external validation by fixing and publicly depositing the model after the discovery phase. Furthermore, capitalizing on the flexibility during model discovery with the registered model design, we have proposed a stopping rule for adaptively splitting the sample size budget into discovery and external validation phases. These approaches together provide a robust and flexible framework for predictive modeling studies and address several common issues in the field, including overfitting, effect size inflation as well as the lack of reliability and reproducibility.

Registered models provide a clear and transparent separation between the discovery and external validation phases, which is essential for ensuring the independence of the external validation data. Thereby, they provide a straightforward solution to several of the widely discussed issues and pitfalls of predictive model development (Efron, 1983; Sui *et al.*, 2020; Varoquaux & Cheplygina, 2022; Marek *et al.*, 2022; Spisak *et al.*, 2023). With registered models, external validation estimates are guaranteed to be free of information leakage (Kapoor & Narayanan, 2023) and provide an unbiased estimate of the model's predictive performance.

With registered models, the question of how the total sample size budget should be distributed between the discovery and external validation phase remains of central importance for the optimal use of available resources (scanning time, budget, limitations in participant recruitment) (Archer *et al.*, 2020; Riley *et al.*, 2021; Marek *et al.*, 2022; Spisak *et al.*, 2023; Rosenberg & Finn, 2022; Thirion, 2023; Makowski *et al.*, 2023; Supplementary Table 1). Optimal sample sizes are often challenging to determine prior to the study. The proposed adaptive splitting procedure promises to provide a solution in such cases by allowing the sample size to be adjusted during the data acquisition process, based on the observed performance of the model trained on the already available data. We performed a thorough evaluation of the proposed adaptive splitting procedure on data from more than 3000 participants from four publicly available datasets. We found that the proposed adaptive splitting approach can successfully identify the optimal time to stop acquiring data for training and maintain a good compromise between maximizing both predictive performance and external validation power with any "sample size budget".

When contrasting splitting approaches based on fixed validation size with the proposed adaptive splitting technique, using the latter was always the preferable strategy to maximize power and statistical significance during external validation. The benefit of adaptively splitting the data acquisition for training and validation provides the largest benefit in lower sample size regimes. In case of larger total sample size budgets, the fixed Pareto split (20-80%) provided also good results, giving similar external validation performances to adaptive splitting, without having to repeatedly re-train the model during data acquisition. Thus, for moderate to large sample sizes and well powered models, the Pareto split might be a good alternative to the adaptive splitting approach, especially if the computational resources for re-training the model are limited.

Of note, the presented implementation of adaptive data splitting aims to maximize the discovery sample (and minimize the external validation sample) in order to achieve the highest possible performance together with a conclusive (statistically significant) external validation. However, the resulting external performance estimates will still be subject of sampling variance. If the aim is to provide more reliable estimates of the predictive effect size in the external validation, the power-rule in the proposed approach can be modified so that it stops the discovery phase when a desired confidence interval width for the external effect size estimate is reached.

The proposed adaptive splitting design can advance the development of predictive models in several ways. Firstly, it provides a simple way to perform both model discovery and initial external validation in a single study. Furthermore, it promotes the public deposition (registration) of models at an early stage of the study, enhancing transparency, reliability and replicability. Finally, it provides a flexible approach to data splitting, which can be adjusted according to the specific needs of the study.

In conclusion, registered models provide a simple approach to guarantee the independence of model discovery and external validation and for the development and initial evaluation of registered models with unknown power, the introduced adaptive splitting procedure provides a robust and flexible approach to determine the optimal ratio of data to be used for model discovery and external validation. Together, registered models and the adaptive splitting procedure, address several common issues in the field, including overfitting, cross-validation failure, and boost the reliability and reproducibility.

Data and Code availability

Empirical analysis was based on data provided by the following sources: (i) the Human Connectome Project (WU-Minn Consortium, principal investigators: D. Van Essen and K. Ugurbil; 1U54MH091657), funded by the 16 National Institutes of Health (NIH) institutes and centers that support the NIH Blueprint for Neuroscience Research, (ii) the ABIDE consortium (Di Martino *et al.*, 2013), (iii) the Imperial college London (IXI, principal investigator: Hill D.L., other investigators: Williams S.C.R., Smith S.M., Hawkes, D; GR/S21533/02) and (iv) the University of Wisconsin (Street et al., 1993). Raw and pre-processed data used in the present study are publicly available for download in their respective repositories:

- ABIDE raw data (Di Martino *et al.*, 2013; available at https://fcon_1000.projects.nitrc.org/indi/abide/):
- ABIDE preprocessed dataset (Dadi et al., 2019; available at https://osf.io/hc4md)
- HCP1200 raw data (Van Essen et al., 2013; available at https://db.humanconnectome.org/)
- HCP1200 preprocessed data (Glasser *et al.*, 2013; available at https://www.humanconnectome.org/)
- BCW preprocessed dataset (Street *et al.*, 1993; available at https://www.kaggle.com/datasets/uciml/breast-cancer-wisconsin-data).
- IXI raw data (Hill et al., 2005; https://brain-development.org/ixi-dataset/).
- IXI preprocessed dataset (Kotikalapudi, 2024; available at https://zenodo.org/records/11635168).

The Python implementation of the "adaptivesplit" package is publicly available on GitHub (Gallitto et al., n.d.; https://github.com/pni-lab/adaptivesplit). Additionally, the Python scripts and data used for the analyses presented in this manuscript can be accessed in the following GitHub repository: https://github.com/pni-lab/AdaptiveSplitAnalysis.

Availability of supporting code and requirements

Project name: adaptivesplit

Project home page: https://github.com/pni-lab/adaptivesplit

Operating system(s): Platform independent

Programming language: Python

Other requirements: Python 3.9 or higher

License: GNU General public licence, version 3, 29 June 2007 (GPL-3.0)

RRID: SCR_025888

bio.tools: bio.tools:adaptivesplit

Acknowledgements

The work is funded by the Deutsche Forschungsgemeinschaft (DFG, German Research Foundation) - Project-ID 422744262 - TRR 289 (Gefördert durch die Deutsche Forschungsgemeinschaft (DFG) – Projektnummer 422744262 – TRR 289).

Competing interests

The authors declare that they have no competing interests.

References

Abraham, A., Pedregosa, F., Eickenberg, M., Gervais, P., Mueller, A., Kossaifi, J., ... & Varoquaux, G. (2014). Machine learning for neuroimaging with scikit-learn. Frontiers in neuroinformatics, 8, 14.

Archer, L., Snell, K. I. E., Ensor, J., Hudda, M. T., Collins, G. S., & Riley, R. D. (2020). Minimum sample size for external validation of a clinical prediction model with a continuous outcome. *Statistics in Medicine*, *40*(1), 133–146. 10.1002/sim.8766

Bellec, P., Rosa-Neto, P., Lyttelton, O. C., Benali, H., & Evans, A. C. (2010). Multi-level bootstrap analysis of stable clusters in resting-state fMRI. *NeuroImage*, *51*(3), 1126–1139. 10.1016/j.neuroimage.2010.02.082

Collins, G. S., de Groot, J. A., Dutton, S., Omar, O., Shanyinde, M., Tajar, A., Voysey, M., Wharton, R., Yu, L.-M., Moons, K. G., & Altman, D. G. (2014). External validation of multivariable prediction models: a systematic review of methodological conduct and reporting. *BMC Medical Research Methodology*, *14*(1). 10.1186/1471-2288-14-40

Craddock, C., Benhajali, Y., Chu, C., Chouinard, F., Evans, A., Jakab, A., & Bellec, P. (2013a). The neuro bureau preprocessing initiative: open sharing of preprocessed neuroimaging data and derivatives. Frontiers in Neuroinformatics, 7(27), 5. 10.3389/conf.fninf.2013.09.00041

Craddock, C., Sikka, S., Cheung, B., Khanuja, R., Ghosh, S. S., Yan, C., … & Milham, M. (2013b). Towards automated analysis of connectomes: The configurable pipeline for the analysis of connectomes (C-PAC). Frontiers in Neuroinformatics, 42 (10.3389).

Dadi, K., Rahim, M., Abraham, A., Chyzhyk, D., Milham, M., Thirion, B., & Varoquaux, G. (2019). Benchmarking functional connectome-based predictive models for resting-state fMRI. *NeuroImage*, *192*, 115–134. 10.1016/j.neuroimage.2019.02.062

Desikan, R. S., Ségonne, F., Fischl, B., Quinn, B. T., Dickerson, B. C., Blacker, D., ... & Killiany, R. J. (2006). An automated labeling system for subdividing the human cerebral cortex on MRI scans into gyral based regions of interest. Neuroimage, 31(3), 968-980.

Di Martino, A., Yan, C.-G., Li, Q., Denio, E., Castellanos, F. X., Alaerts, K., Anderson, J. S., Assaf, M., Bookheimer, S. Y., Dapretto, M., Deen, B., Delmonte, S., Dinstein, I., Ertl-Wagner, B., Fair, D. A., Gallagher, L., Kennedy, D. P., Keown, C. L., Keysers, C., … Milham, M. P. (2013). The autism brain imaging data exchange:

towards a large-scale evaluation of the intrinsic brain architecture in autism. *Molecular Psychiatry*, *19*(6), 659–667. 10.1038/mp.2013.78

Duncan, J., Seitz, R. J., Kolodny, J., Bor, D., Herzog, H., Ahmed, A., Newell, F. N., & Emslie, H. (2000). A Neural Basis for General Intelligence. *Science*, *289*(5478), 457–460. 10.1126/science.289.5478.457

Efron, B. (1983). Estimating the Error Rate of a Prediction Rule: Improvement on Cross-Validation. *Journal of the American Statistical Association*, *78*(382), 316–331. 10.1080/01621459.1983.10477973

Efron, B., & Tibshirani, R. J. (1994). *An Introduction to the Bootstrap*. Chapman. 10.1201/9780429246593

Fischl, B. (2012). FreeSurfer. *NeuroImage*, *62*(2), 774–781. 10.1016/j.neuroimage.2012.01.021

Gallitto, G., Englert, R., Kincses, B., Kotikalapudi, R., Li, J., Hoffschlag, K., Bingel, U., Spisak, T. (n.d.). *adaptivesplit* [Computer software]. GitHub. Retrieved May 27, 2024 from https://github.com/pni-lab/adaptivesplit

Glasser, M. F., Sotiropoulos, S. N., Wilson, J. A., Coalson, T. S., Fischl, B., Andersson, J. L., Xu, J., Jbabdi, S., Webster, M., Polimeni, J. R., Van Essen, D. C., & Jenkinson, M. (2013). The minimal preprocessing pipelines for the Human Connectome Project. *NeuroImage*, *80*, 105–124. 10.1016/j.neuroimage.2013.04.127

Hill, D.L., Williams, S.C.R., Smith, S.M, Hawkes, D. (2005). Information eXtraction from Images (IXI). Available at http://brain-development.org/ixi-dataset/

Ho, S. Y., Phua, K., Wong, L., & Bin Goh, W. W. (2020). Extensions of the External Validation for Checking Learned Model Interpretability and Generalizability. *Patterns*, *1*(8), 100129. 10.1016/j.patter.2020.100129

Hosseini, M., Powell, M., Collins, J., Callahan-Flintoft, C., Jones, W., Bowman, H., & Wyble, B. (2020). I tried a bunch of things: The dangers of unexpected overfitting in classification of brain data. *Neuroscience & Biobehavioral Reviews*, *119*, 456–467. 10.1016/j.neubiorev.2020.09.036

Kapoor, S., & Narayanan, A. (2023). Leakage and the reproducibility crisis in machine-learning-based science. *Patterns*, *4*(9), 100804. 10.1016/j.patter.2023.100804

Kincses, B., Forkmann, K., Schlitt, F., Pawlik, R., Schmidt, K., Timmann, D., Elsenbruch, S., Wiech, K., Bingel, U., & Spisak, T. (2024). *An externally validated resting-state brain connectivity signature of pain-related learning*. Accepted in Communications Biology, Preprint: 10.31219/osf.io/utkbv

Kotikalapudi, R. (2024). IXI – Information eXtraction from Images | Cortical Volume [Data set]. Zenodo. https://doi.org/10.5281/zenodo.11635168

Lee, J.-J., Kim, H. J., Čeko, M., Park, B., Lee, S. A., Park, H., Roy, M., Kim, S.-G., Wager, T. D., & Woo, C.-W. (2021). A neuroimaging biomarker for sustained experimental and clinical pain. *Nature Medicine*, *27*(1), 174–182. 10.1038/s41591-020-1142-7

Lipovetsky, S. (2009). Pareto 80/20 law: derivation via random partitioning. *International Journal of Mathematical Education in Science and Technology*, *40*(2), 271–277. 10.1080/00207390802213609

Makowski, C., Brown, T. T., Zhao, W., Hagler, D. J., Parekh, P., Garavan, H., Nichols, T. E., Jernigan, T. L., & Dale, A. M. (2023). *Leveraging the Adolescent Brain Cognitive Development Study to improve behavioral prediction from neuroimaging in smaller replication samples*. 10.1101/2023.06.16.545340

Marek, S., Tervo-Clemmens, B., Calabro, F. J., Montez, D. F., Kay, B. P., Hatoum, A. S., Donohue, M. R., Foran, W., Miller, R. L., Hendrickson, T. J., Malone, S. M., Kandala, S., Feczko, E., Miranda-Dominguez, O., Graham, A. M., Earl, E. A., Perrone, A. J., Cordova, M., Doyle, O., … Dosenbach, N. U. F. (2022). Reproducible brain-wide association studies require thousands of individuals. *Nature*, *603*(7902), 654–660. 10.1038/s41586-022-04492-9

Nosek, B. A., Beck, E. D., Campbell, L., Flake, J. K., Hardwicke, T. E., Mellor, D. T., van 't Veer, A. E., & Vazire, S. (2019). Preregistration Is Hard, And Worthwhile. *Trends in Cognitive Sciences*, *23*(10), 815–818. 10.1016/j.tics.2019.07.009

Pedregosa, F., Varoquaux, G., Gramfort, A., Michel, V., Thirion, B., Grisel, O., Blondel, M., Müller, A., Nothman, J., Louppe, G., Prettenhofer, P., Weiss, R., Dubourg, V., Vanderplas, J., Passos, A., Cournapeau, D., Brucher, M., Perrot, M., & Duchesnay, É. (2012). *Scikit-learn: Machine Learning in Python*. 10.48550/ARXIV.1201.0490

Poldrack, R. A., Huckins, G., & Varoquaux, G. (2020). Establishment of Best Practices for Evidence for Prediction: A Review. *JAMA Psychiatry*, *77*(5), 534. 10.1001/jamapsychiatry.2019.3671

Preprocessed Connectomes Project. (n.d.). Preprocessing with C-PAC. http://preprocessed-connectomes-project.org/abide/cpac.html

Prosperi, M., Guo, Y., Sperrin, M., Koopman, J. S., Min, J. S., He, X., Rich, S., Wang, M., Buchan, I. E., & Bian, J. (2020). Causal inference and counterfactual prediction in machine learning for actionable healthcare. *Nature Machine Intelligence*, *2*(7), 369–375. 10.1038/s42256-020-0197-y

Raykar, V. C., & Saha, A. (2015). Data Split Strategiesfor Evolving Predictive Models. In *Lecture Notes in Computer Science* (pp. 3–19). Springer International Publishing. 10.1007/978-3-319-23528-8_1

Riley, R. D., Debray, T. P. A., Collins, G. S., Archer, L., Ensor, J., van Smeden, M., & Snell, K. I. E. (2021). Minimum sample size for external validation of a clinical prediction model with a binary outcome. *Statistics in Medicine*, *40*(19), 4230–4251. 10.1002/sim.9025

Rosenberg, M. D., & Finn, E. S. (2022). How to establish robust brain–behavior relationships without thousands of individuals. *Nature Neuroscience*, *25*(7), 835–837. 10.1038/s41593-022-01110-9

Spisak, T. (2022). Statistical quantification of confounding bias in machine learning models. *GigaScience*, *11*. 10.1093/gigascience/giac082

Spisak, T., Bingel, U., & Wager, T. D. (2023). Multivariate BWAS can be replicable with moderate sample sizes. *Nature*, *615*(7951), E4–E7. 10.1038/s41586-023-05745-x

Spisak, T., Kincses, B., Schlitt, F., Zunhammer, M., Schmidt-Wilcke, T., Kincses, Z. T., & Bingel, U. (2020). Pain-free resting-state functional brain connectivity predicts individual pain sensitivity. *Nature Communications*, *11*(1). 10.1038/s41467-019-13785-z

Steyerberg, E. W., & Harrell, F. E. (2016). Prediction models need appropriate internal, internal–external, and external validation. *Journal of Clinical Epidemiology*, *69*, 245–247. 10.1016/j.jclinepi.2015.04.005

Street, W. N., Wolberg, W. H., & Mangasarian, O. L. (1993). Nuclear feature extraction for breast tumor diagnosis In R. S. Acharya & D. B. Goldgof (Eds.), *Biomedical Image Processing and Biomedical Visualization*. SPIE. 10.1117/12.148698

Sui, J., Jiang, R., Bustillo, J., & Calhoun, V. (2020). Neuroimaging-based Individualized Prediction of Cognition and Behavior for Mental Disorders and Health: Methods and Promises. *Biological Psychiatry*, *88*(11), 818–828. 10.1016/j.biopsych.2020.02.016

Thirion, B. (2023). On the statistics of brain/behavior associations. *Aperture Neuro*. 10.52294/51f2e656-d4da-457e-851e-139131a68f14

Van Essen, D. C., Smith, S. M., Barch, D. M., Behrens, T. E. J., Yacoub, E., & Ugurbil, K. (2013). The WU-Minn Human Connectome Project: An overview. *NeuroImage*, *80*, 62–79. 10.1016/j.neuroimage.2013.05.041

Varoquaux, G. (2018). Cross-validation failure: Small sample sizes lead to large error bars. *NeuroImage*, *180*, 68–77. 10.1016/j.neuroimage.2017.06.061

Varoquaux, G., & Cheplygina, V. (2022). Machine learning for medical imaging: methodological failures and recommendations for the future. *Npj Digital Medicine*, *5*(1). 10.1038/s41746-022-00592-y

Wolberg, W., Street, W. N., & Mangasarian, O. (1994). Breast cancer diagnosis and prognosis via linear programming.

Woo, C.-W., Chang, L. J., Lindquist, M. A., & Wager, T. D. (2017). Building better biomarkers: brain models in translational neuroimaging. *Nature Neuroscience*, *20*(3), 365–377. 10.1038/nn.4478

Yang, C., Kors, J. A., Ioannou, S., John, L. H., Markus, A. F., Rekkas, A., de Ridder, M. A. J., Seinen, T. M., Williams, R. D., & Rijnbeek, P. R. (2022). Trends in the conduct and reporting of clinical prediction model development and validation: a systematic review. *Journal of the American Medical Informatics Association*, *29*(5), 983–989. 10.1093/jamia/ocac002

Yu, A. C., Mohajer, B., & Eng, J. (2022). External Validation of Deep Learning Algorithms for Radiologic Diagnosis: A Systematic Review. *Radiology: Artificial Intelligence*, *4*(3). 10.1148/ryai.210064

Click here to access/download
Supplementary Material
adaptivesplit_supplementary.pdf

Manuscript copy with revisions

Click here to access/download;Personal Cover;adaptivesplit_review_corrections.docx

External validation of machine learning models - registered models and adaptive sample splitting

Giuseppe Gallitto[1,2], Robert Englert[1,3], Balint Kincses[1,2], Raviteja Kotikalapudi[1,2], Jialin Li[1,2,4], Kevin Hoffschlag[1,2], Ulrike Bingel[1,2], Tamas Spisak[1,3]*

1 Center for Translational Neuro- and Behavioral Sciences (C-TNBS), University Medicine Essen, Germany

2 Department of Neurology, University Medicine Essen, Germany

3 Department of Diagnostic and Interventional Radiology and Neuroradiology, University Medicine Essen, Germany

4 Max Planck School of Cognition, Leipzig, Germany

** Corresponding author*

Abstract

Multivariate predictive models play a crucial role in enhancing our understanding of complex biological systems and in developing innovative, replicable tools for translational medical research. However, the complexity of machine learning methods and extensive data pre-processing and feature engineering pipelines can lead to overfitting and poor generalizability. An unbiased evaluation of predictive models necessitates external validation, which involves testing the finalized model on independent data. Despite its importance, external validation is often neglected in practice due to the associated costs. Here we propose that, for maximal credibility, model discovery and external validation should be separated by the public disclosure (e.g. pre-registration) of feature processing steps and model weights. Furthermore, we introduce a novel approach to optimize the trade-off between efforts spent on model discoverytraining and external validation in such studies. We show on data involving more than 3000 participants from four different datasets that, for any "sample size budget", the proposed adaptive splitting approach can successfully identify the optimal time to stop model discovery so that predictive performance is maximized without risking a low powered, and thus inconclusive, external validation. The proposed design and splitting approach (implemented in the Python package "AdaptiveSplit") may contribute to addressing issues of replicability, effect size inflation and generalizability in predictive modeling studies.

Formatted: Highlight

Keywords: machine learning; predictive modelling; preregistration, external validation, adaptive splitting

Introduction

Multivariate predictive models integrate information across multiple variables to construct predictions of a specific outcome and hold promise for delivering more accurate estimates than traditional univariate methods (Woo *et al.*, 2017). For instance, in case of predicting individual behavioral and psychometric characteristics from brain data, such models can provide higher statistical power and better replicability, as compared to conventional mass-univariate analyses (Spisak *et al.*, 2023). Predictive models can utilize a variety of algorithms, ranging from simple linear regression-based models to complex deep neural networks. With increasing model complexity, the model will be more prone to overfit its training dataset, resulting in biased, overly optimistic in-sample estimates of predictive performance and often decreased generalizability to data not seen during model fit (Hosseini *et al.*, 2020). Internal validation approaches, like cross-validation (cv) provide means for an unbiased evaluation of predictive performance during model discovery by repeatedly holding out parts of the discovery dataset for testing purposes (Efron & Tibshirani, 1994; Poldrack *et al.*, 2020). However, internal validation approaches, in practice, still tend to yield overly optimistic performance estimates (Efron, 1983; Sui *et al.*, 2020; Varoquaux & Cheplygina, 2022). There are several reasons for this kind of effect size inflation. First, predictive modelling approaches typically display a high level of "analytical flexibility" and pose a large number of possible methodological choices in terms of feature pre-processing and model architecture, which emerge as uncontrolled (e.g. not cross-validated) "hyperparameters" during model discovery. Seemingly 'innocent' adjustments of such parameters can also lead to overfitting, if it happens outside the cv loop. The second reason for inflated internally validated performance estimates is 'leakage' of information from the test dataset to the training dataset (Kapoor & Narayanan, 2023). Information leakage has many faces. It can be a consequence of, for instance, feature standardization in a non cv-compliant way or, in medical imaging, the co-registration of brain data to a study-specific template. Therefore, it is often very hard to notice, especially in complex workflows. Another reason for overly optimistic internal validation results may be that even the highest quality discovery datasets can only yield an imperfect representation of the real world. Therefore, predictive models might capitalize on associations that are specific to the dataset at hand and simply fail to generalize "out-of-the-distribution", e.g. to different populations. Finally, some models might also be overly sensitive to unimportant characteristics of the training data, like subtle differences between batches of data acquisition or center-effects (Prosperi *et al.*, 2020; Spisak, 2022).

The obvious solution for these problems is *external validation*; that is, to evaluate the model's predictive performance on independent ('external') data that is guaranteed to be unseen during the whole model discovery procedure. There is a clear agreement in the community that external validation is critical for establishing machine learning model quality (Collins *et al.,* 2014; Ho *et al.,* 2020; Yu *et al.,* 2022; Spisak *et al.,* 2023; Poldrack *et al.,* 2020). However, the amount of data to be used for model discovery and external validation can have crucial implications on the predictive power, replicability and validity of predictive models and is, therefore, subject of intense discussion (Riley *et al.,* 2021; Marek *et al.,* 2022; Spisak *et al.,* 2023; Rosenberg & Finn, 2022; Thirion, 2023; Makowski *et al.,* 2023; Supplementary Table 1). Finding the optimal sample sizes is especially challenging for biomedical research, where this trade-off needs to weigh-in ethical and economic considerations. As a consequence, to date only around 10% of predictive modeling studies include an external validation of the model (Yang *et al.,* 2022). Those few studies performing true external validation often perform it on retrospective data (like Lee *et al.,* 2021 or Kincses *et al.,* 2024) or in separate, prospective studies (Spisak *et al.,* 2020; Kincses *et al.,* 2024). Both approaches can result in a suboptimal use of data and may slow down the dissemination process of new results.

In this manuscript we argue that maximal reliability and transparency during external validation can be achieved with prospective data acquisition preceded by "freezing" and publicly depositing (e.g. pre-registering) the whole feature processing workflow and all model weights. Furthermore, we present a novel adaptive design for predictive modeling studies with prospective data acquisition that optimizes the trade-off between efforts spent on model discovery ~~training~~ and external validation. We evaluate the proposed approach on data involving more than 3000 participants from four different datasets to illustrate that for any "sample size budget", it can successfully identify the optimal time to stop model discovery, so that predictive performance is maximized without risking a low powered, and thus inconclusive, external validation.

Background

The anatomy of a prospective predictive modelling study

Let us consider the following scenario: a research group plans to involve a fixed number of participants in a study with the aim of constructing a predictive model, and at the same time, evaluate its external validity. How many participants should they allocate for model discovery, and how many for external validation, to get the highest performing model as well as conclusive validation results?

In most cases it is very hard to make an educated guess about the optimal split of the total sample size into discovery and external validation samples prior to data acquisition. A possible approach is to use simplistic rules-of-thumb. Splitting data with an 80-20% ratio (a.k.a Pareto-split, Lipovetsky, 2009) is probably the most common method, but a 90-10% or a 50-50% may also be plausible choices (Raykar & Saha, 2015). However, as illustrated on Figure 1, such prefixed sample sizes are likely sub-optimal in many cases and the optimal strategy is actually determined by the dependence of the model performance on training sample size, that is, the “learning curve”. For instance, in case of a significant but generally low model performance (Figure 1A: flat learning curve) the model does not benefit a lot from adding more data to the discovery~~training~~ set but, on the other hand, it may require a larger external validation set for conclusive evaluation, due to the lower predictive effect size. This is visualized by the “power curve” on Figure 1, which shows the statistical power of external validation with the remaining samples as a function of sample size used for model discovery. The optimal strategy will be different, however, if the learning curve shows a persistent increase, without a strong saturation effect, meaning that predictive performance can be significantly enhanced by training the model on larger sample size (Figure 1B). In this case, the stronger predictive performance that can be achieved with larger training sample size, at the same time, allows a smaller external validation sample to be still conclusive. Finally, in some situations, model performance may rapidly get strong and reach a plateau at a relatively low sample size (Figure 1C). In such cases, the optimal strategy might be to stop early with the discovery phase and allocate resources for a more powerful external validation.

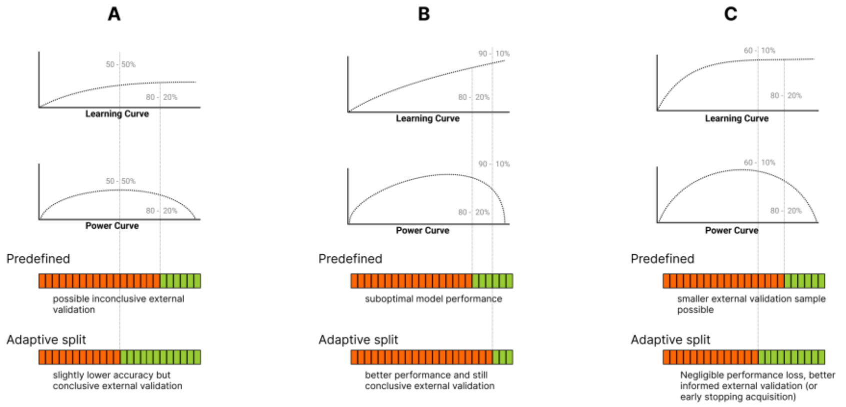

Figure 1: Examples of different optimal discovery and external validation sample sizes compared to a predefined 80-20% Pareto-split. (A) If the planned sample size and the model performance is low, the predefined external validation sample size might provide low statistical power to detect a significant model performance. **(B)** External validation of highly accurate models is well-powered; increasing the discovery~~training~~ sample size (against the external validation sample size) might result in a better performing final model. **(C)** Continuing training on the plateau of the learning curve will result in a negligible or biologically not relevant model performance improvement. In this case, a larger external validation sample (for more robust external performance estimates) or 'early stopping' of the data acquisition process might be desirable.

Transparent reporting of external validation: registered models

A key criterion for external validation is the independence of the external data from the data used during model discovery (Steyerberg & Harrell, 2016; Collins *et al.*, 2014; Spisak *et al.*, 2023). Regardless of the splitting strategy, an externally validated predictive modelling study must provide strong guarantees for this independence criterion. Pre-registration, i.e. the public disclosure of study plans before the start of the study, is an increasingly popular way of enhancing transparency and replicability in biomedical research (Nosek *et al.*, 2019; Spisak *et al.*, 2023) (Figure 2A), which could also be used to ensure the independence of the external validation sample.

However, as the concept of pre-registration was originally developed for confirmatory research, it does not fit well with the exploratory nature of the model discovery phase in typical predictive modelling endeavors. Specifically, while pre-registration necessitates that as many parameters of the analysis as possible are fixed before data acquisition, predictive modelling studies often involve a large number of hyperparameters (e.g. model architecture, feature pre-processing steps, regularization parameters, etc.) that are not known in advance and need to be optimized during the model discovery phase. This is especially true for complex machine learning models, like deep neural networks, where the number of free parameters can easily reach tens of thousands or even more. In such cases, the pre-registration of the discovery phase would require a large number of assumptions or simplifications, which would make the process ineffective and less transparent.

Therefore, we propose to perform the pre-registration after the model discovery phase, but before the external validation (Figure 2B). In this case, more freedom is granted for the discovery phase, while the external validation remains equally conclusive, as long as the pre-registration of the external validation includes all details of the *finalized* model (including the feature pre-processing workflow). This can easily be done by attaching the data and the reproducible analysis code used during the discovery phase or, alternatively, a serialized version of the fitted model (i.e. a file that contains all model weight). We refer to such models as **registered models**. While pre-registered external validation is, to date, sparse in the predictive modelling literature (Yang *et al.*, 2022), examples of studies using the proposed registered model design do exist, see e.g. Spisak *et al.*, 2020 or Kincses *et al.*, 2024. Such studies substantiate that the **registered model** approach allows model discovery with low sample sizes (n=39~~35~~ and n=25~~38~~ in the two studies, respectively) and still offer an unbiased evaluation of replicability and out-of-sample generalizability, without the need for data from thousands of individuals (as recently recommended by Marek, Tervo-Clemens et. al, 2022).

Formatted: Highlight

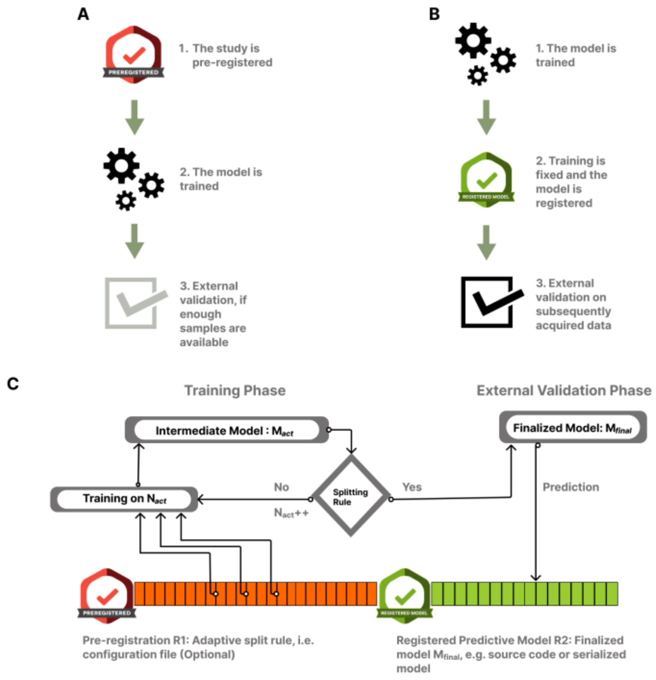

Figure 2: The registered model design and the proposed adaptive sample splitting procedure for prospective predictive modeling studies. (A) Predictive modelling combined with conventional pre-registration. In this case the pre-registration precedes data acquisition and requires fixing as many details of the analysis as possible. Given the potentially large number of coefficients to be optimized and the importance of hyperparameter optimization, conventional pre-registration exhibits a limited compatibility with predictive modelling studies. **(B)** Here we propose that in case of predictive modelling studies, public registration should only happen after the model is trained and finalized. The registration step in this case includes publicly depositing the finalized model, with all its parameters as well as all feature pre-processing steps. External validation is performed with the resulting *registered model*. This practice ensures a transparent, clear separation of model discovery and external validation. **(C)** The “registered model” design allows a flexible, adaptive splitting of the “sample size budget” into discovery and external validation phases. The proposed adaptive sample splitting procedure starts with fixing (and potentially pre-registering) a stopping rule (R1). During the discovery~~training~~ phase, one or more candidate models are trained and the splitting rule is repeatedly evaluated as the data acquisition proceeds. When the splitting rule “activates”, the model gets finalized (e.g. by being fit on the whole training sample) and publicly deposited/registered (R2). Finally, data acquisition continues and the prospective external validation is performed on the newly acquired data.

The adaptive splitting design

Even with registered models, the amount of data to be used for model discovery and external validation can have crucial implications on the predictive power, replicability and validity of predictive models. Here, we introduce a novel design for prospective predictive modeling studies that leverages the flexibility of model discovery granted by the registered model design. Our approach aims to adaptively determine an optimal splitting strategy during data acquisition. This strategy balances the model performance and the statistical power of the external validation (Figure 2C). The proposed design involves continuous model fitting and hyperparameter tuning throughout the discovery phase, for example, after every 10 new participants, and evaluating a 'stopping rule' to determine if the desired compromise between model performance and statistical power of the external validation has been achieved. This marks the end of the discovery phase and the start of the external validation phase, as well as the point at which the model must be publicly and transparently deposited or preregistered. Importantly, the preregistration should precede the continuation of data acquisition, i.e., the start of the external validation phase. In the present work, we propose and evaluate a concrete, customizable implementation for the splitting rule.

Methods and Implementation

Components of the stopping rule

The stopping rule of the proposed adaptive splitting design can be formalized as function S:

$$S_{\Phi}(X_{act}, y_{act}, \mathcal{M}) \qquad S: R^{\mathbb{2}} \longrightarrow \{True, False\} \tag{1}$$

where Φ denotes customizable parameters of the rule (detailed in the next paragraph), $X_{act} \in R^{\mathbb{2}}$ is the data (a matrix consisting of $n_{act} > 0$ observations and a fixed number of features p) and $y_{act} \in R$ is the prediction target, as acquired so far and $\mathcal{M}$ is the machine learning model to be trained. The discovery phase ends if and only if the stopping rule returns $True$.

Hard sample size thresholds

Our stopping rule is designed so that it can force a minimum size for both the discovery and the external validation samples, t_{min} and v_{min}, both being free parameters of the stopping rule.

Specifically:

$$\text{Min-rule:} \quad n_{act} \geq t_{min} \tag{2}$$

$$\text{Max-rule:} \quad n_{act} \geq n_{total} - v_{min} \tag{3}$$

where n_{act} and n_{total} are the actual sample size (e.g. participants measured so far) and the total sample size (i.e. the "sample size budget"), respectively, so that $n_{total} \geq n_{act} > 0$. Setting t_{min} and v_{min} may be useful to prevent early stopping at the beginning of the training procedure, where predictive performance and validation power estimates are not yet reliable due to the small n_{act} or to ensure that a minimal validation sample size, even if stopping criteria are never met. If t_{min} and v_{min} are set so that $t_{min} + v_{min} = n_{total}$ then our approach falls back to training a registered model with predefined discovery~~training~~ and validation sample sizes.

Forecasting Predictive Performance via Learning Curve Analysis

Taking internally validated performance estimates of the candidate model as a function of training sample size, also known as learning curve analysis, is a widely used approach to gain deeper insights into model discovery~~training~~ dynamics (see examples on Figure 1). In the proposed stopping rule, we will rely on learning curve analysis to provide estimates of the current predictive performance and the expected gain when adding new data to the discovery sample.

Performance estimates can be unreliable or noisy in many cases, for instance with low sample sizes or when using leave-one-out cross-validation (Varoquaux, 2018). To obtain stable and reliable learning curves, we propose to

calculate multiple cross-validated performance estimates from sub-samples sampled without replacement from the actual data set. The proposed procedure is detailed in Algorithm 1.

Algorithm 1 (Bootstrapped Learning Curve Analysis)

1. **Require** $\mathbf{X}_{act}, \mathbf{y}_{act}, \mathcal{M}$
2. **Set** $n_b \leftarrow$ `<number of bootstrap iterations>`
3. **For** $t \leftarrow 1$ to n_{act} *(loop over sample sizes)*
    4. **For** $i \leftarrow 1$ to n_b *(bootstrap iterations)*
        5. **Set** $\mathbf{b} \leftarrow$ sample t indices from $< 1, \ldots, n_{act} >$ without replacement
        6. **Set** $\mathbf{X}_b \leftarrow \mathbf{X}_{act}[\mathbf{b}]$
        7. **Set** $\mathbf{y}_b \leftarrow \mathbf{y}_{act}[\mathbf{b}]$
        8. **Set** $\mathbf{s}[i] \leftarrow$ cross-validated performance score of $\mathcal{M}$ fitted to $(\mathbf{y}_b, \mathbf{X}_b)$
    5. **End For**
    6. **Set** $\mathbf{l}_{act}[t] \leftarrow median(\mathbf{s})$
4. **End For**
5. **Return** $\mathbf{l}_{act}$ *(bootstrapped learning curve)*

The learning curve analysis allows the discovery phase to be stopped if the expected gain in predictive performance is lower than a predefined relevance threshold and can be used for instance for stopping model training earlier in well-powered experiments and retain more data for the external validation phase. Specifically, the stopping rule S will return $True$ if the *Min-rule* (Eq. 2) is $True$ or the following is true:

$$\text{Performance-rule:} \quad \widehat{s_{total}} - s_{act} \leq s_{min} \qquad (4)$$

where s_{act} is the actual bootstrapped predictive performance score (i.e. the last element of l_{act}, as returned by Algorithm 1, $\widehat{s_{total}}$ is a estimate of the (unknown) predictive performance s_{total} (i.e. the predictive performance of the model trained on the whole sample size) and s_{min} ~~ϵ_{s}~~ is the smallest predictive effect of interest. Note that

this parameter configuration essentially switches off the performance rule for our main analysis ($s_{min} = 0$, but see Supplementary material, figure 7, for an analysis of the effect of the performance rule) and ensures that even in case of very small simulated sample size budgets, the training sample is suitable for cross-validation ($v\_min =$ 12).

~~setting $\epsilon_s = -\infty$ deactivates the *Performance-rule* (Eq. 4).~~

While s_{total} is typically unknown at the time of evaluating the stopping rule S, there are various approaches of obtaining an estimate $\widehat{s_{total}}$. In the base implementation of AdaptiveSplit, we stick to a simple method: we extrapolate the learning curve l_{act} based on its tangent line at n_{act}, i.e. assuming that the latest growth rate will remain constant for the remaining samples. While in most scenarios this is an overly optimistic estimate, it still provides a useful upper bound for the maximally achievable predictive performance with the given sample size and can successfully detect if the learning curve has already reached a flat plateau (like on Figure 1C).

Statistical power of the external validation sample

Even if the learning curve did not reach a plateau, we still need to make sure that we stop the discovery~~training~~ phase early enough to save a sufficient amount of data for a successful external validation from our sample size budget. Given the actual predictive performance estimate s_{act} and the size of the remaining, to-be-acquired sample $s_{total} - s_{act}$, we can estimate the probability that the external validation correctly rejects the null hypothesis (i.e. zero predictive performance). This type of analysis, known as power calculation, allows us to determine the optimal stopping point that guarantees the desired statistical power during the external validation. Specifically, the stopping rule S will return $True$ if the *Performance-rule* (Eq. 4) is $False$ and the following is true:

$$\text{Power-rule:} \quad POW_{\alpha}(s_{act}, n_{val}) \leq v_{pow} \tag{5}$$

where $POW_{\alpha}(s, n)$ is the power of a validation sample of size n to detect an effect size of s and $n_{val} = n_{total} - n_{act}$ is the size of the validation sample if stopping, i.e. the number of remaining (not yet measured) participants in the experiment. Given that machine learning model predictions are often non-normally distributed (Spisak,

2022), our implementation is based on a bootstrapped power analysis for permutation tests, as shown in Algorithm 2. Our implementation is, however, simple to extend with other parametric or non-parametric power calculation techniques.

Algorithm 2 (Calculation of the Power-rule)

1\. **Require** $\mathbf{X}_{act}, \mathbf{y}_{act}, n_{validation}, \mathcal{M}, \alpha$
2\. **Set** $n_b \leftarrow$ `<number of bootstrap iterations>`
3\. **Set** $n_\pi \leftarrow$ `<number of permutations>`
4\. **Set** $\hat{\mathbf{y}}_{act} \leftarrow$ cross-validated prediction from $\mathbf{X}_{act}$ with $\mathcal{M}$
    5\. **For** $i \leftarrow 1$ to n_b
        6\. **Set** $\mathbf{b} \leftarrow$ sample t indices from $< 1, \ldots, n_{val} >$ with replacement
        7\. **Set** $\mathbf{y}_b \leftarrow \mathbf{y}_{act}[\mathbf{b}]$
        8\. **Set** $\hat{\mathbf{y}}_b \leftarrow \hat{\mathbf{y}}_{act}[\mathbf{b}]$
        9\. **Set** $r_{obs} = correlation(\mathbf{y}_b, \hat{\mathbf{y}}_b)$
        10\. **For** $j \leftarrow 1$ to n_p
            11\. **Set** $\boldsymbol{\pi} \leftarrow$ permute$(< 1, \ldots, n_{val} >)$
            12\. **Set** $\mathbf{y}_\pi \leftarrow \mathbf{y}_b[\boldsymbol{\pi}]$
            13\. **Set** $\hat{\mathbf{y}}_\pi \leftarrow \hat{\mathbf{y}}_b[\boldsymbol{\pi}]$
            14\. **Set** $\mathbf{r}_{null}[j] = correlation(\mathbf{y}_\pi, \hat{\mathbf{y}}_\pi)$
        11\. **End For**
        12\. **Set** $\mathbf{p}[i] \leftarrow \#(\mathbf{r}_{null} > r_{obs})/n_{perm}$
    6\. **End For**
5\. **Set** $power = \#(\mathbf{p} < \alpha)/n_b$
6\. **Return** $power$

Note that depending on the aim of external validation, the *Power-rule* can be swapped to, or extended with, other conditions. For instance, if we are interested in accurately estimating the predictive effect size, we could condition the stopping rule on the width of the confidence interval for the prediction performance.

Calculating the validation power (Algorithm 2) for all available sample sizes ($n = 1 \ldots n_{act}$) defines the so-called "validation power curve" (see Figure 1 and Supplementary Figures 2, 4 and 6), that represents the expected ratio of true positive statistical tests on increasing sample size calculated on the external validation set. Various extrapolations of the power curve can predict the expected stopping point during the course of the experiment.

Stopping Rule

Our proposed stopping rule integrates the Min-rule, the Max-rule, the Performance-rule and the Power-rule in the following way:

$$S_{\Phi}(X_{act}, y_{act}, \mathcal{M}) = \text{Min-rule} \quad AND \\ (\\ \text{Max-rule} \quad OR \\ \text{Performance-rule} \quad OR \\ \text{Power-rule} \\ ) \tag{6}$$

where $\Phi =< t_{min}, v_{min}, s_{min}, v_{pow}, \alpha >$ are parameters of the stopping rule: minimum training sample size, minimum validation sample size, minimum effect of interest and target power for the external validation and the significance threshold, respectively.

We have implemented the proposed stopping rule in the Python package "*adaptivesplit*" (Gallitto et al., n.d.). The package can be used together with a wide variety of machine learning tools and provides an easy-to-use interface to work with scikit-learn (Pedregosa *et al.*, 2012) models.

Empirical evaluation

We evaluate the proposed stopping rule, as implemented in the package *adaptivesplit* (Gallitto et al., n.d.), in four publicly available datasets; the Autism Brain Imaging Data Exchange (ABIDE; Di Martino *et al.*, 2013), the Human Connectome Project (HCP; Van Essen *et al.*, 2013), the Information eXtraction from Images (IXI; Hill *et al.*, 2005) and the Breast Cancer Wisconsin (BCW; Street *et al.*, 1993) datasets (Fig. 3).

ABIDE

We obtained preprocessed data from Autism Brain Imaging Data Exchange (ABIDE) dataset (Di Martino *et al.*, 2013) involving the resting-state data of 866 participants (Autism Spectrum Disorder: 402, neurotypical control: 464). Pre-processed regional time-series data were obtained as shared by Dadi *et al.*, 2019, which were based on image data provided by the Pre-processed Connectome Project (Craddock *et al.*, 2013a), pre-processed using the C-PAC pipeline (Craddock et al., 2013b; Preprocessed Connectomes Project, n.d.), without global signal regression. Tangent correlation across the time series of the n=122 regions of the BASC brain parcellation (Multi-level bootstrap analysis of stable clusters; Bellec *et al.*, 2010) was computed with nilearn (Abraham et al., 2014). The resulting functional connectivity estimates were considered features for a predictive model of autism diagnosis.

HCP

The Human Connectome Project dataset contains imaging and behavioral data of approximately 1,200 healthy subjects (Van Essen *et al.*, 2013). Pre-processed resting state functional magnetic resonance imaging (fMRI) connectivity data (partial correlation of the mean regional timeseries of 100 brain parcels derived via independent component analysis; Glasser *et al.*, 2013 as published with the HCP1200 release (N=999 participants with functional connectivity data) were used to build models that predict individual fluid intelligence scores (Gf), measured with Penn Progressive Matrices (Duncan *et al.*, 2000). The minimal preprocessing pipelines of Glasser et al. for structural, functional, and diffusion MRI that were developed by the HCP and included spatial artifact/distortion removal, surface generation, cross-modal registration, and alignment to standard space. These pipelines were specially designed to capitalize on the high-quality data offered by the HCP.

IXI

The IXI dataset is published by the Neuroimage Analysis Center, from Imperial College London, in the United Kingdom, and it is part of the project Brain Development. It consists of approximately 600 structural MRI images from a diverse population of healthy individuals, including both males and females across a wide age range. The dataset contains high-resolution brain images from three different MRI scanners (Philips Intera 3T, Philips Gyroscan Intera 1.5T and GE 1.5T) and associated demographic information, making it suitable for studying age-

related changes in brain structure and function. Structural pre-processing of T1-weighted images was conducted using FreeSurfer (Fischl, 2012) software (version 6.0), run with default parameters, focusing on grey matter volume. The procedure included motion correction, skull stripping, removal of the cerebellum and brain stem, intensity correction, segmentation, tessellation, smoothing and topology correction (Kotikalapudi, 2024). Cortical volume of brain regions was measured using the Desikan-Killiany brain atlas (Desikan et al., 2006), producing 68 regional volume measures (34 per hemisphere, measured in mm^3).

BCW

The Breast Cancer Wisconsin (BCW, Street et al., 1993) dataset contains diagnostic features computed from digitized images of fine needle aspirates (FNA) of breast masses. The FNA procedure involves using a thin, hollow needle to extract cells from a suspicious area of breast tissue. These cells are then smeared onto glass slides, stained to highlight cellular structures, and scanned to create digital images. Specialized software analyses these images (Wolberg et al., 1994) to extract 30 different features, which quantify various morphological characteristics of the cell nuclei, such as size, shape, and texture. These features are used to create a predictive model for breast cancer diagnosis, with the target variable being the diagnosis categorized as malignant (M) or benign (B).

The chosen datasets include both classification and regression tasks and span a wide range in terms of number of participants, number of predictive features, achievable predictive effect size and data homogeneity (see Supplementary Figures 1-6). Our analyses aimed to contrast the proposed adaptive splitting method with the application of fixed training and validation sample sizes, specifically using 50, 60 or 90% of the total sample size for ~~training~~ discovery and the rest for external validation. We simulated various “sample size budgets” (total sample sizes, n_{total}) with random sampling without replacement. For a given total sample size, we simulated the prospective data acquisition procedure by incrementing n_{act}; starting with 10% of the total sample size and going up with increments of five. In each step, the stopping rule was evaluated with “AdaptiveSplit”, fitting a Ridge model (for regression tasks; HCP and IXI datasets) or a L2-regularized logistic regression (for classification tasks; ABIDE and BCW datasets). Model fit always consisted of a cross-validated fine-tuning of the α regularization parameter ($\alpha \in \{0.1, 1, 10\}$), resulting in a nested cv estimate of prediction performance and validation power. Robust estimates (and confidence intervals) were obtained with bootstrapping, as described in Algorithm 1 and

Algorithm 2. This procedure was iterated until the stopping rule returned True. The corresponding sample size was then considered the final training discovery sample. With all four splitting approaches (adaptive, Pareto, Half-split, 90-10% split), we trained the previously described Ridge or regularized logistic regression model on the training discovery sample and obtained predictions for the sample left out for external validation. This whole procedure was repeated 100 times for each simulated sample size budget in each dataset, to estimate the confidence intervals for the models performance in the external validation and its statistical significance. In all analyses, the adaptive splitting procedure is performed with a target power of $v_{pow} = 0.8$, an $alpha = 0.05$, $t_{tmin} = n_{total}/3$, $v_{min} = 12$, $s_{min} = 0 - \infty$. P-values were calculated using a permutation test with 5000 permutations.

Results

The results of our empirical analyses of four large, openly available datasets confirmed that the proposed adaptive splitting approach can successfully identify the optimal time to stop acquiring data for training and maintain a good compromise between maximizing both predictive performance and external validation power with any sample size budget.

In all four samples, the applied models yielded a statistically significant predictive performance at much lower sample sizes than the total size of the dataset, i.e. all datasets were well powered for the analysis. Thus, when reporting our results, we focused on the most realistic scenarios and omitted sample size budgets that were powered too low (neither of the splitting strategies leads to significant model performance) or too high (prediction performance plateaus with all splitting strategies) for any meaningful comparison between splitting strategies. Trained on the full sample size with cross-validation, the models displayed the following performances: functional brain connectivity from the HCP dataset explained 13% of the variance in cognitive abilities; structural MRI data (gray matter probability maps) in the IXI dataset explained 48% in age; classification accuracy was 65.5% for autism diagnosis (functional brain connectivity) in the ABIDE dataset and 92% for breast cancer diagnosis in the BCW dataset.

Formatted: Highlight

Formatted: Highlight

The datasets varied not only in the achievable predictive performance but also in the shape of the learning curve, with different sample sizes and thus, they provided a good opportunity to evaluate the performance of our stopping rule in various circumstances (Supplementary Figures 1-6).

We found that adaptively splitting the data provided external validation performances that were comparable to the commonly used Pareto split (80-20%) in most cases (Figure 3, left column). From the fixed splitting approaches, the half-split assigns the least samples from the total sample size budget to the training phase (50%). Thus, the resulting model is trained on less data than with other strategies, typically resulting in a smaller l_{act}. While this lower effect size should in general result in lower statistical power during the external validation phase, the half-split approach can counterbalance this with the larger sample size remaining for external validation. Our analysis shows, that this happens in almost all of the cases, hinting that in research scenarios where the expected predictive performance is low, researchers should either use the proposed adaptive splitting procedure, or aim for a relatively large pre-fixed external validation sample~~As expected, half-split tended to provide worse predictive performance due to the smaller training sample~~. In contrast, 90-10% tended to display only slightly higher performances than the Pareto and the Adaptive splitting techniques, in most cases. This small achievement came with a big cost in terms of the statistical power in the external validation sample, where the 90-10% split very often gave inconclusive results ($p \geq 0.05$) (Figure 3, right column), especially with low sample size budgets. Although to a lesser degree, Pareto split also frequently failed to yield a conclusive external validation with small total sample sizes. In addition to the Pareto, half-split, and 90-10% splitting strategies, we also evaluated alternative split ratios (75-25% and 70-30%), which are commonly used in the literature. The 75-25% split demonstrated performance comparable to the Pareto and adaptive splitting techniques, although, similarly to Pareto, it struggled to achieve statistical significance at smaller sample sizes. In contrast, the 70-30% split exhibited good statistical significance at the cost of lower overall performance, comparable to the trend observed with the half-split strategy (see Supplementary material, Figure 13). Adaptive splitting (as well as half-split) provided sufficient statistical power for the external validation in most cases. This was achieved by applying different strategies in different scenarios. In case of low total sample sizes, it retained a larger proportion of the sample for the external validation phase in order to achieve sufficient power, up to using 79% of the data for external validation. On the other hand, if the total sample size budget allowed it, adaptive splitting let the predictive model benefit from larger training samples, retaining ~~as low as~~ 8% or less of the data for external validation in~~s~~ such cases.

Focusing only on cases with a successful, conclusive external validation, the proposed adaptive splitting strategy provided an external validation performance comparable to the alternative fixed splitting strategies, in all cases where the external validation was conclusive (statistically significant). Furthermore, in contrast to the investigated fixed splitting strategies, the proposed splitting strategy yields solid guarantees for the success of the external validation phase, independent of the sample size budget.~~proposed adaptive splitting strategy always provided equally good or better predictive performance than the fixed splitting strategies (as shown by the 95% confidence intervals on Figure 3).~~

Commented [GG1]: Fix figure 3, one of the plots is cropped

Formatted: Suppress line numbers

**Classification**	**BCW**					**ABIDE**				
Sample sizes	49	65	86	113	150	400	442	489	542	599
Adaptive Splits	21-79	33-67	48-52	67-33	92-08	41-59	49-51	59-41	71-29	82-18
Discovery Scores	0.888	0.921	0.933	0.938	0.944	0.614	0.624	0.633	0.640	0.644
External Validation scores	0.896	0.927	0.935	0.941	0.944	0.626	0.634	0.634	0.643	0.655
Statistical Significance	0.036	0.032	0.033	0.024	0.041	0.018	0.014	0.023	0.027	0.017
**Regression**	**HCP**					**IXI**				
Sample sizes	242	272	305	343	384	49	65	86	113	150
Splits	44-56	53-47	64-36	76-24	89-11	21-79	25-75	40-60	61-39	89-11
Discovery Scores	-15.35	-15.20	-15.10	-15.02	-14.89	-12.01	-11.93	-11.72	-11.54	-11.16
External Validation scores	-15.24	-15.13	-15.09	-14.82	-14.82	-11.95	-12.08	-11.40	-11.12	-10.74
Statistical Significance	0.012	0.016	0.019	0.033	0.029	0.070	0.031	0.014	0.021	0.041

Table 1: Performance results of the "adaptivesplit" algorithm for each dataset across the different sample sizes (n_{act}). The fraction of discovery samples (orange) and external validation samples (green) is shown for each split. For each n_{act}, the relative accuracy (for classification tasks) or negative mean absolute error (for regression tasks) is reported, along with statistical significance (p-value), providing a comprehensive overview of the algorithm's performance across different datasets and sample sizes.

Formatted: Font: 9 pt, English (United States)

Formatted: Font: 9 pt

Formatted: Font: 9 pt, English (United States)

Formatted: Font: 9 pt, Not Italic, English (United

Formatted: Font: 9 pt, Not Italic, English (United

Formatted: Font: 9 pt, Not Italic, English (United

Formatted: Font: 9 pt, Not Italic, English (United

Formatted: Font: 9 pt

Formatted: Suppress line numbers

Formatted: Indent: Left: 0.5", Suppress line numbers

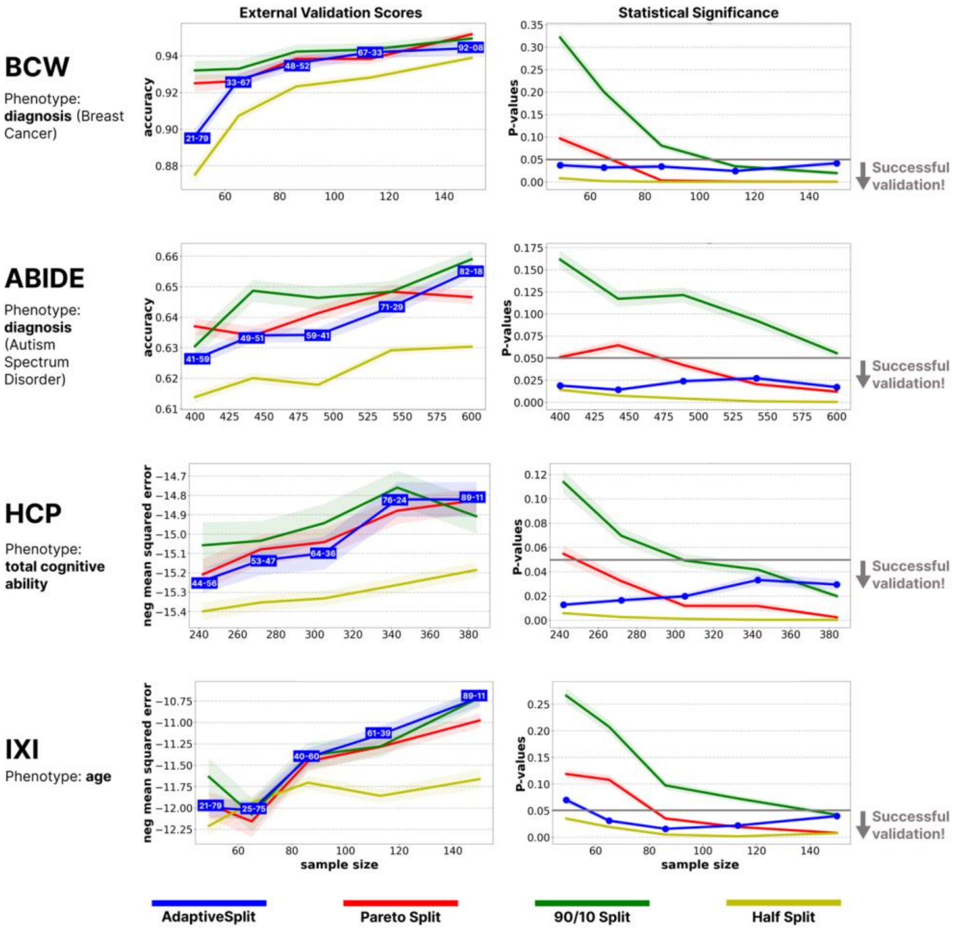

Figure 3: The proposed adaptive splitting approach provides a good compromise between predictive performance and statistical power of the external validation. The left and right column shows the comparison of splitting methods on external validation performance and p-values, respectively, at various n_{total}. Confidence intervals are based on 100 repetitions of the analyses. The adaptive splitting approach (blue) provides a good compromise between predictive performance and statistical power of the external validation. The Pareto split (redorange) provides similar external validation performances to adaptive splitting; however it

often fails to provide conclusive results due to an insufficient sample size during external validation, especially in case of a limited sample size budget. The 90-10% split (green) provides only slightly higher performances than the Pareto and the Adaptive splitting techniques, but it very often gives inconclusive results ($p \geq 0.05$) in the external validation sample. Half-split (yellowred) tends to provide worse predictive performance due to the too small discoverytraining sample.

Formatted: Suppress line numbers

Discussion

Here we have proposed "registered models", a novel design for prospective predictive modeling studies that allows flexible model discovery and trustworthy prospective external validation by fixing and publicly depositing the model after the discovery phase. Furthermore, capitalizing on the flexibility during model discovery with the registered model design, we have proposed a stopping rule for adaptively splitting the sample size budget into discovery and external validation phases. These approaches together provide a robust and flexible framework for predictive modeling studies and address several common issues in the field, including overfitting, effect size inflation as well as the lack of reliability and reproducibility.

Registered models provide a clear and transparent separation between the discovery and external validation phases, which is essential for ensuring the independence of the external validation data. Thereby, they provide a straightforward solution to several of the widely discussed issues and pitfalls of predictive model development (Efron, 1983; Sui *et al.*, 2020; Varoquaux & Cheplygina, 2022; Marek *et al.*, 2022; Spisak *et al.*, 2023). With registered models, external validation estimates are guaranteed to be free of information leakage (Kapoor & Narayanan, 2023) and provide an unbiased estimate of the model's predictive performance.

With registered models, the question of how the total sample size budget should be distributed between the discovery and external validation phase remains of central importance for the optimal use of available resources (scanning time, budget, limitations in participant recruitment) (Archer *et al.*, 2020; Riley *et al.*, 2021; Marek *et al.*, 2022; Spisak *et al.*, 2023; Rosenberg & Finn, 2022; Thirion, 2023; Makowski *et al.*, 2023; Supplementary Table 1). Optimal sample sizes are often challenging to determine prior to the study. The proposed adaptive splitting procedure promises to provide a solution in such cases by allowing the sample size to be adjusted during the data acquisition process, based on the observed performance of the model trained on the already available data. We performed a thorough evaluation of the proposed adaptive splitting procedure on data from more than

3000 participants from four publicly available datasets. We found that the proposed adaptive splitting approach can successfully identify the optimal time to stop acquiring data for training and maintain a good compromise between maximizing both predictive performance and external validation power with any “sample size budget”. When contrasting splitting approaches based on fixed validation size with the proposed adaptive splitting technique, using the latter was always the preferable strategy to maximize power and statistical significance during external validation. The benefit of adaptively splitting the data acquisition for training and validation provides the largest benefit in lower sample size regimes. In case of larger total sample size budgets, the fixed Pareto split (20-80%) provided also good results, giving similar external validation performances to adaptive splitting, without having to repeatedly re-train the model during data acquisition. Thus, for moderate to large sample sizes and well powered models, the Pareto split might be a good alternative to the adaptive splitting approach, especially if the computational resources for re-training the model are limited.

Of note, the presented implementation of adaptive data splitting aims to maximize the discovery~~training~~ sample (and minimize the external validation sample) in order to achieve the highest possible performance together with a conclusive (statistically significant) external validation. However, the resulting external performance estimates will still be subject of sampling variance. If the aim is to provide more reliable estimates of the predictive effect size in the external validation, the power-rule in the proposed approach can be modified so that it stops the discovery phase when a desired confidence interval width for the external effect size estimate is reached.

The proposed adaptive splitting design can advance the development of predictive models in several ways. Firstly, it provides a simple way to perform both model discovery and initial external validation in a single study. Furthermore, it promotes the public deposition (registration) of models at an early stage of the study, enhancing transparency, reliability and replicability. Finally, it provides a flexible approach to data splitting, which can be adjusted according to the specific needs of the study.

In conclusion, registered models provide a simple approach to guarantee the independence of model discovery and external validation and for the development and initial evaluation of registered models with unknown power, the introduced adaptive splitting procedure provides a robust and flexible approach to determine the optimal ratio of data to be used for model discovery and external validation. Together, registered models and the adaptive splitting procedure, address several common issues in the field, including overfitting, cross-validation failure, and boost the reliability and reproducibility.

Data and Code availability

Empirical analysis was based on data provided by the following sources: (i) the Human Connectome Project (WU-Minn Consortium, principal investigators: D. Van Essen and K. Ugurbil; 1U54MH091657), funded by the 16 National Institutes of Health (NIH) institutes and centers that support the NIH Blueprint for Neuroscience Research, (ii) the ABIDE consortium (Di Martino *et al.*, 2013), (iii) the Imperial college London (IXI, principal investigator: Hill D.L., other investigators: Williams S.C.R., Smith S.M., Hawkes, D; GR/S21533/02) and (iv) the University of Wisconsin (Street et al., 1993). Raw and pre-processed data used in the present study are publicly available for download in their respective repositories:

- ABIDE raw data (Di Martino *et al.*, 2013; available at https://fcon_1000.projects.nitrc.org/indi/abide/):
- ABIDE preprocessed dataset (Dadi et al., 2019; available at https://osf.io/hc4md)
- HCP1200 raw data (Van Essen et al., 2013; available at https://db.humanconnectome.org/)
- HCP1200 preprocessed data (Glasser *et al.,* 2013; available at https://www.humanconnectome.org/)
- BCW preprocessed dataset (Street *et al.,* 1993; available at https://www.kaggle.com/datasets/uciml/breast-cancer-wisconsin-data).
- IXI raw data (Hill et al., 2005; https://brain-development.org/ixi-dataset/).
- IXI preprocessed dataset (Kotikalapudi, 2024; available at https://zenodo.org/records/11635168).

The Python implementation of the "adaptivesplit" package is publicly available on GitHub (Gallitto et al., n.d.; https://github.com/pni-lab/adaptivesplit). Additionally, the Python scripts and data used for the analyses presented in this manuscript can be accessed in the following GitHub repository: https://github.com/pni-lab/AdaptiveSplitAnalysis.

~~*The Python implementation of the "adaptivesplit" package is available on GitHub (Gallitto et al., n.d.).*~~

Formatted: Normal, No bullets or numbering, Suppress line numbers

Formatted: Suppress line numbers

Availability of supporting code and requirements

Project name: adaptivesplit

Formatted: Don't suppress line numbers

Project home page: https://github.com/pni-lab/adaptivesplit

Operating system(s): Platform independent

Programming language: Python

Other requirements: Python 3.9 or higher

License: GNU General public licence, version 3, 29 June 2007 (GPL-3.0)

RRID: SCR_025888

bio.tools: bio.tools:adaptivesplit

Formatted: Suppress line numbers

~~The Python implementation of the "adaptivesplit" package is available on GitHub (Gallitto et al., n.d.).~~

Acknowledgements

The work is funded by the Deutsche Forschungsgemeinschaft (DFG, German Research Foundation) - Project-ID 422744262 - TRR 289 (Gefördert durch die Deutsche Forschungsgemeinschaft (DFG) – Projektnummer 422744262 – TRR 289).

Formatted: English (United Kingdom)

Competing interests

The authors declare that they have no competing interests.

References

Abraham, A., Pedregosa, F., Eickenberg, M., Gervais, P., Mueller, A., Kossaifi, J., ... & Varoquaux, G. (2014). Machine learning for neuroimaging with scikit-learn. Frontiers in neuroinformatics, 8, 14.

Archer, L., Snell, K. I. E., Ensor, J., Hudda, M. T., Collins, G. S., & Riley, R. D. (2020). Minimum sample size for external validation of a clinical prediction model with a continuous outcome. *Statistics in Medicine*, *40*(1), 133–146. 10.1002/sim.8766

Bellec, P., Rosa-Neto, P., Lyttelton, O. C., Benali, H., & Evans, A. C. (2010). Multi-level bootstrap analysis of stable clusters in resting-state fMRI. *NeuroImage*, *51*(3), 1126–1139. 10.1016/j.neuroimage.2010.02.082

Collins, G. S., de Groot, J. A., Dutton, S., Omar, O., Shanyinde, M., Tajar, A., Voysey, M., Wharton, R., Yu, L.-M., Moons, K. G., & Altman, D. G. (2014). External validation of multivariable prediction models: a systematic review of methodological conduct and reporting. *BMC Medical Research Methodology*, *14*(1). 10.1186/1471-2288-14-40

Field Code Changed

Craddock, C., Benhajali, Y., Chu, C., Chouinard, F., Evans, A., Jakab, A., & Bellec, P. (2013a). The neuro bureau preprocessing initiative: open sharing of preprocessed neuroimaging data and derivatives. Frontiers in Neuroinformatics, 7(27), 5. 10.3389/conf.fninf.2013.09.00041

Craddock, C., Sikka, S., Cheung, B., Khanuja, R., Ghosh, S. S., Yan, C., … & Milham, M. (2013b). Towards automated analysis of connectomes: The configurable pipeline for the analysis of connectomes (C-PAC). Frontiers in Neuroinformatics, 42 (10.3389).

Dadi, K., Rahim, M., Abraham, A., Chyzhyk, D., Milham, M., Thirion, B., & Varoquaux, G. (2019). Benchmarking functional connectome-based predictive models for resting-state fMRI. *NeuroImage*, *192*, 115–134. 10.1016/j.neuroimage.2019.02.062

Desikan, R. S., Ségonne, F., Fischl, B., Quinn, B. T., Dickerson, B. C., Blacker, D., ... & Killiany, R. J. (2006). An automated labeling system for subdividing the human cerebral cortex on MRI scans into gyral based regions of interest. Neuroimage, 31(3), 968-980.

Di Martino, A., Yan, C.-G., Li, Q., Denio, E., Castellanos, F. X., Alaerts, K., Anderson, J. S., Assaf, M., Bookheimer, S. Y., Dapretto, M., Deen, B., Delmonte, S., Dinstein, I., Ertl-Wagner, B., Fair, D. A., Gallagher, L., Kennedy, D. P., Keown, C. L., Keysers, C., … Milham, M. P. (2013). The autism brain imaging data exchange: towards a large-scale evaluation of the intrinsic brain architecture in autism. *Molecular Psychiatry*, *19*(6), 659–667. 10.1038/mp.2013.78

Duncan, J., Seitz, R. J., Kolodny, J., Bor, D., Herzog, H., Ahmed, A., Newell, F. N., & Emslie, H. (2000). A Neural Basis for General Intelligence. *Science*, *289*(5478), 457–460. 10.1126/science.289.5478.457

Efron, B. (1983). Estimating the Error Rate of a Prediction Rule: Improvement on Cross-Validation. *Journal of the American Statistical Association*, *78*(382), 316–331. 10.1080/01621459.1983.10477973

Efron, B., & Tibshirani, R. J. (1994). *An Introduction to the Bootstrap*. Chapman. 10.1201/9780429246593

Fischl, B. (2012). FreeSurfer. *NeuroImage*, *62*(2), 774–781. 10.1016/j.neuroimage.2012.01.021

Gallitto, G., Englert, R., Kincses, B., Kotikalapudi, R., Li, J., Hoffschlag, K., Bingel, U., Spisak, T. (n.d.). *adaptivesplit* [Computer software]. GitHub. Retrieved May 27, 2024 from https://github.com/pni-lab/adaptivesplit

Field Code Changed

Field Code Changed

Field Code Changed

Glasser, M. F., Sotiropoulos, S. N., Wilson, J. A., Coalson, T. S., Fischl, B., Andersson, J. L., Xu, J., Jbabdi, S., Webster, M., Polimeni, J. R., Van Essen, D. C., & Jenkinson, M. (2013). The minimal preprocessing pipelines for the Human Connectome Project. *NeuroImage*, *80*, 105–124. 10.1016/j.neuroimage.2013.04.127

Hill, D.L., Williams, S.C.R., Smith, S.M, Hawkes, D. (2005). Information eXtraction from Images (IXI). Available at http://brain-development.org/ixi-dataset/

Ho, S. Y., Phua, K., Wong, L., & Bin Goh, W. W. (2020). Extensions of the External Validation for Checking Learned Model Interpretability and Generalizability. *Patterns*, *1*(8), 100129. 10.1016/j.patter.2020.100129

Hosseini, M., Powell, M., Collins, J., Callahan-Flintoft, C., Jones, W., Bowman, H., & Wyble, B. (2020). I tried a bunch of things: The dangers of unexpected overfitting in classification of brain data. *Neuroscience & Biobehavioral Reviews*, *119*, 456–467. 10.1016/j.neubiorev.2020.09.036

Kapoor, S., & Narayanan, A. (2023). Leakage and the reproducibility crisis in machine-learning-based science. *Patterns*, *4*(9), 100804. 10.1016/j.patter.2023.100804

Kincses, B., Forkmann, K., Schlitt, F., Pawlik, R., Schmidt, K., Timmann, D., Elsenbruch, S., Wiech, K., Bingel, U., & Spisak, T. (2024). *An externally validated resting-state brain connectivity signature of pain-related learning*. Accepted in Communications Biology, Preprint: 10.31219/osf.io/utkbv

Kotikalapudi, R. (2024). IXI – Information eXtraction from Images | Cortical Volume [Data set]. Zenodo. https://doi.org/10.5281/zenodo.11635168

Lee, J.-J., Kim, H. J., Čeko, M., Park, B., Lee, S. A., Park, H., Roy, M., Kim, S.-G., Wager, T. D., & Woo, C.-W. (2021). A neuroimaging biomarker for sustained experimental and clinical pain. *Nature Medicine*, *27*(1), 174–182. 10.1038/s41591-020-1142-7

Lipovetsky, S. (2009). Pareto 80/20 law: derivation via random partitioning. *International Journal of Mathematical Education in Science and Technology*, *40*(2), 271–277. 10.1080/00207390802213609

Makowski, C., Brown, T. T., Zhao, W., Hagler, D. J., Parekh, P., Garavan, H., Nichols, T. E., Jernigan, T. L., & Dale, A. M. (2023). *Leveraging the Adolescent Brain Cognitive Development Study to improve behavioral prediction from neuroimaging in smaller replication samples*. 10.1101/2023.06.16.545340

Field Code Changed

Marek, S., Tervo-Clemmens, B., Calabro, F. J., Montez, D. F., Kay, B. P., Hatoum, A. S., Donohue, M. R., Foran, W., Miller, R. L., Hendrickson, T. J., Malone, S. M., Kandala, S., Feczko, E., Miranda-Dominguez, O., Graham, A. M., Earl, E. A., Perrone, A. J., Cordova, M., Doyle, O., … Dosenbach, N. U. F. (2022). Reproducible brain-wide association studies require thousands of individuals. *Nature*, *603*(7902), 654–660. 10.1038/s41586-022-04492-9

Nosek, B. A., Beck, E. D., Campbell, L., Flake, J. K., Hardwicke, T. E., Mellor, D. T., van 't Veer, A. E., & Vazire, S. (2019). Preregistration Is Hard, And Worthwhile. *Trends in Cognitive Sciences*, *23*(10), 815–818. 10.1016/j.tics.2019.07.009

Pedregosa, F., Varoquaux, G., Gramfort, A., Michel, V., Thirion, B., Grisel, O., Blondel, M., Müller, A., Nothman, J., Louppe, G., Prettenhofer, P., Weiss, R., Dubourg, V., Vanderplas, J., Passos, A., Cournapeau, D., Brucher, M., Perrot, M., & Duchesnay, É. (2012). *Scikit-learn: Machine Learning in Python*. 10.48550/ARXIV.1201.0490

Poldrack, R. A., Huckins, G., & Varoquaux, G. (2020). Establishment of Best Practices for Evidence for Prediction: A Review. *JAMA Psychiatry*, *77*(5), 534. 10.1001/jamapsychiatry.2019.3671

Preprocessed Connectomes Project. (n.d.). Preprocessing with C-PAC. http://preprocessed-connectomes-project.org/abide/cpac.html

Prosperi, M., Guo, Y., Sperrin, M., Koopman, J. S., Min, J. S., He, X., Rich, S., Wang, M., Buchan, I. E., & Bian, J. (2020). Causal inference and counterfactual prediction in machine learning for actionable healthcare. *Nature Machine Intelligence*, *2*(7), 369–375. 10.1038/s42256-020-0197-y

Raykar, V. C., & Saha, A. (2015). Data Split Strategiesfor Evolving Predictive Models. In *Lecture Notes in Computer Science* (pp. 3–19). Springer International Publishing. 10.1007/978-3-319-23528-8_1

Riley, R. D., Debray, T. P. A., Collins, G. S., Archer, L., Ensor, J., van Smeden, M., & Snell, K. I. E. (2021). Minimum sample size for external validation of a clinical prediction model with a binary outcome. *Statistics in Medicine*, *40*(19), 4230–4251. 10.1002/sim.9025

Rosenberg, M. D., & Finn, E. S. (2022). How to establish robust brain–behavior relationships without thousands of individuals. *Nature Neuroscience*, *25*(7), 835–837. 10.1038/s41593-022-01110-9

Spisak, T. (2022). Statistical quantification of confounding bias in machine learning models. *GigaScience*, *11*. 10.1093/gigascience/giac082

Spisak, T., Bingel, U., & Wager, T. D. (2023). Multivariate BWAS can be replicable with moderate sample sizes. *Nature*, *615*(7951), E4–E7. 10.1038/s41586-023-05745-x

Spisak, T., Kincses, B., Schlitt, F., Zunhammer, M., Schmidt-Wilcke, T., Kincses, Z. T., & Bingel, U. (2020). Pain-free resting-state functional brain connectivity predicts individual pain sensitivity. *Nature Communications*, *11*(1). 10.1038/s41467-019-13785-z

Steyerberg, E. W., & Harrell, F. E. (2016). Prediction models need appropriate internal, internal–external, and external validation. *Journal of Clinical Epidemiology*, *69*, 245–247. 10.1016/j.jclinepi.2015.04.005

Street, W. N., Wolberg, W. H., & Mangasarian, O. L. (1993). Nuclear feature extraction for breast tumor diagnosis In R. S. Acharya & D. B. Goldgof (Eds.), *Biomedical Image Processing and Biomedical Visualization*. SPIE. 10.1117/12.148698

Sui, J., Jiang, R., Bustillo, J., & Calhoun, V. (2020). Neuroimaging-based Individualized Prediction of Cognition and Behavior for Mental Disorders and Health: Methods and Promises. *Biological Psychiatry*, *88*(11), 818–828. 10.1016/j.biopsych.2020.02.016

Thirion, B. (2023). On the statistics of brain/behavior associations. *Aperture Neuro*. 10.52294/51f2e656-d4da-457e-851e-139131a68f14

Van Essen, D. C., Smith, S. M., Barch, D. M., Behrens, T. E. J., Yacoub, E., & Ugurbil, K. (2013). The WU-Minn Human Connectome Project: An overview. *NeuroImage*, *80*, 62–79. 10.1016/j.neuroimage.2013.05.041

Varoquaux, G. (2018). Cross-validation failure: Small sample sizes lead to large error bars. *NeuroImage*, *180*, 68–77. 10.1016/j.neuroimage.2017.06.061

Varoquaux, G., & Cheplygina, V. (2022). Machine learning for medical imaging: methodological failures and recommendations for the future. *Npj Digital Medicine*, *5*(1). 10.1038/s41746-022-00592-y

Wolberg, W., Street, W. N., & Mangasarian, O. (1994). Breast cancer diagnosis and prognosis via linear programming.

Field Code Changed

Field Code Changed

Woo, C.-W., Chang, L. J., Lindquist, M. A., & Wager, T. D. (2017). Building better biomarkers: brain models in translational neuroimaging. *Nature Neuroscience*, *20*(3), 365–377. 10.1038/nn.4478

Yang, C., Kors, J. A., Ioannou, S., John, L. H., Markus, A. F., Rekkas, A., de Ridder, M. A. J., Seinen, T. M., Williams, R. D., & Rijnbeek, P. R. (2022). Trends in the conduct and reporting of clinical prediction model development and validation: a systematic review. *Journal of the American Medical Informatics Association*, *29*(5), 983–989. 10.1093/jamia/ocac002

Yu, A. C., Mohajer, B., & Eng, J. (2022). External Validation of Deep Learning Algorithms for Radiologic Diagnosis: A Systematic Review. *Radiology: Artificial Intelligence*, *4*(3). 10.1148/ryai.210064

 Click here to access/download;Personal Cover;Letter.docx

Dear Dr. Zauner,

We thank you and the reviewers for their time and the insightful and constructive assessment of our manuscript entitled ″External validation of machine learning models - registered models and adaptive sample splitting” and the opportunity to revise our manuscript accordingly.

We were pleased to read the reviewers’ overall positive feedback on our work and thankful to them for recognizing its importance and timeliness. We are especially grateful for the invaluable comments that highlighted areas where additional information was necessary. We have taken care to address these points in our revisions and feel that these really helped us improve both the quality and clarity of the manuscript.

In response to your request, we have added a new section detailing the availability of supporting code and requirements to the manuscript. Furthermore, we have indexed our software on bio.tools and Scicrunch, and updated the "Data and Code Availability" section to include a direct link to our analysis repository, which provides access to all necessary code for reproducing the analyses, plots, and machine learning model outputs presented in the manuscript.

Please find attached the latest revision of the manuscript and our point-by-point responses to the reviewers’ comments. We hope that the revisions and our accompanying responses will be sufficient to make our manuscript suitable for publication in GigaScience. We would be also glad to respond to any further questions and comments that you or the reviewers may have.

We are looking forward to hearing from you.

Yours sincerely,

Giuseppe Gallitto,
On behalf of all authors

Click here to access/download;Personal Cover;review_response_letter.docx

Reviewer #1: The manuscript discusses an interesting approach that seeks optimal data split for the pre-registration framework. The approach adaptively optimizes the balance between predictive performance of discovery set and sample size of external validation set. The approach is showcased on 4 applications, demonstrating advantage over traditional fixed data split (e.g., 80/20).

I generally enjoyed reading the manuscript. I believe pre-registration is one important tool for reproducible ML analysis and the ideology behind the proposed framework (investigating the balance between discovery power and validation power) is urgently needed. My main concerns are all around Fig. 3, which represents the core quantitative analysis but lacks many details.

We are thankful for the positive evaluation of our manuscript and for acknowledging its importance. We agree that the presentation of the main results of the manuscript indeed wasn't detailed enough. Below we outline how we addressed the issues raised by the reviewer.

1. Fig. 3 is mostly about external validation. What about training? For each n_total, which stopping rule is activated? What is the training accuracy? What does I_act look like? What is \hat{s_total}?

We fully agree that - next to examining the external validity of models - estimates of internal validity (i.e. cross-validated performance estimates during the model discovery phase) are also of high importance. This is especially true for the proposed technique, where the power of external validation is determined based on the internal validity estimates I_{act}. Thus, we have slightly revised our terminology for more clarity (replacing the term "training" with "discovery" and "test scores" to "external validation scores" throughout the manuscript) and added a new table (Table 1) that presents the requested details, including performance scores for the discovery phase. We also added a new figure in the supplementary material (fig. 12) that provides a graphical illustration of model performance during the discovery phase. To provide even more insights, we now present the relationship between the internally validated scores at the time of stopping (I_{act}), the corresponding external validation scores and sample sizes, for all 4 datasets in supplementary figures 8-11. The figures show a relatively good correspondence between internally and externally validated performance estimates with all splitting strategies.

Finally, we clarified that all stopping events with the adaptive splits approach shown on Fig 3. were induced by the power rule. Stopping was prevented if the actual sample size was lower than 12 (sample size rule; see also our responses to your comment 5). To keep the main analysis (presented on Fig. 3) as clean as possible, we decided to deactivate the performance rule, by setting the minimum relevant score (s_{min}) to 0. We apologize that this wasn't communicated clearly enough in the previous version. Now we provide a clear explanation of these details. For instance, in the Methods section we write:

“Note that this parameter configuration essentially switches off the performance rule for our main analysis (/s_{min}=0, but see Supplementary material, figure 7, for an analysis of the effect of the performance rule).” (Line 180 - 182)

Even though the performance rule was inactive in the analyses presented on Fig 3., we believe it still provides a useful option in our implementation: it allows us to trade a negligible decrease in model performance for more robust estimates of external validation or may even prevent the unnecessary use of resources by allowing early stopping in case of very well powered models. In the revised manuscript we illustrate this on supplementary figure 7 with our most highly powered dataset: the BCW dataset, by setting the sample size budget to maximum (n_total = 569) and using the performance rule to detect the plateauing of the learning curve with various minimum relevant score settings.

2. Results section states "the proposed adaptive splitting strategy always provided equally good or better predictive performance than the fixed splitting strategies (as shown by the 95% confidence intervals on Figure 3)". I'm confused by this because the blue curve is often below other methods in accuracy (e.g., comparing with 90/10 split in ABIDE and HCP).

Thank you for your comment, we fully agree that this was an overstatement and apologize for the mistake. We rephrased the sentence as follows:

“The proposed adaptive splitting strategy provided an external validation performance comparable to the alternative fixed splitting strategies, in all cases where the external validation was conclusive (statistically significant). Furthermore, in contrast to the investigated fixed splitting strategies, the proposed splitting strategy yields solid guarantees for the success of the external validation phase, independent of the sample size budget.” (Line 337 - 341)

3. Why does the half split have the lowest accuracy but the highest statistical power?

In the revised manuscript we discuss this phenomenon the following way:

“From the fixed splitting approaches, the half-split assigns the least samples from the total sample size budget to the training phase (50%). Thus, the resulting model is trained on less data than with other strategies, typically resulting in a smaller l_{act}. While this lower effect size should in general result in lower statistical power during the external validation phase, the half-split approach can counterbalance this with the larger sample size remaining for external validation. Our analysis shows, that this happens in almost all of the cases, hinting that in research scenarios where the expected predictive performance is low, researchers should either use the proposed adaptive splitting procedure, or aim for a relatively large pre-fixed external validation sample”. (Line 314 – 321)

We believe this is an interesting observation and are thankful for the reviewer for bringing it up.

4. How was the range of x-axis (n_total) selected? E.g., HCP has 1000 subjects, why was 240-380 chosen for analysis?

We apologize for not being clear about this in the previous version of the manuscript. Initially, we performed the analysis with all possible sample size budgets (with increments of 5), but we noticed that - depending on the power of the individual datasets and the used machine learning model – many of these simulation cases are not informative. When the sample size budget is too low, no meaningful prediction can be achieved. When the total sample size budget is too high, independent of the splitting ratio, we will stop at the "plateau" of the learning curve, where all splitting strategies will perform similarly good. These cases are not just uninformative but also uninteresting for realistic research scenarios, which – due to prior domain knowledge about the expected predictive performance - tend to employ sample sizes that fall into the critical range investigated in this study. Too small samples are clearly inadequate and too large samples are uneconomical, due to the diminishing returns as the learning curve saturates.

In the revised manuscript, we discuss this as follows:

"Thus, when reporting our results, we focused on the most realistic scenarios and omitted sample size budgets that were powered too low (neither of the splitting strategies leads to significant model performance) or too high (prediction performance plateaus with all splitting strategies) for any meaningful comparison between splitting strategies." (Line 301 - 304)

Furthermore, in the discussion we mention that with highly-powered studies, adaptive splitting may not provide advantages to fixed splits:

"The benefit of adaptively splitting the data acquisition for training and validation provides the largest benefit in lower sample size regimes. In case of larger total sample size budgets, the fixed Pareto split (20-80%) provided also good results, giving similar external validation performances to adaptive splitting, without having to repeatedly re-train the model during data acquisition." (Line 387 - 390)

5. The lowest n_total for BCW and IXI is approximately 50. If n_act starts from 10% of n_total, how is it possible to train (nested) cross-validation on 5 samples or so?

Thank you for raising this important point. The situation you describe is addressed by a "min_sample_size" parameter, which is part of the stopping rule configuration (that can be set-up in the adaptivesplit package's setting.conf configuration file). For all datasets, we specified a minimum sample size of 12. Setting the sample size rule to this value not only avoids "false stops" in the beginning where the learning curve is still noisy, but also

ensures that the chosen cross-validation scheme can always be applied. In the revised manuscript we point this out explicitly in the methods section:

"Note that this parameter configuration" … "ensures that even in case of very small simulated sample size budgets, the training sample is suitable for cross-validation (vmin=12)." (Line 180 - 184)

6. How can this be applied to retrospective data or secondary data analysis where the collection is finished?

Thank you for your question. While our approach was primarily designed with prospective data acquisition in mind and is particularly suited for use in conjunction with pre-registration protocols, it can be applied to retrospective data without modifications.

In detail, with retrospective data, one can simply "simulate" a prospective data acquisition by involving more and more data into the model discovery until the stopping rule activates (as we did in the present study). It is essential to guarantee in this case, however, that the model training process remains unbiased and no information leaks from the actual held-out part of the whole dataset into the training process (e.g. through globally executed preprocessing steps). As long as these considerations are met, the method can be effectively employed in retrospective settings.

7. Is there a guidance on the minimum sample size that is required to perform such an auto-split analysis? It is surprising that the authors think the two studies with n=35 and n=38 are good examples of training generalizable ML models. It is generally hard to believe any ML analysis can be done on such low sample sizes with thousands of rs-fMRI features. By the way, I believe n=25 in Kincses 2024 if I read it correctly.

Thank you for the important comment. While in general we share the reviewer's skepticism regarding training complex ML-models in very small samples, we must mention that there are notable examples of models that were trained on small samples but were found to generalize well to new data. For instance the Neurologic Pain Signature (Wager et al., 2013) was initially developed on 20 samples and since then demonstrated to generalize very well in a series of external validation studies, e.g. an individual-participant meta-analysis including 20 independent studies and more than 600 participants (Zunhammer et al, 2018).
Nevertheless, we agree that small sample sizes are, in general, problematic and would like to point out that the proposed approach is not meant to solve this issue. We believe that any general rules-of-thumb on minimal sample sizes may become misleading and the question must be carefully considered from study to study, weighing on the specific characteristics of the data and the prediction task (as discussed e.g. in Spisak et al, 2023). However, the reviewer is correct that the proposed approach may have a special role in small-sample model discovery studies; it may be most useful in exactly in these cases by

supporting the development of credible, externally validated “prototype models” that can be further developed and validated in the biomarker development process.

Thank you furthermore for spotting the typos regarding the sample sizes in the two cited studies (line 108 and 109 of the manuscript). The sample size in Spisak et al. 2020 during discovery phase is of 39 (not 35) and the sample size for Kincses et al is 25 and not 38 (after exclusions). We corrected the typos in the main text.

Wager, T. D., Atlas, L. Y., Lindquist, M. A., Roy, M., Woo, C. W., & Kross, E. (2013). An fMRI-based neurologic signature of physical pain. New England Journal of Medicine, 368(15), 1388-1397.

Zunhammer M, Bingel U, Wager TD, Placebo Imaging Consortium. Placebo effects on the neurologic pain signature: a meta-analysis of individual participant functional magnetic resonance imaging data. JAMA neurology. 2018 Nov 1;75(11):1321-30.

Spisak T, Bingel U, Wager TD. Multivariate BWAS can be replicable with moderate sample sizes. Nature. 2023 Mar 9;615(7951):E4-7.

Reviewer #2: External validation of machine learning models - registered models and adaptive sample splitting Gallitto et al.

The Manuscript describes a methodology and algorithm aimed at better choosing a train-test validation split of data for scikit-learn models. A python package, adaptivesplit, was built as part of this MS as a tool for others to use. The package is proposed to be used together with a suggested workflow to integrate an approach invoking registered models as a full design for better prospective modelling studies. Finally, the work is evaluated on four alternative publicly available datasets of health research data and comprehensive results are presented.

There is a trade-off in the split between the amount of sample data to be used for training and the amount of data to use for validation. Ideally the content of each must be balanced in order for the trained model to be representative and equally for the validation set to be representative. This manuscript is therefore very timely due to the large increase in the use of AI models and provides important information and methodology.

This reviewer does not have the specific expertise to provide detailed comments on the statistical rule methods.

We are thankful to the reviewer for highlighting the timeliness of the work.

1. The Python implementation of the "adaptivesplit" package is described as available on GitHub (Gallitto et al., n.d.). One of the major points of the paper is to provide the python package "adaptivesplit", however, this package does not have a clear hyperlink, and is not found by simple google searches, and it appears is not yet available. It is therefore not possible to evaluate it at present. There is a website found available with a preprint of this MS after further google searches, https://pni-lab.github.io/adaptivesplit/ however, adaptive split is here shown as an interactivate jupyter-type notebook example and not as a python library code. Therefore, it is not

clear how available the package is for others' use. Can the authors comment on the code availability?

Thank you for testing the availability and findability of the "adaptivesplit" Python package. While at the time of review, the page was probably not yet indexed by web-search engines, at the time of writing this revision, the package (https://github.com/pni-lab/adaptivesplit) seems to be findable through search engines. Based on your comment, we have now updated the repository's README file with clear installation instructions. We apologize for the lack of clarity in the previous version of the repository. Additionally, we provide an interactive Jupyter notebook example to demonstrate the package's functionality.

All resources can now be accessed via our GitHub repository at the following links:
- Documentation: https://pni-lab.github.io/adaptivesplit/
- Package repository: https://github.com/pni-lab/adaptivesplit
- Analysis code repository: https://github.com/pni-lab/AdaptiveSplitAnalysis
- Pre-print: https://pni-lab.github.io/AdaptiveSplitAnalysis/

2. Apart from the 80:20 Pareto split of train-test data, other splits are commonly used in ratios such as 75:25 (the scikit-learn default split if ratio is unspecified), and 70:30. Also the cross-validation strategy with train-test-validation split 60:20:20, yet these strategies have not been mentioned or included in the figures such as Fig 3. The splits provided in the figure and discussed are 50:50, 80:20 and 90:10 only. Could the authors discuss alternative split ratios?

Thank you for your valuable insights regarding alternative split ratios. We have re-run the whole analysis with the suggested split ratios.
As showing these new fixed-split results on Fig 3. Would have madxe the figure too crowded, we present these new results in the supplementary figure 13. The analysis code and stored results have also been updated to include the new splits (see: https://github.com/pni-lab/AdaptiveSplitAnalysis).
The additional fixed splitting ratios complement our results and further support our main conclusions. There are always sample size budgets, with which fixed splits will lead to an inconclusive external validation phase and, in all cases with a conclusive external validation, fixed splits provide results that are comparable or worse to those provided by adaptivesplit.

In the revised manuscript, we discuss different splits and refer to these supplementary results as follows:

"In addition to the Pareto, half-split, and 90-10% splitting strategies, we also evaluated alternative split ratios (75-25% and 70-30%), which are commonly used in the literature. The 75-25% split demonstrated performance comparable to the Pareto and adaptive splitting techniques, although, similarly to Pareto, it struggled to achieve statistical significance at smaller sample sizes. In contrast, the 70-30% split exhibited good

statistical significance at the cost of lower overall performance, comparable to the trend observed with the half-split strategy (see Supplementary material, Figure 13)." (Line 326 – 331)
