# Supplementary material for: External validation of machine learning models—registered models and adaptive sample splitting
Source: Gigascience. 2025 May 14;14:giaf036. doi: 10.1093/gigascience/giaf036 (PMC12077397; doi:10.1093/gigascience/giaf036)
Supplement: giaf036_Authors_Response_To_Reviewer_Comments_Revision_2 [file giaf036_authors_response_to_reviewer_comments_revision_2.pdf]

Author's Response To Reviewer Comments

Dear Dr. Zauner,

The requested changes have been incorporated into the manuscript.

All ORCID IDs listed at the beginning of the manuscript are accurate, and datasets without identifiers have been cited as internet sources. Additionally, we have cited the https://github.com/pni-lab/AdaptiveSplitAnalysis support repository both as a GitHub repository and in Software Heritage. The DOME annotations and the Software Heritage archival version of the main repository are now also referenced in the text.

Thank you for taking the time to review our manuscript. Please don't hesitate to contact us if further revisions are needed.

Kind Regards,
Giuseppe Gallitto
On behalf of all authors

Editorial Manager®

Cookie Preference Center

We use cookies which are necessary to make our site work. We may also use additional cookies to analyze, improve and personalize our content and your digital experience. For more information, see our Cookie Policy.

You may choose not to allow some types of cookies. However, blocking some types may impact your experience of our site and the services we are able to offer. See the different category headings below to find out more or change your settings.

Allow all

Manage Consent Preferences

Strictly Necessary Cookies

Always active

These cookies are necessary for the website to function and cannot be switched off in our systems. They are usually only set in response to actions made by you which amount to a request for services, such as setting your privacy preferences, logging in or filling in forms. You can set your browser to block or alert you about these cookies, but some parts of the site will not then work. These cookies do not store any personally identifiable information.

Functional Cookies

These cookies enable the website to provide enhanced functionality and personalisation. They may be set by us or by third party providers whose services we have added to our pages. If you do not allow these cookies then some or all of these services may not function properly.

Performance Cookies

These cookies allow us to count visits and traffic sources so we can measure and improve the performance of our site. They help us to know which pages are the most and least popular and see how visitors move around the site.

Targeting Cookies

These cookies may be set through our site by our advertising partners. They may be used by those companies to build a profile of your interests and show you relevant adverts on other sites. If you do not allow these cookies, you will experience less targeted advertising.

Cookie List

Clear

Apply Cancel
Consent Leg.Interest

Confirm my choices

Powered by onetrust
