# Supplementary material for: External validation of machine learning models—registered models and adaptive sample splitting
Source: Gigascience. 2025 May 14;14:giaf036. doi: 10.1093/gigascience/giaf036 (PMC12077397; doi:10.1093/gigascience/giaf036)
Supplement: giaf036_Reviewer_1_Report_Original_Submission — Qingyu Zhao -- 7/30/2024 [file giaf036_reviewer_1_report_original_submission.pdf]

Reviewer Report

Title: External validation of machine learning models - registered models and adaptive sample splitting

Version: Original Submission **Date:** 7/30/2024

Reviewer name: Qingyu Zhao

Reviewer Comments to Author:

The manuscript discusses an interesting approach that seeks optimal data split for the pre-registration framework. The approach adaptively optimizes the balance between predictive performance of discovery set and sample size of external validation set. The approach is showcased on 4 applications, demonstrating advantage over traditional fixed data split (e.g., 80/20).
I generally enjoyed reading the manuscript. I believe pre-registration is one important tool for reproducible ML analysis and the ideology behind the proposed framework (investigating the balance between discovery power and validation power) is urgently needed. My main concerns are all around Fig. 3, which represents the core quantitative analysis but lacks many details.
1. Fig. 3 is mostly about external validation. What about training? For each n_total, which stopping rule is activated? What is the training accuracy? What does l_act look like? What is \hat{s_total}?
2. Results section states "the proposed adaptive splitting strategy always provided equally good or better predictive performance than the fixed splitting strategies (as shown by the 95% confidence intervals on Figure 3)". I'm confused by this because the blue curve is often below other methods in accuracy (e.g., comparing with 90/10 split in ABIDE and HCP).
3. Why does the half split have the lowest accuracy but the highest statistical power?
4. How was the range of x-axis (n_total) selected? E.g., HCP has 1000 subjects, why was 240-380 chosen for analysis?
5. The lowest n_total for BCW and IXI is approximately 50. If n_act starts from 10% of n_total, how is it possible to train (nested) cross-validation on 5 samples or so?
Two other general comments are
1. How can this be applied to retrospective data or secondary data analysis where the collection is finished?
2. Is there a guidance on the minimum sample size that is required to perform such an auto-split analysis? It is surprising that the authors think the two studies with n=35 and n=38 are good examples of training generalizable ML models. It is generally hard to believe any ML analysis can be done on such low sample sizes with thousands of rs-fMRI features. By the way, I believe n=25 in Kincses 2024 if I read it correctly.

Methods

Are the methods appropriate to the aims of the study, are they well described, and are necessary controls included? Choose an item.

Conclusions

Are the conclusions adequately supported by the data shown? Choose an item.

Reporting Standards

Does the manuscript adhere to the journal's guidelines on minimum standards of reporting? Choose an item.

Choose an item.

Statistics

Are you able to assess all statistics in the manuscript, including the appropriateness of statistical tests used? Choose an item.

Quality of Written English

Please indicate the quality of language in the manuscript: Choose an item.

Declaration of Competing Interests

Please complete a declaration of competing interests, considering the following questions:

- Have you in the past five years received reimbursements, fees, funding, or salary from an organisation that may in any way gain or lose financially from the publication of this manuscript, either now or in the future?
- Do you hold any stocks or shares in an organisation that may in any way gain or lose financially from the publication of this manuscript, either now or in the future?
- Do you hold or are you currently applying for any patents relating to the content of the manuscript?
- Have you received reimbursements, fees, funding, or salary from an organization that holds or has applied for patents relating to the content of the manuscript?
- Do you have any other financial competing interests?
- Do you have any non-financial competing interests in relation to this paper?

If you can answer no to all of the above, write 'I declare that I have no competing interests' below. If your reply is yes to any, please give details below.

I declare that I have no competing interests

I agree to the open peer review policy of the journal. I understand that my name will be included on my report to the authors and, if the manuscript is accepted for publication, my named report including any attachments I upload will be posted on the website along with the authors' responses. I agree for my

report to be made available under an Open Access Creative Commons CC-BY license (http://creativecommons.org/licenses/by/4.0/). I understand that any comments which I do not wish to be included in my named report can be included as confidential comments to the editors, which will not be published.

Choose an item.

To further support our reviewers, we have joined with Publons, where you can gain additional credit to further highlight your hard work (see: https://publons.com/journal/530/gigascience). On publication of this paper, your review will be automatically added to Publons, you can then choose whether or not to claim your Publons credit. I understand this statement.

Yes Choose an item.
