# Supplementary material for: External validation of machine learning models—registered models and adaptive sample splitting
Source: Gigascience. 2025 May 14;14:giaf036. doi: 10.1093/gigascience/giaf036 (PMC12077397; doi:10.1093/gigascience/giaf036)
Supplement: giaf036_Reviewer_2_Report_Original_Submission — Lisa Crossman -- 8/29/2024 [file giaf036_reviewer_2_report_original_submission.pdf]

Reviewer Report

Title: External validation of machine learning models - registered models and adaptive sample splitting

Version: Original Submission **Date:** 8/29/2024

Reviewer name: Lisa Crossman

Reviewer Comments to Author:

External validation of machine learning models - registered models and adaptive sample splitting
Gallito et al.
The Manuscript describes a methodology and algorithm aimed at better choosing a train-test validation split of data for scikit-learn models. A python package, adaptivesplit, was built as part of this MS as a tool for others to use. The package is proposed to be used together with a suggested workflow to integrate an approach invoking registered models as a full design for better prospective modelling studies. Finally, the work is evaluated on four alternative publicly available datasets of health research data and comprehensive results are presented.
There is a trade-off in the split between the amount of sample data to be used for training and the amount of data to use for validation. Ideally the content of each must be balanced in order for the trained model to be representative and equally for the validation set to be representative. This manuscript is therefore very timely due to the large increase in the use of AI models and provides important information and methodology.
This reviewer does not have the specific expertise to provide detailed comments on the statistical rule methods.
Main Suggested Revision:
1. The Python implementation of the "adaptivesplit" package is described as available on GitHub (Gallitto et al., n.d.).
One of the major points of the paper is to provide the python package "adaptivesplit", however, this package does not have a clear hyperlink, and is not found by simple google searches, and it appears is not yet available. It is therefore not possible to evaluate it at present.
There is a website found available with a preprint of this MS after further google searches, https://pni-lab.github.io/adaptivesplit/ however, adaptive split is here shown as an interactivate jupyter-type notebook example and not as a python library code. Therefore, it is not clear how available the package is for others' use. Can the authors comment on the code availability?
Minor comments:
1. Apart from the 80:20 Pareto split of train-test data, other splits are commonly used in ratios such as 75:25 (the scikit-learn default split if ratio is unspecified), and 70:30. Also the cross-validation strategy with train-test-validation split 60:20:20, yet these strategies have not been mentioned or included in the figures such as Fig 3. The splits provided in the figure and discussed are 50:50, 80:20 and 90:10 only. Could the authors discuss alternative split ratios?

Methods

Are the methods appropriate to the aims of the study, are they well described, and are necessary controls included? Choose an item.

Conclusions

Are the conclusions adequately supported by the data shown? Choose an item.

Reporting Standards

Does the manuscript adhere to the journal's guidelines on minimum standards of reporting? Choose an item.

Choose an item.

Statistics

Are you able to assess all statistics in the manuscript, including the appropriateness of statistical tests used? Choose an item.

Quality of Written English

Please indicate the quality of language in the manuscript: Choose an item.

Declaration of Competing Interests

Please complete a declaration of competing interests, considering the following questions:

- Have you in the past five years received reimbursements, fees, funding, or salary from an organisation that may in any way gain or lose financially from the publication of this manuscript, either now or in the future?
- Do you hold any stocks or shares in an organisation that may in any way gain or lose financially from the publication of this manuscript, either now or in the future?
- Do you hold or are you currently applying for any patents relating to the content of the manuscript?
- Have you received reimbursements, fees, funding, or salary from an organization that holds or has applied for patents relating to the content of the manuscript?
- Do you have any other financial competing interests?
- Do you have any non-financial competing interests in relation to this paper?

If you can answer no to all of the above, write 'I declare that I have no competing interests' below. If your reply is yes to any, please give details below.

I declare that I have no competing interests

I agree to the open peer review policy of the journal. I understand that my name will be included on my report to the authors and, if the manuscript is accepted for publication, my named report including any attachments I upload will be posted on the website along with the authors' responses. I agree for my report to be made available under an Open Access Creative Commons CC-BY license (http://creativecommons.org/licenses/by/4.0/). I understand that any comments which I do not wish to be included in my named report can be included as confidential comments to the editors, which will not be published.

Choose an item.

To further support our reviewers, we have joined with Publons, where you can gain additional credit to further highlight your hard work (see: https://publons.com/journal/530/gigascience). On publication of this paper, your review will be automatically added to Publons, you can then choose whether or not to claim your Publons credit. I understand this statement.

Yes Choose an item.
